# A mechanically interlocked molecular system programmed for the delivery of an anticancer drug†
†Electronic supplementary information (ESI): Experimental conditions and procedures, syntheses and compounds characterizations (^1^H, ^13^C and 2D NMR spectroscopic analyses and mass spectrometry data) as well as biological experiments. See DOI: 10.1039/c5sc00648a



**DOI:** 10.1039/c5sc00648a

**Published:** 2015-02-25

**Authors:** Romain Barat, Thibaut Legigan, Isabelle Tranoy-Opalinski, Brigitte Renoux, Elodie Péraudeau, Jonathan Clarhaut, Pauline Poinot, Antony E. Fernandes, Vincent Aucagne, David A. Leigh, Sébastien Papot

**Affiliations:** a Université de Poitiers , UMR-CNRS 7285 , Institut de Chimie des Milieux et des Matériaux de Poitiers , groupe « Systèmes Moléculaires Programmés » , 4 rue Michel Brunet, TSA 51106 , 86073 Poitiers , France . Email: sebastien.papot@univ-poitiers.fr; b Université de Poitiers , CNRS ERL 7368 , 1 rue Georges Bonnet, TSA 51106 , 86073 Poitiers , France; c CHU de Poitiers , 2 rue de la Milétrie, CS 90577 , 86021 Poitiers , France; d Université de Poitiers , UMR-CNRS 7285 , Institut de Chimie des Milieux et des Matériaux de Poitiers , Equipe Eau, Géochimie Organique, Santé (EGS) , 4 rue Michel Brunet, TSA 51106 , 86073 Poitiers , France; e Institute of Condensed Matter and Nanoscience , Université catholique de Louvain , place Croix du Sud , 1348 Louvain-la-Neuve , Belgium; f Centre de Biophysique Moléculaire , CNRS , rue Charles Sadron , 45071 Orléans Cedex 2 , France; g School of Chemistry University of Manchester , Oxford road , Manchester MP13 9PL , UK

## Abstract

The development of mechanically interlocked molecular systems programmed to operate autonomously in biological environments is an emerging field of research with potential medicinal applications. Within this framework, functional rotaxane- and pseudorotaxane-based architectures are starting to attract interest for the delivery of anticancer drugs, with the ultimate goal to improve the efficiency of cancer chemotherapy. Here, we report an enzyme-sensitive [2]-rotaxane designed to release a potent anticancer drug within tumor cells. The molecular device includes a protective ring that prevents the premature liberation of the drug in plasma. However, once located inside cancer cells the [2]-rotaxane leads to the release of the drug through the controlled disassembly of the mechanically interlocked components, in response to a determined sequence of two distinct enzymatic activations. Furthermore, *in vitro* biological evaluations reveal that this biocompatible functional system exhibits a noticeable level of selectivity for cancer cells overexpressing β-galactosidase.

## Abstract 2

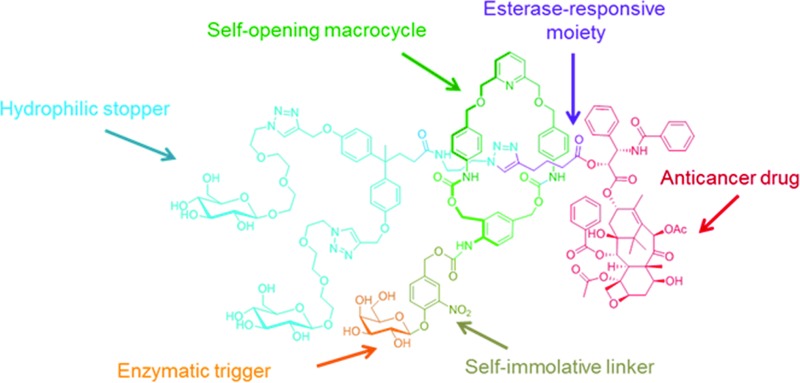
The development of mechanically interlocked molecular systems programmed to operate autonomously in biological environments is an emerging field of research with potential medicinal applications.

## Introduction

Over the past two decades, the design of functional interlocked molecules^1^ programmed to perform specific tasks in response to an external stimulus has received considerable attention.^2–9^ In particular, much effort has been devoted to control the dynamic behavior of mechanically interlocked architectures with the ultimate goal to develop molecular motors and machines for nanotechnological applications.^2^ More recently, rotaxane- and pseudorotaxane-based devices designed to interact with biological systems have started to emerge.^3–9^ Mechanized mesoporous silica nanoparticles coated with “nanovalves”,^5–7^ biodegradable polyrotaxanes^8^ and enzyme-responsive rotaxane-based propeptides^9^ have been proposed as potential anticancer drug carriers. However, the development of autonomous interlocked systems capable of transporting a potent drug in a stable non-toxic form, launching its biological activity once located inside cancer cells, still remains highly challenging.

**Fig. 1:**
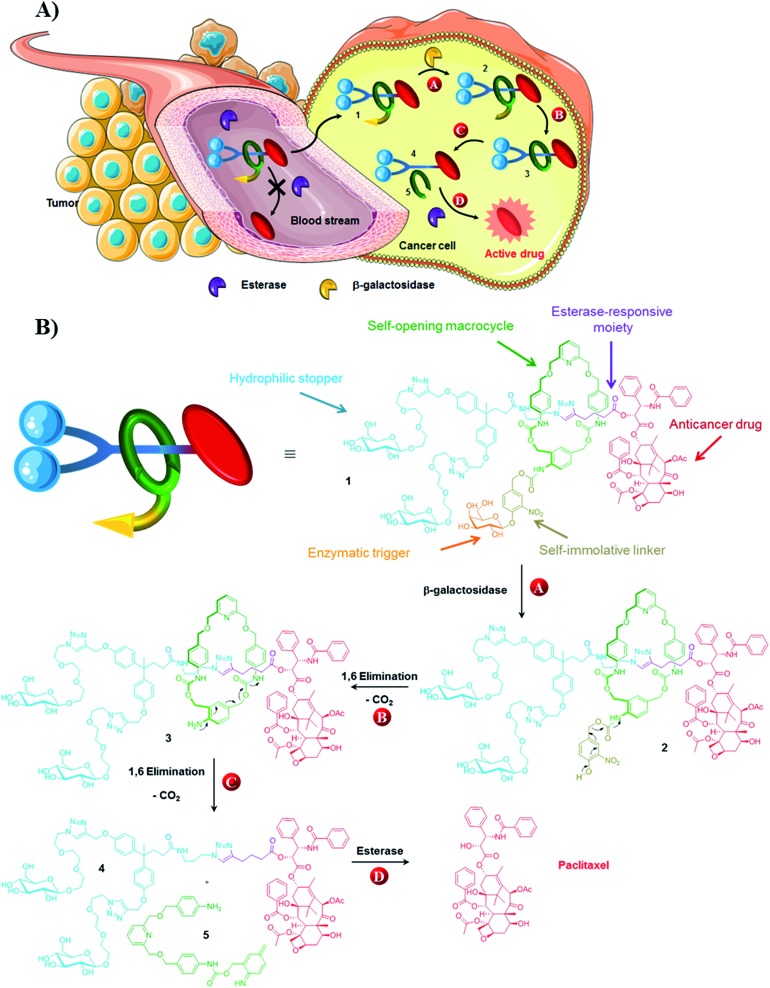
(A) The principle of the intracellular drug delivery with functional interlocked system **1**. When in the blood stream, rotaxane **1** does not release the drug due to the presence of the protective ring that prevents hydrolysis of the esterase-sensitive moiety. Once inside cancer cells, the process of drug release is initiated by the activation of the galactoside trigger by intracellular β-galactosidase (step A). This is followed by a spontaneous sequence of reactions that leads to opening of the protective ring and the concomitant disassembly of the interlocked architecture (steps B and C). As a result the ester linkage of the thread becomes accessible to intracellular esterases that induce the liberation of the active drug (step D). (B) Structure of rotaxane **1** and the paclitaxel release mechanism.

Here we report on the design, synthesis and operation of a novel biocompatible [2]-rotaxane **1** programmed for the intracellular delivery of a potent anticancer agent while preventing its premature liberation in plasma (Fig. 1). To achieve such a task in an autonomous manner, the molecular delivery system has built into its structure a latent “chemical program” that pilots the process of drug release through the disassembly of the mechanically interlocked components, in response to a determined sequence of enzymatic activations and chemical self-immolations. The functionality of rotaxane **1** relies on the association of several distinct units, including an enzymatic trigger, a self-immolative linker, a self-opening macrocycle, a hydrophilic stopper and an esterase-sensitive thread linked to the C2′–OH position of paclitaxel (Fig. 1). With this design, the macrocycle plays the role of a temporary molecular shield protecting the ester bond from hydrolysis by plasmatic esterases, thereby avoiding the release of the active drug in the bloodstream. Once inside cancer cells, activation of the galactoside trigger by β-galactosidase (Fig. 1A and B, step A) will initiate a sequence of chemical reactions leading ultimately to the decomposition of the interlocked architecture through the controlled opening of the protective ring (Fig. 1A and B, steps B and C). Unmasked in this way, the ester bond of the free thread then becomes accessible to intracellular esterases, allowing the release of paclitaxel within the cells (Fig. 1A and B, step D). The demonstration that an enzyme-sensitive [2]-rotaxane can be programmed to accomplish a specific task in an autonomous manner under the condition prevailing within living cells opens the way for the development of functional interlocked systems for triggered biological applications.

## Results and discussion

### Synthesis of rotaxane **1**

The key step in the synthesis of **1** relies on the construction of the interlocked architecture *via* the copper(i)-catalysed azide-alkyne 1,3-cycloaddition (CuAAC) active-metal template strategy.^10–12^

**Fig. 2:**
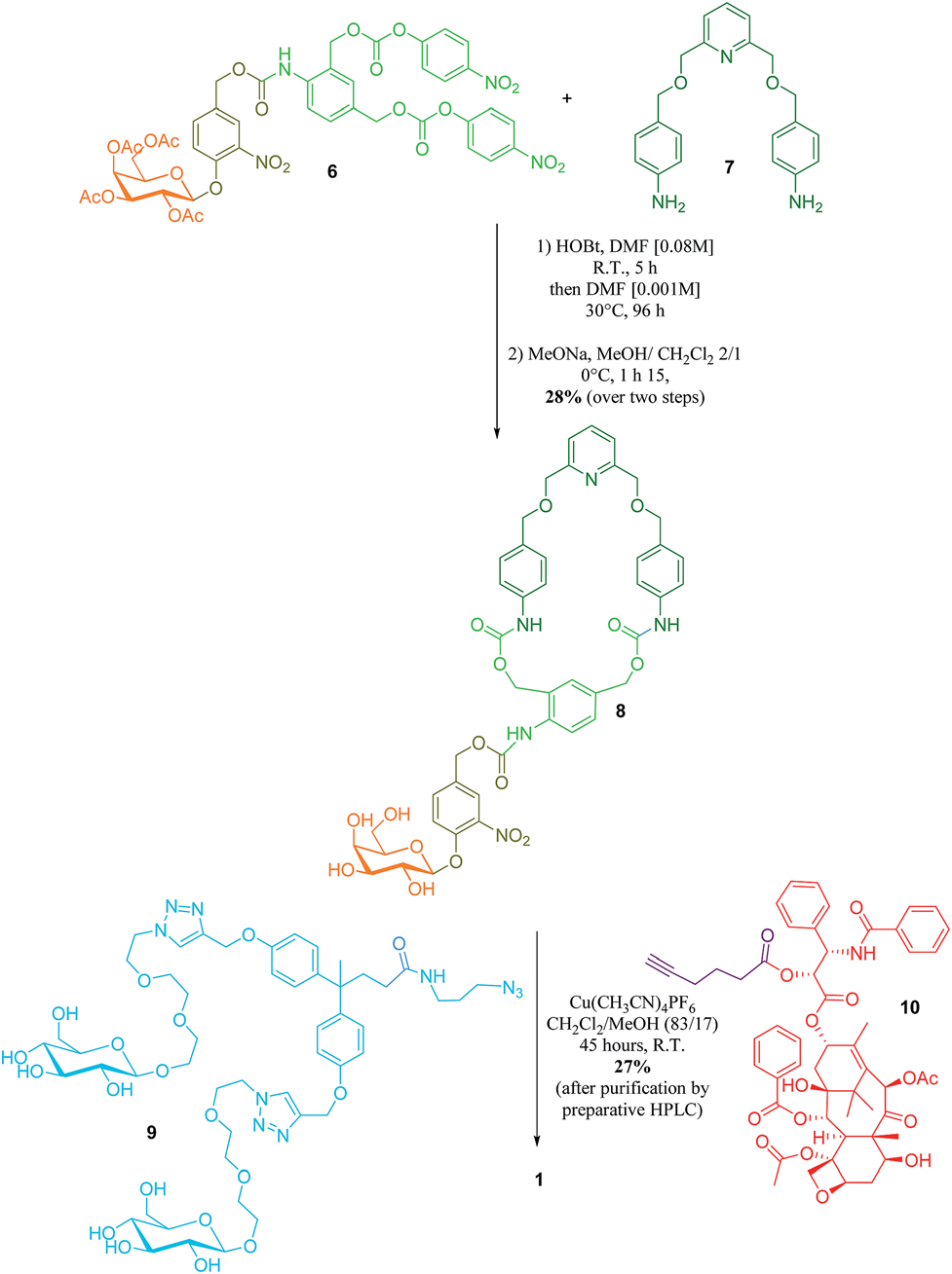
Synthesis of rotaxane **1**.

Coordination of Cu(i) to the endotopic pyridine-containing macrocycle **8** allows azide **9** and alkyne **10** to bind to the metal catalyst in such a way that formation of the triazole thread occurs predominantly within the cavity of the macrocycle, leading to the rotaxane architecture (Fig. 2).

Galactosylated ring **8** was prepared from biscarbonate **6** and dianiline **7** (see ESI†). The macrocycle was treated with azide **9**, alkyne **10** and Cu(CH_3_CN)_4_PF_6_ for 45 hours at room temperature in CH_2_Cl_2_/CH_3_OH (87/13) to afford [2]-rotaxane **1** as a mixture of two diastereoisomers (the off-centre urethane group of the macrocycle means that threading of the unsymmetrical axle through the ring in different directions produces nonidentical structures) in 27% yield after purification by preparative HPLC. The rotaxane was perfectly stable over several days in solution demonstrating that paclitaxel and the hydrophilic bisphenol-based moiety are bulky enough to act as stoppers, thereby avoiding premature disassembly of the interlocked architecture.

### Operation of functional interlocked system **1**

Paclitaxel is a potent antimitotic agent employed clinically for the treatment of several malignancies including ovarian, breast and lung cancer.^13^ However, the antitumor efficacy of this molecule is limited by its systemic toxicity and poor aqueous solubility. Thus, the derivatization of paclitaxel in the form of water soluble non-toxic prodrugs have been suggested as a valuable alternative to enhance the therapeutic index of this compound.^14^ The vast majority of paclitaxel prodrugs have been designed by introducing a promoiety to the C2′–OH position of the drug through an ester linkage. Since this hydroxyl group is crucial for the bioactivity,^15^ such derivatives are usually less toxic than paclitaxel. Furthermore, C2′–OH esters can be readily cleaved by various hydrolytic enzymes to restore the anticancer activity of the free drug that is a requisite property for a prodrug. This relatively high reactivity represents a significant drawback in this approach due to the rapid hydrolysis of these prodrugs by plasmatic esterases preventing them reaching cancer cells. In contrast, by reducing the accessibility of the C2′–OH ester bond, the macrocycle of rotaxane **1** should limit the esterase-mediated degradation of the thread and the subsequent liberation of paclitaxel in plasma.

The stability of rotaxane **1** was examined in rat plasma at 37 °C and compared to that of the corresponding thread **4**. Under these conditions, prodrug **4** was rapidly cleaved leading to the release of the free drug, confirming the instability of C2′–OH esters of paclitaxel in plasma (see ESI†). In contrast, the interlocked system **1** was perfectly stable over a 48 hour period without any detectable liberation of the drug. This demonstrates that encapsulation of the thread **4** within a rotaxane structure prevents its esterase-mediated hydrolysis in plasma. Furthermore, compound **1** was readily soluble in plasma at a concentration of 3.3 × 10^–2^ mol L^–1^ whereas the aqueous solubility of paclitaxel is 1.1 × 10^–5^ mol L^–1^.^16^ The increased solubility, which probably results from the presence of the galactoside trigger and the Glc-TEG-based stopper,^17^ is significant since in the clinic the poor aqueous solubility of paclitaxel means that it has to be administered in a vehicle containing ethanol and Cremophor EL.

We next investigated the release mechanism of the thread **4** from the interlocked system **1** in the presence of β-galactosidase (Fig. 1B). This enzyme is present only in vanishingly small concentrations in serum and its activity is overwhelmingly intracellular. As a result, activation of the enzymatic trigger of the functional interlocked system **1** should take place exclusively inside living cells. Furthermore, since the expression of β-galactosidase is much higher in several tumor types compared to normal tissues,^18–20^ system **1** should be activated preferentially within cancer cells exhibiting elevated levels of this enzyme.

**Fig. 3:**
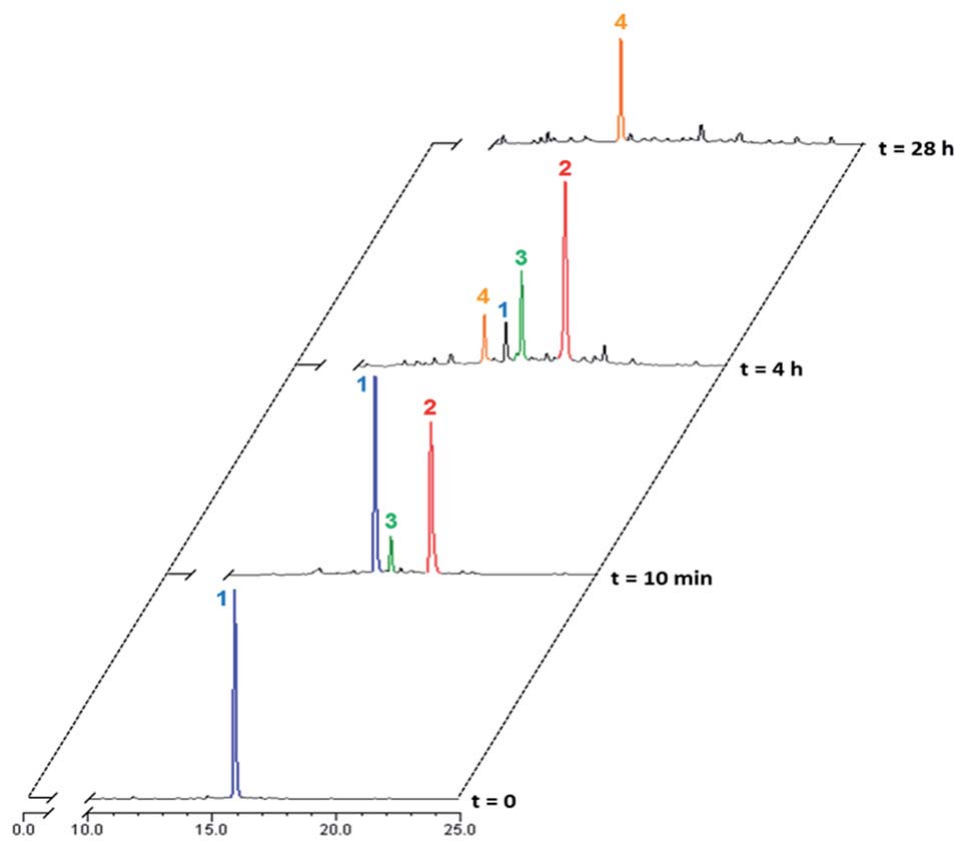
Enzymatic hydrolysis of rotaxane **1** with *E. coli* β-galactosidase in phosphate buffer (0.02 M, pH 7.2, 37 °C) monitored by HPLC at *t* = 0, *t* = 10 min, *t* = 4 h and *t* = 28 h. Retention times: **1** (15.87 min), **2** (18.22 min), **3** (16.59 min), **4** (14.98 min).

Rotaxane **1** was incubated with the enzyme in phosphate buffer (pH 7.2, 0.02 M) at 37 °C and the evolution of the mixture over time monitored by HPLC/HRMS (Fig. 3). The chromatograms showed the rapid emergence (*t* = 10 min) of two new peaks with *m*/*z* 1387.0735 and 1311.5593 ([M + 2H]^2+^), which correspond respectively to the degalactosylated rotaxane **2** and the aniline **3** resulting from the self-immolation of the nitro-benzyloxycarbonyl linker.^21,22^ Rotaxane **3** then spontaneously decomposed, enabling the opening of the macrocycle and the subsequent release of the free thread **4** (*m*/*z* 1034.4501, [M + 2H]^2+^, *t* = 4 h). The opening mechanism of the macrocycle was confirmed by HRMS detection of the unstable azaquinone methide **5** (*m*/*z* 511.2340, [M + H]^+^) that degraded rapidly in the medium.^23^ After 28 hours under these conditions, the interlocked system **1** as well as the intermediates **2** and **3** completely disappeared from the mixture, whilst the amount of thread **4** increased and then remained stable once all of **1–3** had been consumed.

### Biological evaluation

**Fig. 4:**
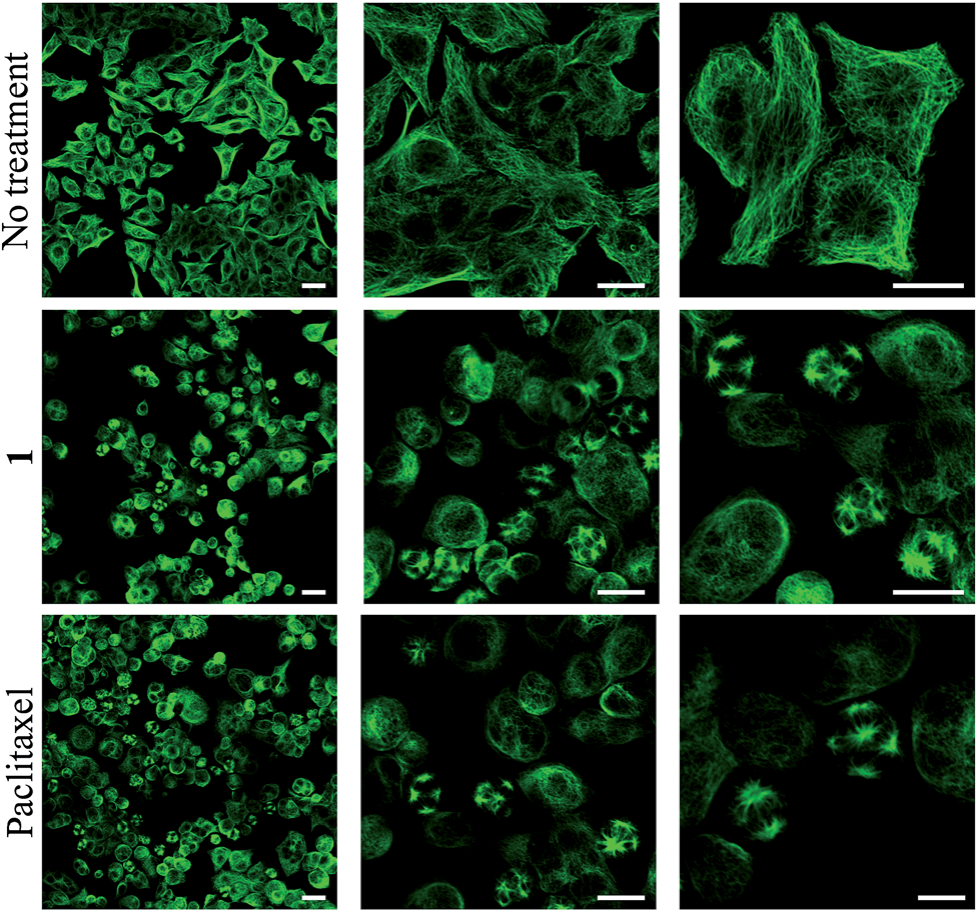
α-Tubulin immunodetection by confocal microscopy in KB cells (human mouth epidermal carcinoma) when non-treated, treated for 48 hours with either 100 nM of rotaxane **1** or 25 nM of paclitaxel. Scale bar: 25 μm.

To obtain evidence for the release of paclitaxel inside cancer cells, we first examined the inhibition of tubulin polymerization, the mechanism by which paclitaxel exerts its anticancer activity. As shown by confocal microscopy imaging, incubation of rotaxane **1** with KB tumor cells disturbed the microtubule network like paclitaxel, while this was not observed when cells were not treated with **1** (Fig. 4). Since the free C2′–OH of paclitaxel is essential for its bioactivity,^15^ this effect can be attributed to the liberation of the active drug within the tumor cells.

This result also correlates with the high cytotoxicity observed for compound **1** when incubated for 48 hours with KB, H661 and MDA-MB-231 cells, exhibiting IC_50_ values of 88.7, 144.0 and 192.3 nM respectively (Table 1). However, paclitaxel was from 3.9 to 4.7-fold more toxic on the same cancer cell lines with IC_50_ of 19.4, 37.3 and 41.2 nM. This difference of cytotoxicity may be a result of the higher hydrophilicity of **1** reducing its passive penetration through the cell membrane. Nevertheless, the lower antiproliferative activity of rotaxane **1** is outweighed by the gain in selectivity for cancer cells overexpressing β-galactosidase. We measured the activity of **1** on siRNA-transfected KB cell line with a 60% reduced expression of β-galactosidase (see ESI†).

**Table 1:** IC_50_ values (nM) of paclitaxel and rotaxane **1** on KB, H661 and MDA-MB-231 cell lines after two days treatment

	KB	H661	MDA-MB-231
Paclitaxel	19.4 ± 3.1	37.3 ± 8.0	41.2 ± 5.0
**1**	88.7 ± 10.3	144.0 ± 3.0	192.3 ± 19.1

**Fig. 5:**
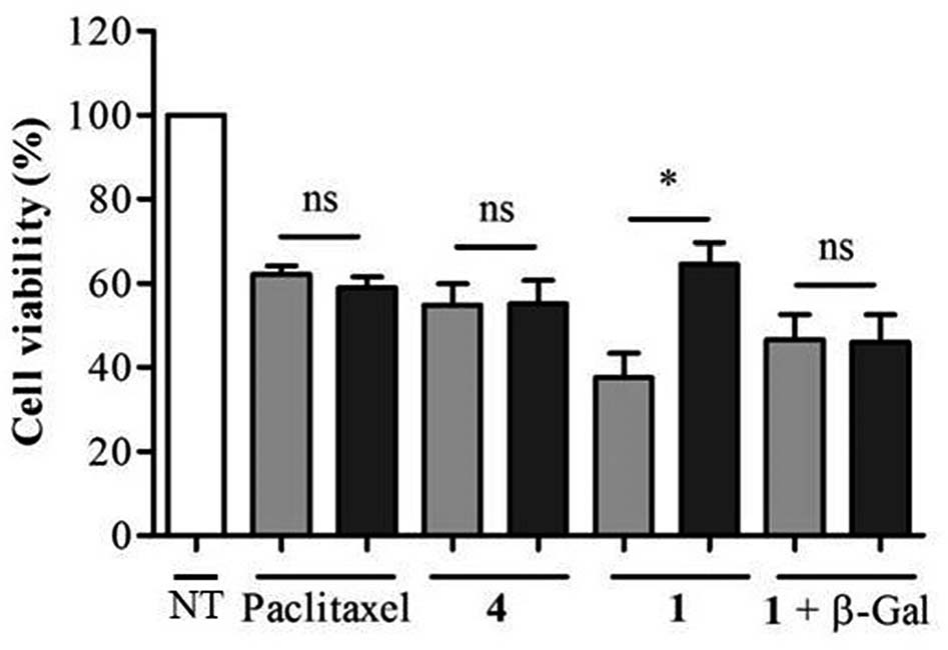
Viability of KB tumor cells treated for 48 h with the indicated compounds. White bar: untreated KB cells (NT). Light grey bar: non-transfected KB cells. Dark grey bar: siRNA-transfected KB cells. Rotaxane **1** and thread **4** were tested at 100 nM. Paclitaxel was tested at 20 nM.

As shown in Fig. 5, when incubated at 100 nM the enzyme-responsive rotaxane **1** was approximately 2-fold more toxic for non-transfected compared to transfected KB cells (with 65% and 35% reduction of cell viability respectively). In contrast, no significant difference of cytotoxicity was observed with either paclitaxel or thread **4** on both types of cells. Furthermore, when placed in the presence of *E. coli* β-galactosidase, rotaxane **1** exhibited the same level of toxicity for non-transfected and transfected KB cells. Overall, these results confirmed that the cytotoxicity of **1** is dependent of the intracellular activity of β-galactosidase.

Finally, we evaluated the antiproliferative activity of the interlocked system **1** on HUVEC normal cells. Compared to cancer cells tested in this study, rotaxane **1** was less toxic for HUVEC cells with only a 20% reduction of cell viability at the highest tested dose of 500 nM (see ESI†).

The selective killing of tumor cells represents one of the main challenges of cancer chemotherapy. As a drug delivery system based on **1** should exhibit higher toxicity for cancer cells expressing a high concentration of the enzyme compared to healthy cells.

## Conclusion

In summary, we developed a functional interlocked molecular system programmed to release a potent anticancer drug within cancer cells in response to a determined sequence of two distinct enzymatic activations. The [2]-rotaxane includes a novel β-galactosidase-responsive self-opening macrocycle that enables both the efficient protection and release of an anticancer prodrug that is, in turn, activated by intracellular esterases. Biological evaluations demonstrated that the molecular system exhibits an appreciable level of discrimination for cancer cells overexpressing β-galactosidase, whereas the majority of current anticancer drugs lack intrinsic selectivity. The sequence of events leading to the release of the active form of the drug occurs under conditions as complex as those prevailing within living cells, suggesting that this process could operate in biochemical environments. Further studies are underway to evaluate the potential of rotaxane-based architectures as potential anticancer prodrugs.

## Author contributions

S.P. conceived the project and wrote the paper. R.B., T.L. and I.T. synthesised the compounds. R.B and I.T. undertook enzymatic evaluations and wrote ESI. E.P. and J.C. designed and performed biological evaluations. P.P conducted HPLC/HRMS experiments. R.B. and T.L. prepared early versions of the enzyme-sensitive rotaxane. A.E.F designed early versions of the self-opening macrocycle. R.B., T.L., I.T. B.R. and A.E.F. analysed the data. D.A.L. and V.A. discussed the results and commented on the manuscript at all stages.

## References

[cit1] van Dongen S. F. M., Cantekin S., Elemans J., Rowan A. E., Nolte R. J. M. (2014). Chem. Soc. Rev..

[cit2] Kay E. R., Leigh D. A., Zerbetto F. (2007). Angew. Chem., Int. Ed..

[cit3] Smithrud D. B., Wang X., Tarapore P., Ho S. (2013). ACS Med. Chem. Lett..

[cit4] Baumes J. M., Gassensmith J. J., Giblin J., Lee J. J., White A. G., Culligan W. J., Leevy W. M., Kuno M., Smith B. D. (2010). Nat. Chem..

[cit5] Coti K. K., Belowich M. E., Liong M., Ambrogio M. W., Lau Y. A., Khatib H. A., Zink J. I., Khashab N. M., Stoddart J. F. (2009). Nanoscale.

[cit6] Ambrogio M. W., Thomas C. R., Zhao Y.-L., Zink J. I., Stoddart J. F. (2011). Acc. Chem. Res..

[cit7] Meng H., Xue M., Xia T., Zhao Y.-L., Tamanoi F., Stoddart J. F., Zink J. I., Nel A. E. (2010). J. Am. Chem. Soc..

[cit8] Moon C., Kwon Y. M., Lee W. K., Park Y. J., Yang V. C. (2007). J. Control. Release.

[cit9] Fernandes A., Viterisi A., Coutrot F., Potok S., Leigh D. A., Aucagne V., Papot S. (2009). Angew. Chem., Int. Ed..

[cit10] Aucagne V., Hänni K. D., Leigh D. A., Lusby P. J., Walker D. B. (2006). J. Am. Chem. Soc..

[cit11] Aucagne V., Berna J., Crowley J. D., Goldup S. M., Hänni K. D., Leigh D. A., Lusby P., Ronaldson V. E., Slawin A. M., Viterisi A., Walker D. B. (2007). J. Am. Chem. Soc..

[cit12] Beves J. E., Blight B. A., Campbell C. J., Leigh D. A., McBurney R. T. (2011). Angew. Chem., Int. Ed..

[cit13] Mekhail T. M., Markman M. (2002). Expert Opin. Pharmacother..

[cit14] Skwarczynski M., Hayashi Y., Kiso Y. (2006). J. Med. Chem..

[cit15] Kingston D. G. I. (2000). J. Nat. Prod..

[cit16] Damen E. W. P., Wiegerinck P. H. G., Braamer L., Sperling D., de Vos D., Scheeren H. W. (2000). Bioorg. Med. Chem..

[cit17] Fernandes A., Viterisi A., Aucagne V., Leigh D. A., Papot S. (2012). Chem. Commun..

[cit18] Bosmann H. B., Hall T. C. (1974). Proc. Natl. Acad. Sci. U. S. A..

[cit19] Paradis V., Youssef N., Dargère D., Bâ N., Bonvoust F., Descharette J., Bedossa P. (2001). Hum. Pathol..

[cit20] te Poele R. H., Okorokov A. L., Jardine L., Cummings J., Joel S. P. (2002). Cancer Res..

[cit21] Papot S., Tranoy I., Tillequin F., Florent J.-C., Gesson J.-P. (2002). Curr. Med. Chem.: Anti-Cancer Agents.

[cit22] Tranoy-Opalinski I., Fernandes A., Thomas M., Gesson J.-P., Papot S. (2008). Anti-Cancer Agents Med. Chem..

[cit23] The opening of the macrocycle can also occur *via* a 1,4-elimination process leading to the formation of the corresponding azaquinone methide that is the region-isomer of the intermediate **5**

