# Supplementary material for: A mechanically interlocked molecular system programmed for the delivery of an anticancer drug
Source: Chem Sci. 2015 Feb 25;6(4):2608–13. doi: 10.1039/c5sc00648a (PMC5649224; doi:10.1039/c5sc00648a)
Supplement: Supplementary file 1 [file SC-006-C5SC00648A-s001.pdf]

Electronic Supplementary Material (ESI) for Chemical Science.
This journal is © The Royal Society of Chemistry 2015



# Supporting Information

## A Mechanically Interlocked Molecular System Programmed for the Delivery of an Anticancer Drug.

Romain Barat,[a] Thibaut Legigan,[a] Isabelle Tranoy-Opalinski,[a] Brigitte Renoux,[a] Elodie Péraudeau,[b, c] Jonathan Clarhaut,[a, c] Pauline Poinot,[d] Antony E. Fernandes,[e] Vincent Aucagne,[f] David A. Leigh[g] and Sébastien Papot.[a]

[a] *Université de Poitiers, UMR-CNRS 7285, Institut de Chimie des Milieux et des Matériaux de Poitiers, Equipe Synthèse, 4 rue Michel Brunet, TSA 51106, 86073 Poitiers, France.*

[b] *CHU de Poitiers, 2 rue de la Milétrie, CS 90577, 86021 Poitiers, France.*

[c] *Université de Poitiers, CNRS ERL 7368, 1 rue Georges Bonnet, TSA 51106, 86073 Poitiers, France.*

[d] *Université de Poitiers, UMR-CNRS 7285, Institut de Chimie des Milieux et des Matériaux de Poitiers, Equipe Eau, Geochimie Organique, Santé (EGS), 4 rue Michel Brunet, TSA 51106, 86073 Poitiers, France.*

[e] *Institute of Condensed Matter and Nanoscience, Université Catholique de Louvain, place Croix du Sud, 1348 Louvain-la-Neuve, Belgium.*

[f] *Centre de Biophysique Moleculaire, CNRS, rue Charles Sadron, 45071 Orléans Cedex 2, France*

[g] *Scool of chemistry University of Manchester, Oxford road, Manchester MP13 9PL, UK*

**E-mail: sebastien.papot@univ-poitiers.fr*

## Table of Contents


**I. Chemistry Section** S3

**I.1.General experimental methods** S3

**I.2. Synthesis of compounds 6, 7, 8, 9 and 1** S5

**I.3. Synthetic procedures and characterization details with $^{1}$H NMR and $^{13}$C NMR plots** S9

**I.4. β-galactosidase cleavage** S48

**I.5. Stability toward rat plasma esterases** S50

**II. Biological Section** S52

**II.1. Cell culture** S52

**II.2. Cellular viability** S52

**II.3. Immunofluorescence** S54

**II.4. Small Interfering RNA Transfection** S55

**III. References** S57

# I. Chemistry Section

## I.1. General experimental methods

All reactions were performed under a nitrogen atmosphere. Unless otherwise stated, solvents used were of HPLC quality. Chemicals were of analytical grade from commercial sources and were used without further purification. Reaction were monitored using precoated silica gel TLC plates MACHEREY-NAGEL ALUGRAM® SIL G/$UV_{254}$. (0.2 mm silica gel 60). Spots were visualized under 254 nm UV light and/or by dipping the TLC plate into a solution of phosphomolybdic acid (3 g) in ethanol (100 mL) followed by heating with a heat gun. Flash column chromatographies were performed using MERK silica gel 60 (15-40 $\mu$m) as the stationary phase. Automatic chromatographies were performed with a REVELERIS® iES instrument equipped with UV and ELSD detectors and using flash cartridges Resolv® or Reveleris® silica 40 $\mu$m. $^{1}$H and $^{13}$C NMR spectra were respectively recorded at 400 MHz and 100 MHz on a Bruker 400 Avance III instrument, equipped with an ultra shielded magnet and a BBFO 5 mm broadband probe. $^{1}$H and $^{13}$C NMR of compounds **4**, **6**, **1** were recorded at 500 MHz and 125 MHz respectively on a Bruker spectrometer equipped with a cryoprobe TXI 1H-13C-15N (5mm) in the Prism platform at University of Rennes. Chemical shifts (δ) are reported in parts per million (ppm) from high to low field and referenced to residual solvent peaks. Coupling constants (*J*) are reported in hertz (Hz). Standard abbreviations indicating multiplicity are used as follows: br = broad, s = singlet, d = doublet, t = triplet, q = quartet, qi = quintet, m = multiplet, dd = doublet of doublets, etc. Melting points were measured on a Büchi Melting Point B-545 instrument and are uncorrected. Accurate mass was determined for all derivatives through their infusion on high resolution ESI mass spectrometers in the CRMPO, at the University of Rennes 1, in the Organic Analysis Center IC2MP at University of Poitiers and in the ICOA at the University of Orléans. Analytical RP-

HPLC were carried out on a Dionex Ultimate 3000 system equipped with a UV/Visible variable wavelength detector and with a reverse-phase Acclaim® column (120, C18, 250x4.6 mm, 5 $\mu$m, 120 Å) at 30°C and 1 mL.min$^{-1}$. Semi-preparative RP-HPLC were performed with a VWR LaPrep system equipped with a spectrophotometer LaPrep P314, a preparative pump LaPrep P110 and a semi-preparative column ACE® C18-AR (100x10 mm, 5 $\mu$m) at room temperature and a flow rate of 4 mL.min$^{-1}$. All chromatograms were recorded at 254 nm. LC/MS experiments were performed on an Accela UHPLC system coupled to a hybrid high resolution mass spectrometer Q-Exactive. An Acclaim® C18 column (250x4.6 mm, 5 $\mu$m, 120 Å) at 30°C was used for chromatographic separation after injection of 20 µL of sample at a flow rate of 0.5 mL.min$^{-1}$. The column effluent first passed through an Accela PDA detector and introduced into the electrospray ionisation source (ESI) of the mass spectrometer. Analyses were performed in the positive ion mode. The electrospray voltage was set at 4.0 kV. The capillary and heater temperatures were 275°C and 300°C respectively. The sheath, sweep and auxiliary gas (nitrogen) flow rates were set at 35, 10 and 20 (arbitrary units). Analysis of data was performed with Xcalibur software.

## I.2. Synthesis of compounds 6, 7, 8, 9 and 1.

Compound **6** was prepared according to the following strategy:

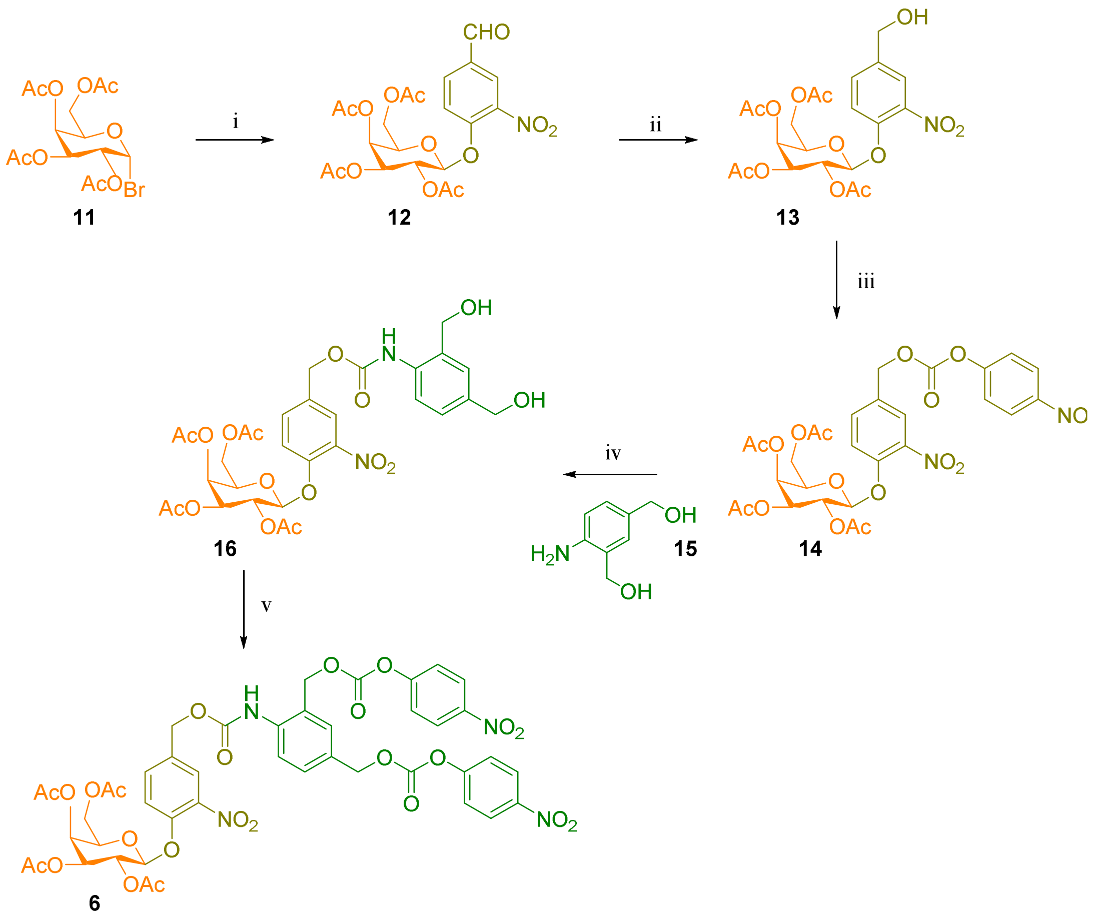


**Scheme S1.** *Reagents and conditions:* (i) 4-Hydroxy-3-nitrobenzaldehyde, $Ag_2CO_3$, $CH_3CN$, RT, 4 h, 89%; (ii) $NaBH_4$, THF/MeOH 1/1, 0°C, 30 min. 84%; (iii) 4-nitrophenyl chloroformate, pyridine, $CH_2Cl_2$, 0°C to RT, 30 min., 86%; (iv) **15**, HOBt, DMF, 50°C, 18 h, 91%; (v) 4-nitrophenyl chloroformate, pyridine, $CH_2Cl_2$, 0°C to RT, 1 h, 89%;

Compound **7** was prepared according to the following strategy:

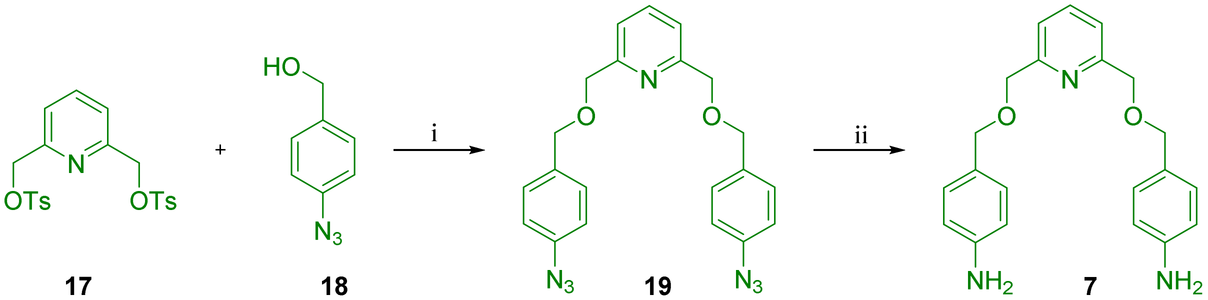


**17** **18** **19** **7**

**Scheme S2.** *Reagents and conditions:* (i) NaH, DMF, RT, 45 min., 96%; (ii) $Me_2PhP$, $NH_4OH$, THF, RT, 1 h, 97%.

Compound **8** was prepared according to the following strategy:

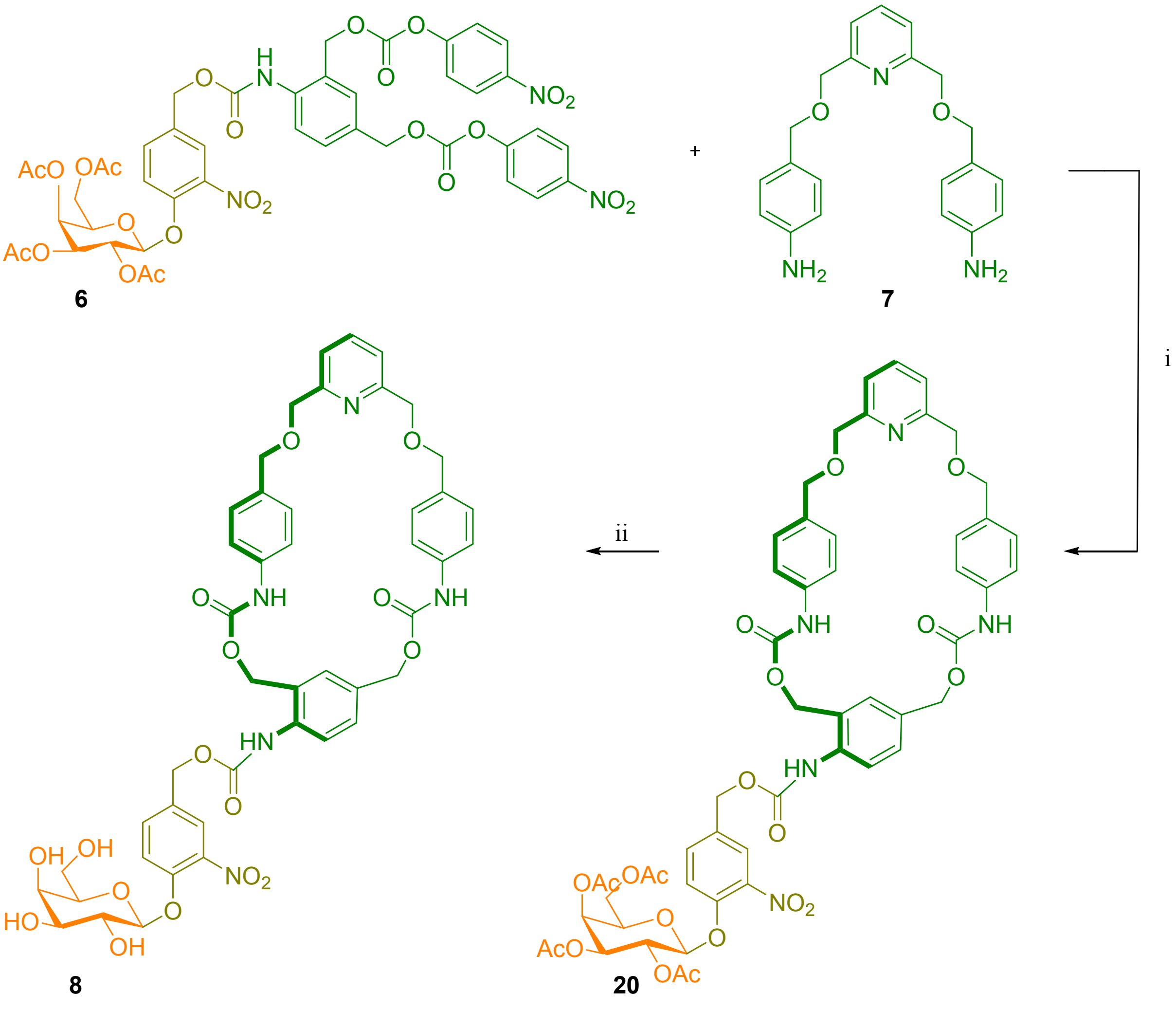


**Scheme S3.** *Reagents and conditions:* (i) a) HOBt, DMF [0.08M], RT, 5 h; b) DMF [0.001M], 30°C, 96 h; (ii) MeONa, MeOH/$CH_2Cl_2$ 2:1, 0°C, 1 h 15, 28% (over two steps).

Compound **9** was prepared according to the following strategy:

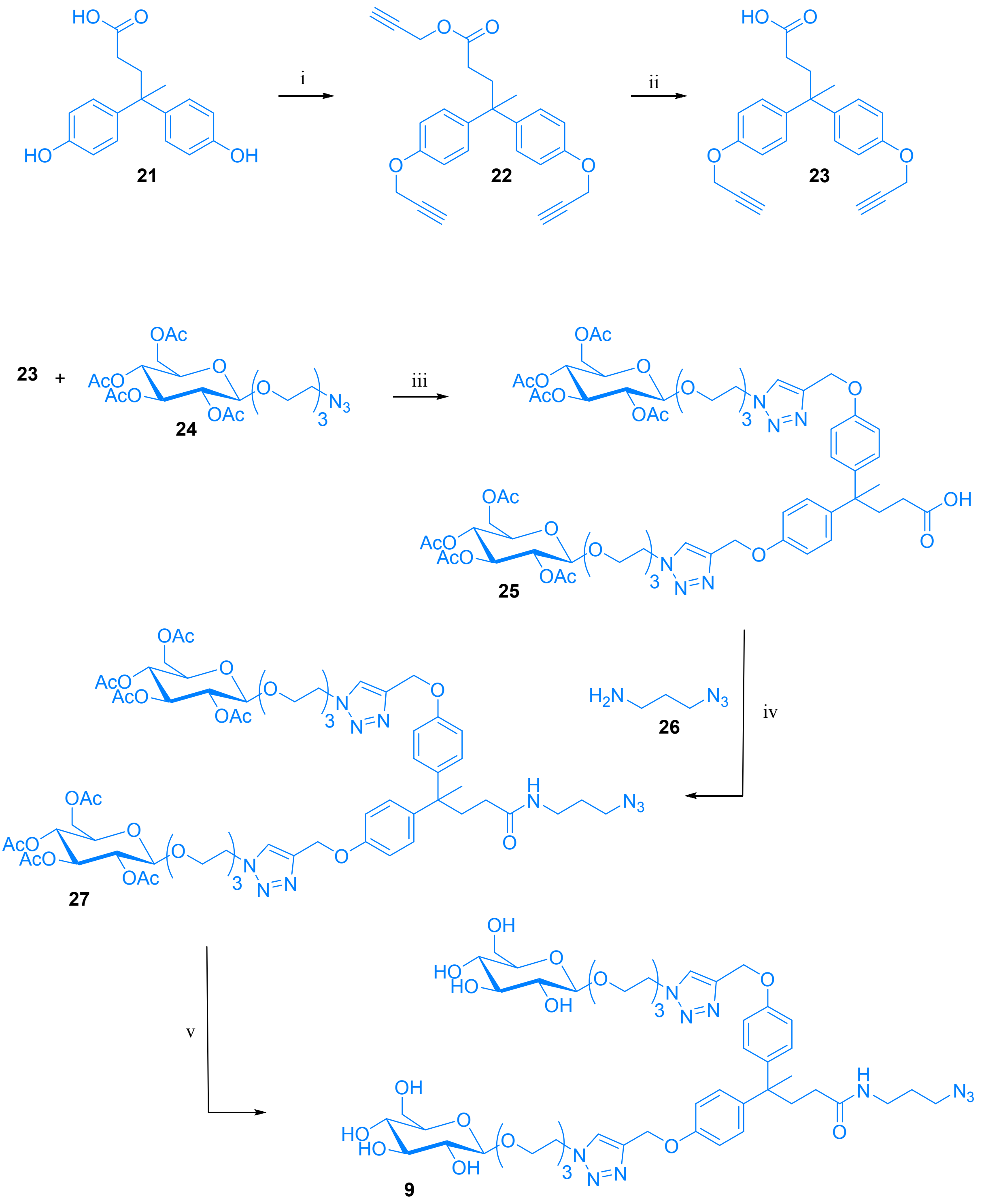


**Scheme S4.** *Reagents and conditions:* (i) $K_2CO_3$, propargyl bromide, DMF, 50°C, 22 h, 80%; (ii) NaOH (2M)/MeOH 3/2, RT, 18 h, 94%; (iii) $[Cu(CH_3CN)_4](PF_6)$, $CH_2Cl_2$, RT, 48 h, 78%; (iv) **26**, EDC, DMAP, DMF, RT, 24 h, 59%; (v) MeONa/MeOH, RT, 18 h, 100%.

Compound **1** was prepared according to the following strategy:

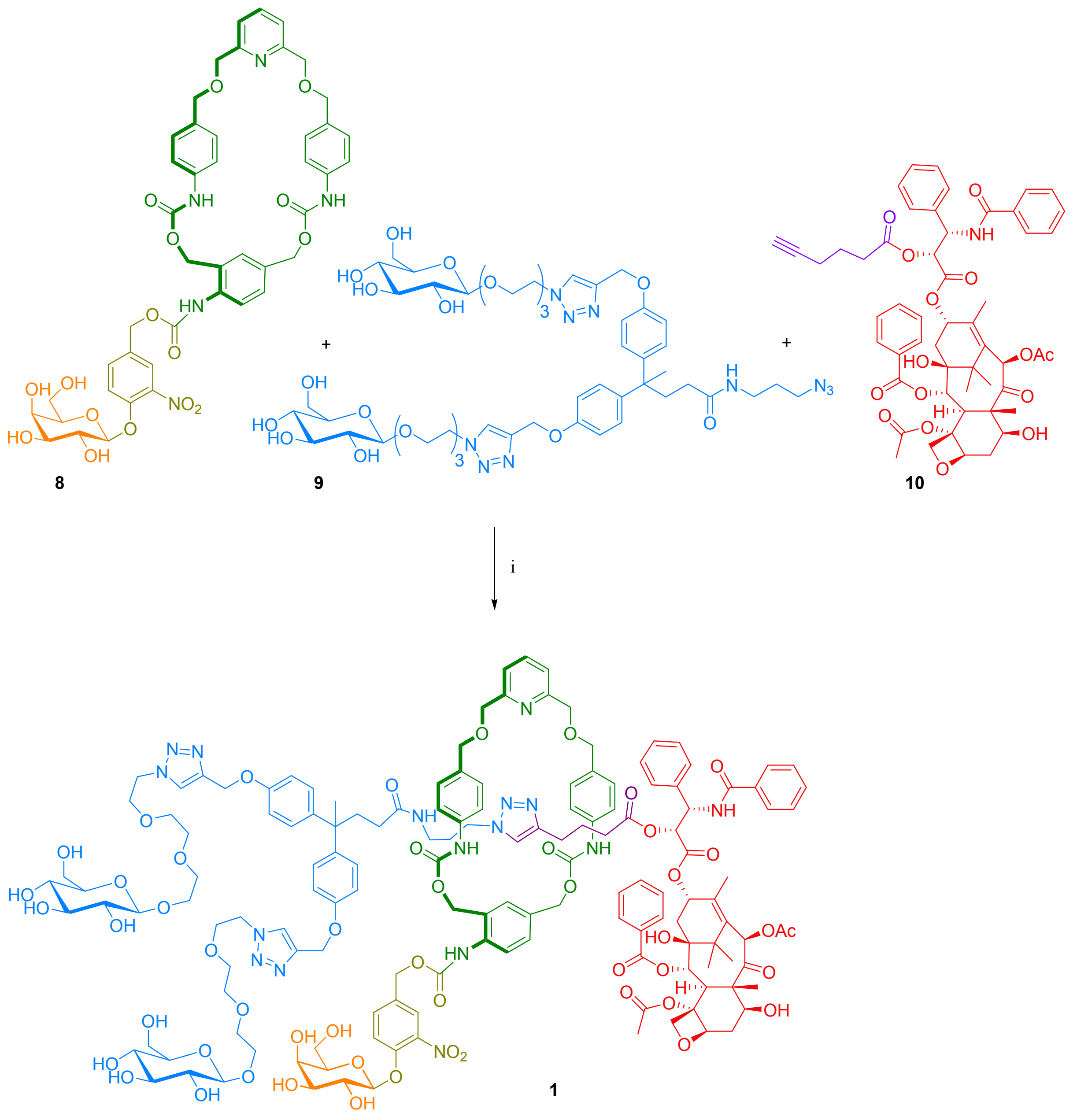


**Scheme S5.** *Reagents and conditions:* $[Cu(CH_3CN)_4](PF_6)$, $CH_2Cl_2$/MeOH 4/1, RT, 45 h, 52%.

## I.3. Synthetic procedures and characterization details with $^{1}$H NMR and $^{13}$C NMR plots

### *Preparation of compound 12*

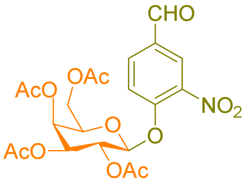


**12**

To a stirred solution of 2,3,4,6-tetra-*O*-acetyl-alpha-D-galactopyranosyl bromide **11** (2 g, 4.86 mmol) in $CH_3CN$ (32 mL) cooled at 0°C, were added $Ag_2CO_3$ (2.67 g, 9.72 mmol, 2 equiv.) and a solution of 4-hydroxy-3-nitrobenzaldehyde (861 mg, 5.34 mmol, 1.1 equiv.) in $CH_3CN$ (10 mL). Stirring was continued for 4 hours at room temperature and the solution was filtered through a pad of celite and concentrated under reduced pressure. Purification by column chromatography over silica gel (gradient elution 30% to 50% ethyl acetate in petroleum ether) gave galactoside **12** (2.16 g, 4.3 mmol, 89%) as a white foam.

**R$_f$:** 0.5 (petroleum ether:ethyl acetate 1:1).

**$^{1}$H NMR** (400 MHz, $CDCl_3$): δ 2.02-2.06-2.13-2.19 (4s, 12H), 4.24 (m, 3H), 5.14 (dd, $J$ = 10.0, 3.2, 1H), 5.21 (d, $J$ = 7.9, 1H), 5.49 (d, $J$ = 3.2, 1H), 5.58 (dd, $J$ = 10.0, 7.9, 1H), 7.48 (d, $J$ = 8.6, 1H), 8.06 (dd, $J$ = 8.6, 2.0, 1H), 8.31 (d, $J$ = 2.0, 1H), 9.98 (s, 1H).

**$^{13}$C NMR** (100 MHz, $CDCl_3$): δ 20.7 (x3), 20.8, 61.5, 66.7, 67.7, 70.5, 71.9, 100.1, 118.9, 126.9, 131.6, 134.1, 141.3, 153.6, 169.3, 170.2, 170.4 (x2), 188.7.

**HRESI-MS**: m/z 520.1066 (calcd. for $C_{21}H_{23}NO_{13}Na$ 520.1067 $[M+Na]^+$).

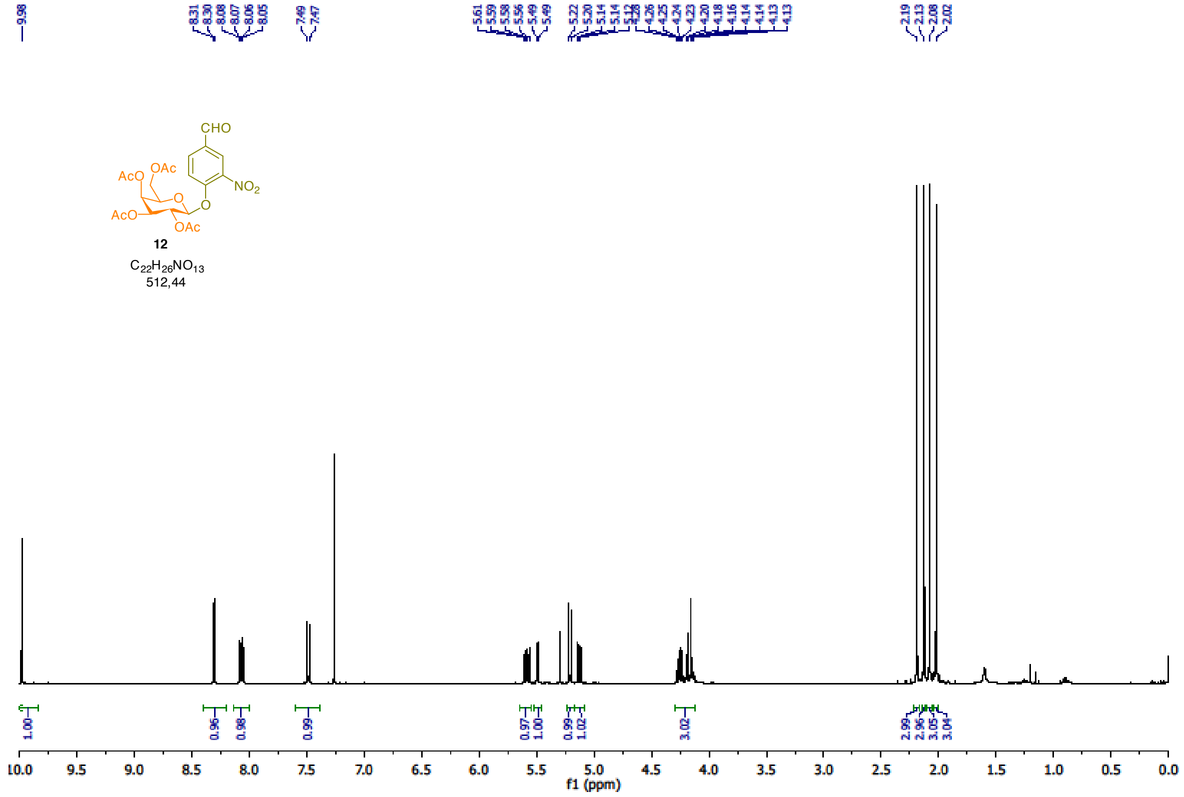


$^1$H NMR spectrum (400 MHz, 298 K, $CDCl_3$) of **12**.

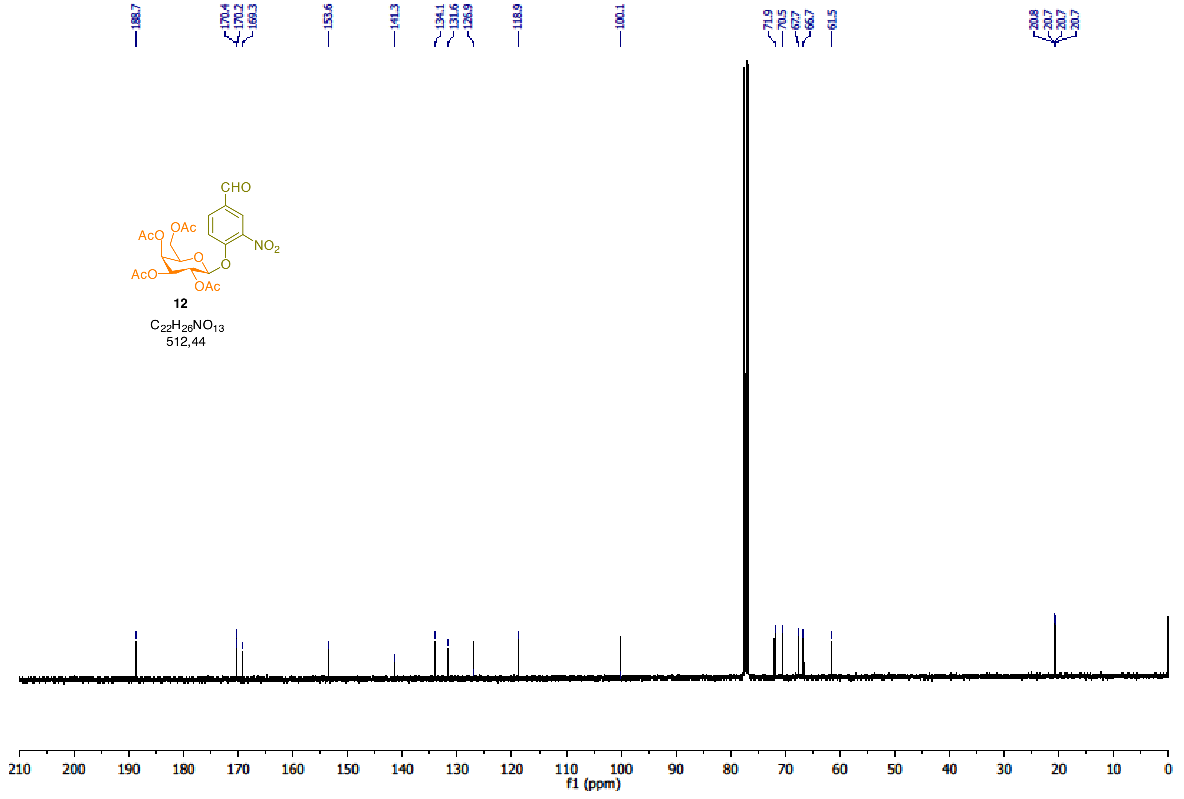


$^{13}$C NMR spectrum (100 MHz, 298 K, $CDCl_3$) of **12**.

***Preparation of compound 13***

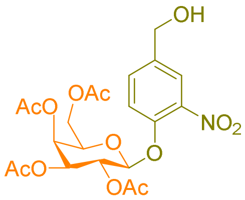

**13**

To a stirred solution of aldehyde **12** (2 g, 4 mmol) in THF:MeOH 1:1 (30 mL) cooled at 0°C, was added portion wise $NaBH_4$ (177 mg, 2 mmol, 0.5 equiv.) and stirring was continued for 30 minutes at 0°C. The reaction was hydrolyzed with HCl 0.1N and extracted with $CH_2Cl_2$ (3x). The combined organic layers were dried over $MgSO_4$, filtered and concentrated under reduced pressure. The resulting crude material was purified by column chromatography over silica gel (petroleum ether:ethyl acetate 25:75) to give benzylic alcohol **13** (1.67 g, 3.36 mmol, 84%) as a white solid.

**$R_f$:** 0.3 (petroleum ether/ethyl acetate 25:75).

**m.p.:** 193°C.

**$^1$H NMR** (400 MHz, $CDCl_3$): δ 2.02-2.07-2.13-2.19 (4s. 12H), 4.17 (m, 3H), 4.73 (d, *J* = 5.6, 2H), 5.06 (d, *J* = 7.9, 1H), 5.10 (dd, *J* = 10.0, 3.3, 1H), 5.47 (d, *J* = 3.3, 1H), 5.55 (dd, *J* = 10.0, 7.9, 1H), 7.35 (d, *J* = 8.6, 1H), 7.52 (dd, *J* = 8.6, 2.0, 1H), 7.80 (d, *J* = 2.0, 1H).

**$^{13}$C NMR** (100 MHz, $CDCl_3$): δ 20.7 (x4), 61.5, 63.5, 66.8, 67.9, 70.7, 71.5, 100.9, 120.0, 123.3, 131.9, 137.3, 141.4, 148.5, 169.3, 170.3, 170.4, 170.5.

**HRESI-MS**: m/z 522.1224 (calcd. for $C_{21}H_{25}NO_{13}Na$ 522.1224 $[M+Na]^+$).

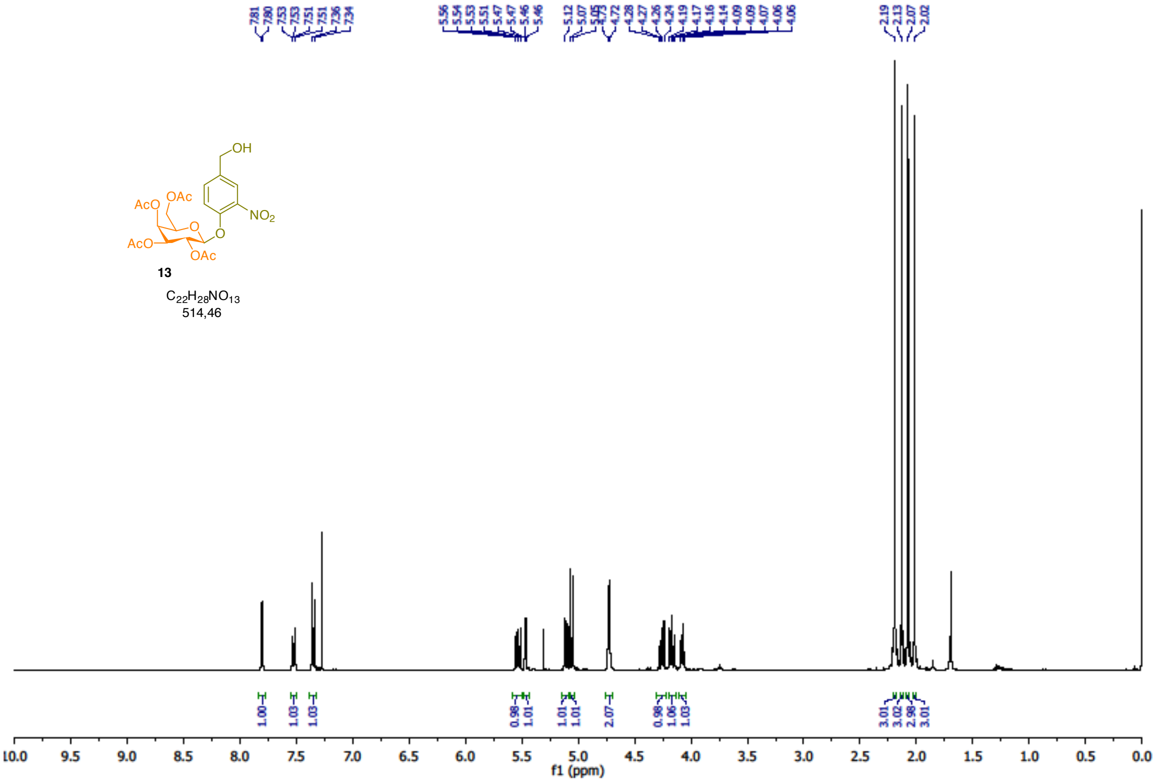

$^{1}$H NMR spectrum (400 MHz, 298 K, $CDCl_3$) of **13**.

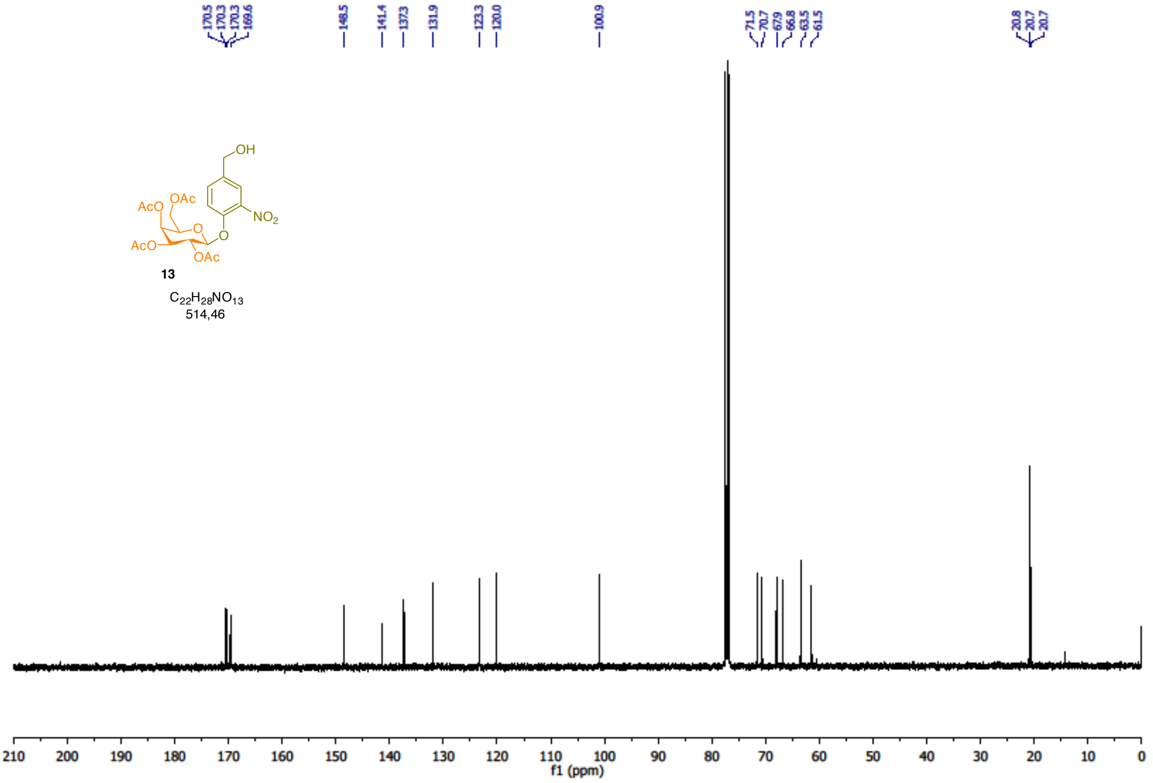

$^{13}$C NMR spectrum (100 MHz, 298 K, $CDCl_3$) of **13**.

***Preparation of compound 14***

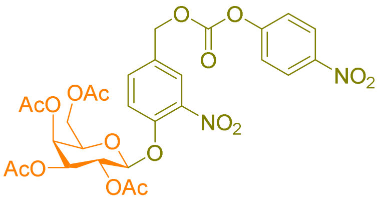

**14**

To a stirred solution of alcohol **13** (1.47 g, 2.94 mmol) in $CH_2Cl_2$ (15 mL) cooled at 0°C, were added successively 4-nitrophenyl chloroformate (1.78 g, 8.83 mmol, 3 equiv.) and pyridine (714 $\mu$L, 8.83 mmol, 3 equiv.) and stirring was continued for 30 minutes at room temperature. The reaction was hydrolyzed with a saturated solution of $NaHCO_3$ and the aqueous phase was extracted with $CH_2Cl_2$ (3x). The combined organic layers were dried over $MgSO_4$, filtered and concentrated under reduced pressure. The resulting crude material was purified by column chromatography over silica gel (gradient elution 30% to 50% ethyl acetate in petroleum ether) to give carbonate **14** (1.68 g, 2.52 mmol, 86%) as a white foam.

**R*f***: 0.2 (petroleum ether:ethyl acetate 1:1).

**$^1$H NMR** (400 MHz, $CDCl_3$): δ 2.02-2.08-2.13-2.19 (4s, 12H), 4.20 (m. 3H), 5.13 (dd, *J* = 10.0, 3.3, 1H), 5.16 (d, *J* = 7.9, 1H,), 5.31 (s, 2H,), 5.50 (d, *J* = 3.3, 1H), 5.56 (dd. *J* = 10.0, 7.9, 1H), 7.39 (d, *J* = 9.2, 2H), 7.42 (d, *J* = 8.6, 1H), 7.65 (dd, *J* = 8.6, 2.0, 1H), 7.92 (d, *J* = 2.0, 1H), 8.26 (d, *J* = 9.2, 2H).

**$^{13}$C NMR** (100 MHz, $CDCl_3$): δ 20.4, 20.5, 20.6 (x2), 61.3, 66.7, 67.7, 68.8, 70.4, 71.4, 100.3, 119.4, 121.7 (x2), 125.2 (x2), 125.3, 130.1, 133.9, 140.9, 145.4, 149.6, 152.2, 155.2, 169.3, 170.0, 170.1, 170.2.

**HRESI-MS**: m/z 687.1287 (calcd. for $C_{28}H_{28}N_2O_{17}Na$ 687.1286 $[M+Na]^+$).

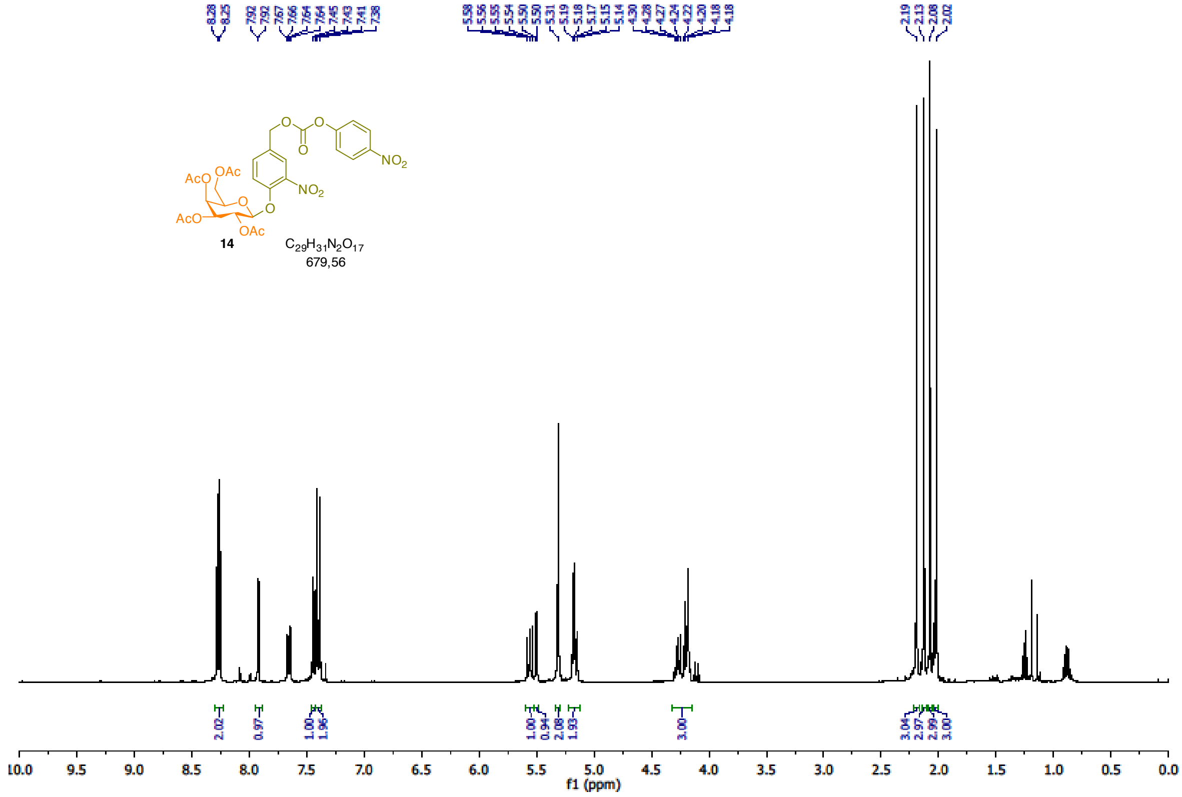

$^{1}$H NMR spectrum (400 MHz, 298 K, $CDCl_3$) of **14**.

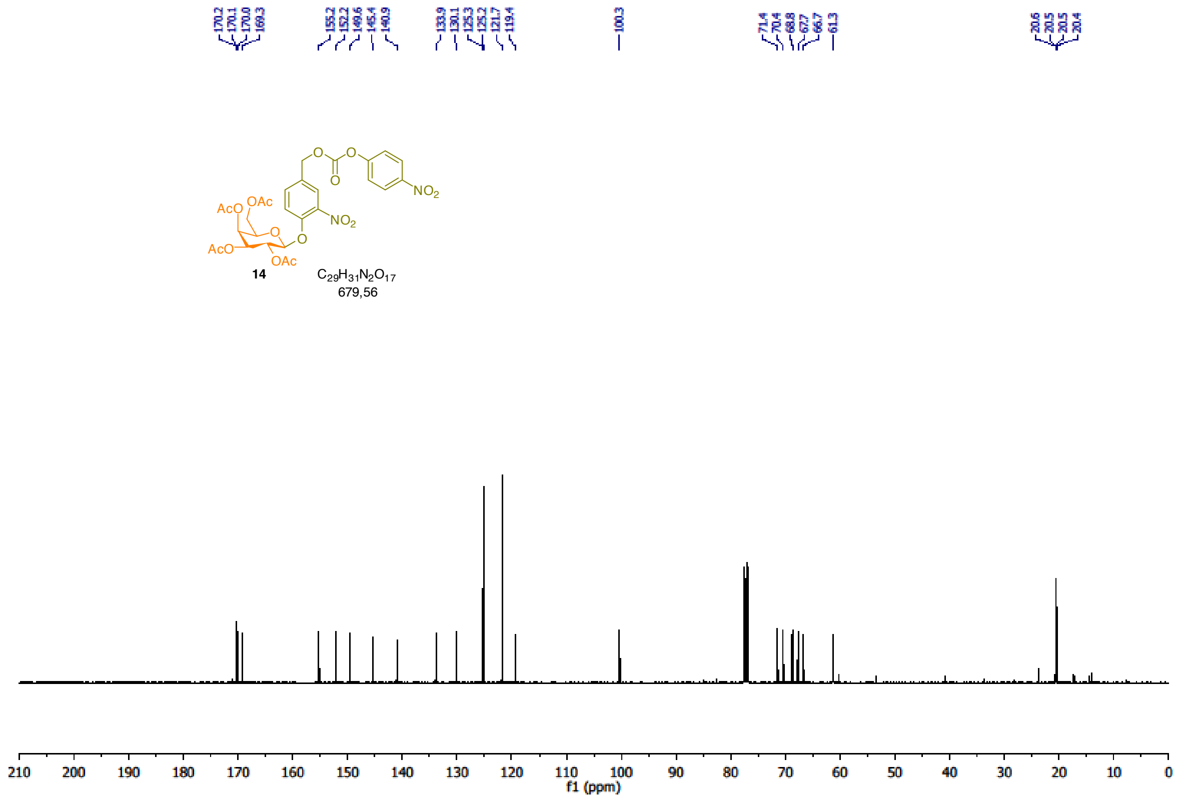

$^{13}$C NMR spectrum (100 MHz, 298 K, $CDCl_3$) of **14**.

### *Preparation of compound 16*

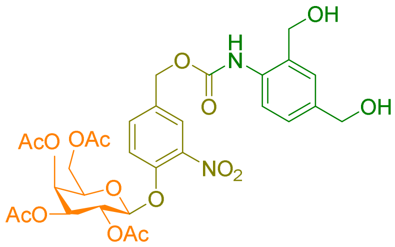


**16**

To a solution of carbonate **14** (365 mg, 0.55 mmol) in DMF (4 mL), were added aniline **15**[1] (169 mg, 1.11 mmol, 2 equiv.) and HOBt (97 mg, 0.71mmol, 1.3 equiv.). The solution was stirred for 18 hours at 50°C. The solvent was removed under reduced pressure and the crude material was purified by column chromatography over silica gel (gradient elution 50% to 75% ethyl acetate in petroleum ether) to give compound **16** (341 mg, 0.5 mmol, 91%) as a white foam.

**R*f*:** 0.4 (ethyl acetate).

**$^{1}$H NMR** (400 MHz, $CDCl_3$): δ 1.99-2.04-2.09-2.14 (4s, 12H), 2.88 (bs, 1H), 3.34 (bs, 1H), 4.20 (m. 3H), 4.50-4.57 (2s, 4H), 5.07 (d, *J* = 8.0, 1H), 5.10 (dd, *J* = 11.0, 3.0, 1H), 5.13 (s, 2H,), 5.43 (d, *J* = 3.0, 1H), 5.50 (dd, *J* = 11.0, 8.0, 1H), 7.06 (d, *J* = 1.3, 1H), 7.17 (dd, *J* = 8.4, 1.7, 1H), 7.33 (d, *J* = 8.6, 1H), 7.54 (dd, *J* = 8.6, 2.0, 1H), 7.77 (d, *J* = 8.4, 1H), 7.82 (d, *J* = 2.0, 1H), 8.15 (bs, 1H).

**$^{13}$C NMR** (100 MHz, $CDCl_3$): δ 20.6, 20.7 (x3), 61.4, 63.9, 64.4, 65.2, 66.9, 67.9, 70.6, 71.5, 100.6, 119.7, 121.0, 125.0, 127.5, 127.6, 129.6, 132.4 (x2), 133.6, 136.5, 141.1, 149.1, 153.6, 169.6, 170.3, 170.4, 170.6.

**HRESI-MS**: m/z 701.1804 (calcd. for $C_{30}H_{34}N_2O_{16}Na$ 701.1801 $[M+Na]^+$).

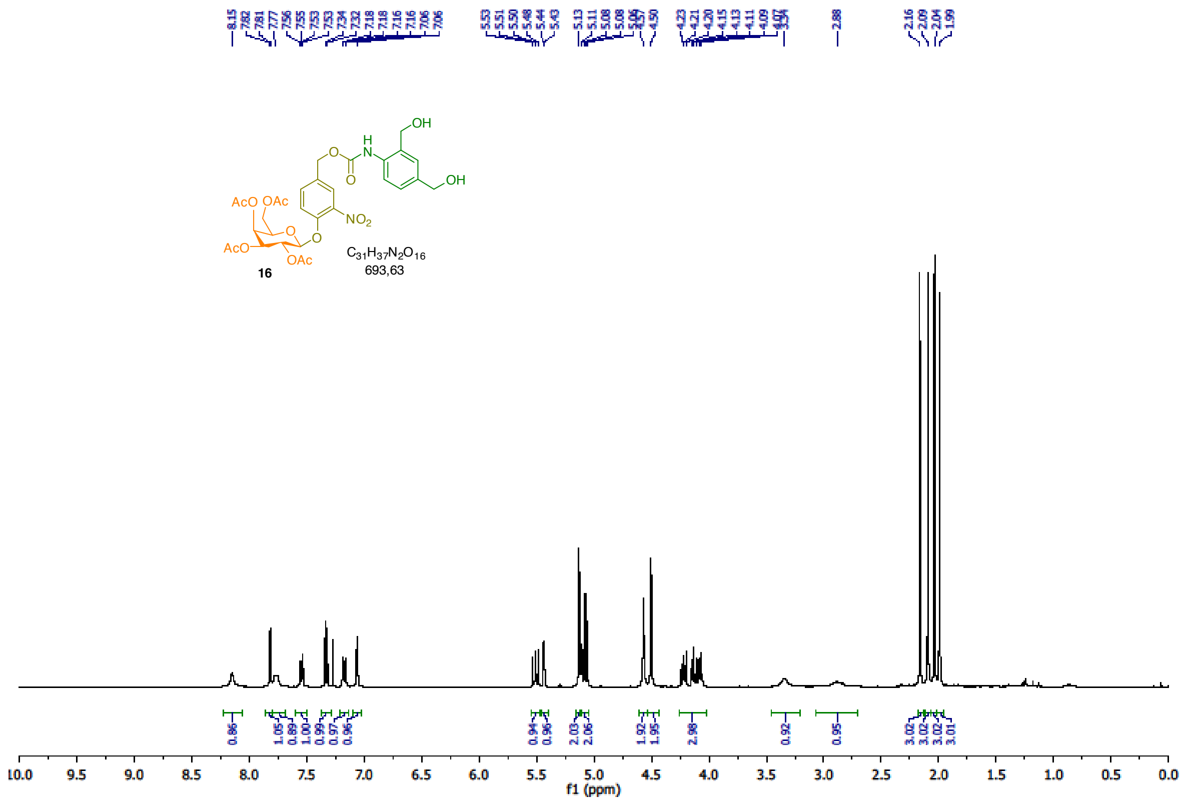

$^{1}H$ NMR spectrum (400 MHz, 298 K, $CDCl_3$) of **16**.

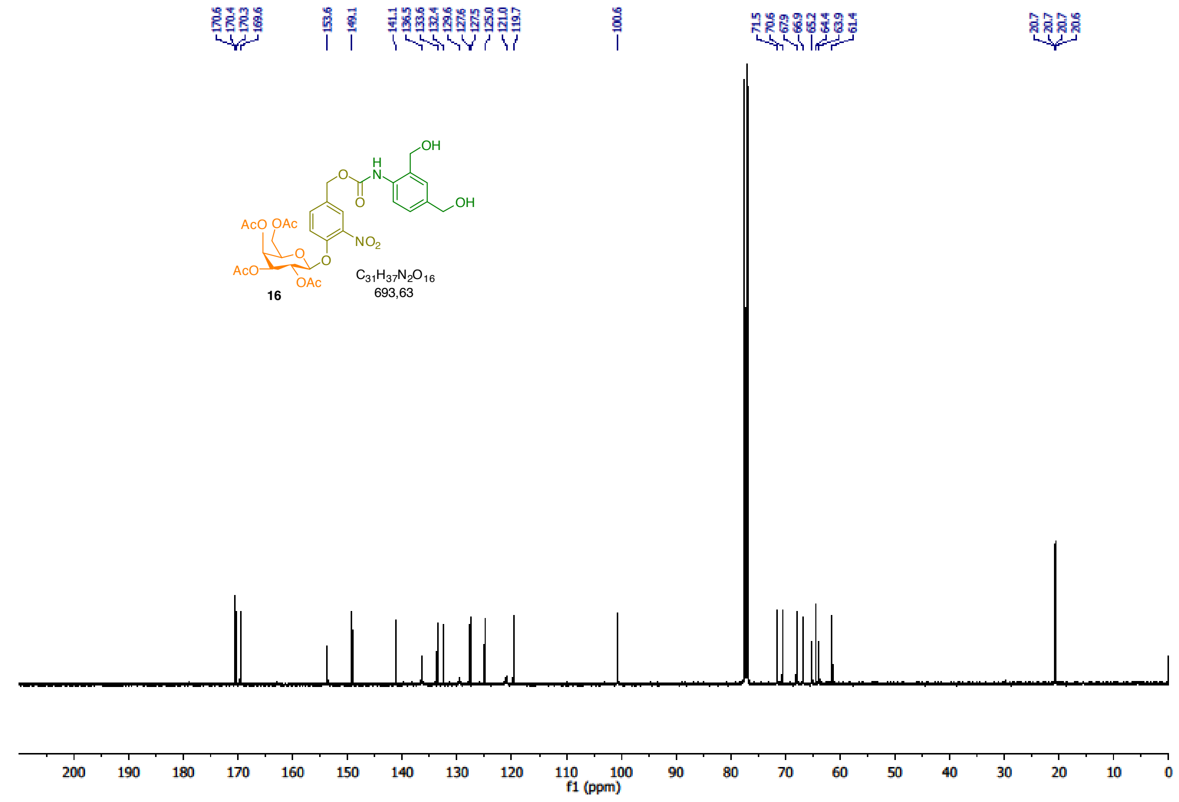

$^{13}C$ NMR spectrum (100 MHz, 298 K, $CDCl_3$) of **16**.

***Preparation of compound 6***

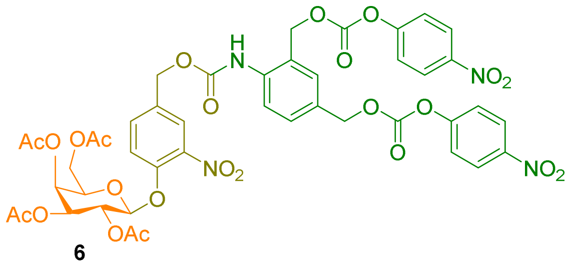

**6**

To a stirred solution of 4-nitrophenyl chloroformate (238 mg, 1.18 mmol, 4 equiv.) and pyridine (96 $\mu$L, 1.18 mmol, 4 equiv.) in $CH_2Cl_2$ (3 mL) cooled at 0°C, was added dropwise a solution of dialcohol **16** (201 mg, 0.29 mmol) in $CH_2Cl_2$ (4 mL) and stirring was continued for 1 hour at room temperature. The reaction was hydrolyzed with a saturated solution of $NaHCO_3$ and the aqueous phase was extracted with $CH_2Cl_2$ (3x). The combined organic layers were dried over $MgSO_4$, filtered and concentrated under reduced pressure. The resulting crude material was purified by column chromatography over silica gel (gradient elution 30% to 50% ethyl acetate in petroleum ether) to give compound **6** (266 mg, 0.26 mmol, 89%) as a white foam.

**R*f*:** 0.6 (petroleum ether:ethyl acetate 6:4).

**$^1$H NMR** (400 MHz, $CDCl_3$): δ 2.02-2.06-2.13-2.19 (4s, 12H), 4.20 (m. 3H), 5.09 (d, *J* = 7.8, 1H), 5.12 (dd, *J* = 10.5, 3.4, 1H), 5.21 (s, 2H), 5.28 (s, 2H), 5.33 (s, 2H), 5.48 (dd, *J* = 3.4, 0.8, 1H), 5.55 (dd, *J* = 10.5, 7.8, 1H), 7.37 (m, 5H), 7.51 (m, 2H), 7.58 (dd, *J* = 8.6, 2.0, 1H), 7.65 (bs, 1H), 7.87 (d, *J* = 2.0, 1H), 7.90 (d, *J* = 7.7, 1H), 8.27 (m, 4H).

**$^{13}$C NMR** (100 MHz, $CDCl_3$): δ 20.6, 20.7, 20.8 (x2), 61.3, 65.5, 66.7, 67.6, 67.8, 70.0, 70.5, 71.5, 100.7, 119.7, 121.8 (x4), 123.3, 125.0, 125.3 (x4), 130.9, 131.1, 131.9, 132.1, 133.6, 137.4, 141.3, 145.5, 145.7, 149.2, 152.4, 152.9, 153.5 (x2), 155.2, 155.5, 169.4, 170.2 (x2), 170.4.

**HRESI-MS**: m/z 1031.1925 (calcd. for $C_{44}H_{40}N_4O_{24}Na$ 1031.1925 $[M+Na]^+$).

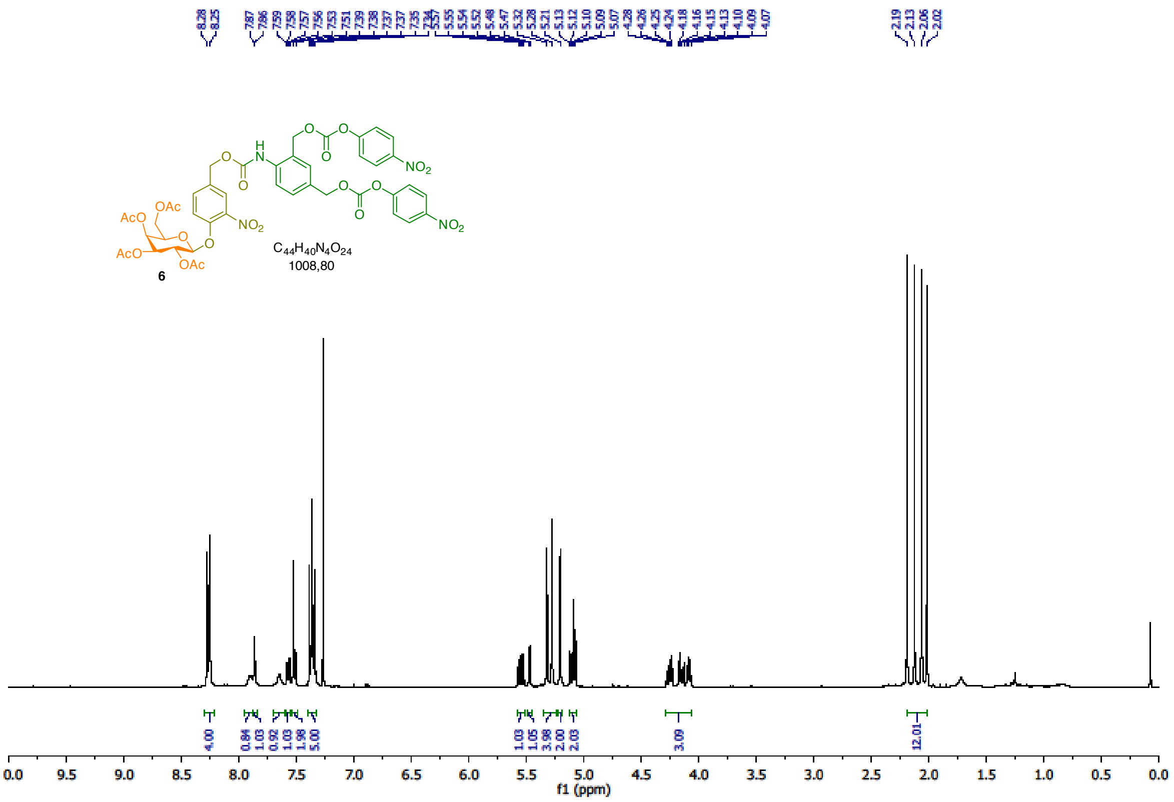

$^{1}H$ NMR spectrum (400 MHz, 298 K, $CDCl_3$) of **6**.

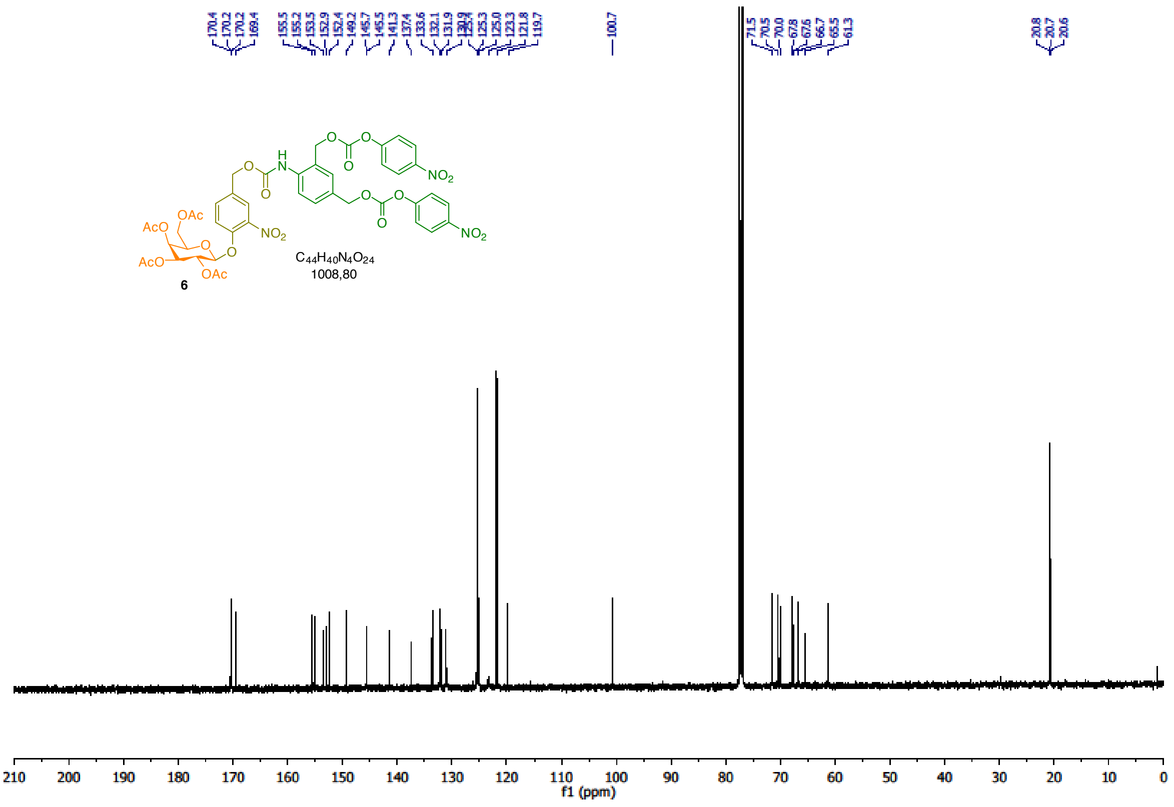

$^{13}C$ NMR spectrum (100 MHz, 298 K, $CDCl_3$) of **6**.

***Preparation of compound 19***

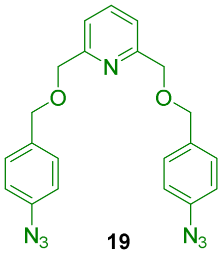

**19**

To a solution of 2,6-[(tosyloxy)methyl]pyridine **17**[2] (3 g, 21.6 mmol) and 4-azidobenzylic alcohol **18**[3] (601 mg, 4.03 mmol, 2.5 equiv.) in anhydrous DMF (29 mL), was added portion wise NaH (60% in mineral oil) (173 mg, 4.03 mmol, 2.5 equiv.) and stirring was continued for 45 minutes at room temperature. The reaction was hydrolyzed with water and the solution was extracted with ethyl acetate (3x). The combined organic layers were washed with a saturated solution of $NaHCO_3$, dried over $MgSO_4$, filtered and concentrated under reduced pressure. The resulting crude material was purified by column chromatography over silica gel (gradient elution 10% to 20% ethyl acetate in petroleum ether) to give **19** (621 mg, 1.55 mmol, 96%) as a yellow solid.

**R*f*:** 0.6 ($CH_2Cl_2$/acetone 9:1).

**m.p.:** 85°C.

**$^1$H NMR** (400 MHz, $CDCl_3$): δ 4.61 (s, 4H), 4.66 (s, 4H), 7.02 (d, *J* = 8.6, 4H), 7.38 (m, 6H), 7.73 (t, *J* = 7.7, 1H).

**$^{13}$C NMR** (100 MHz, $CDCl_3$): δ 72.5 (x2), 73.0 (x2), 119.2 (x4), 120.4 (x2), 129.5 (x4), 134.8 (x2), 138.3, 139.7(x2), 157.8 (x2).

**HRESI-MS**: m/z 424.1496 (calcd. for $C_{21}H_{19}N_7O_2Na$ 424.1498 $[M+Na]^+$).

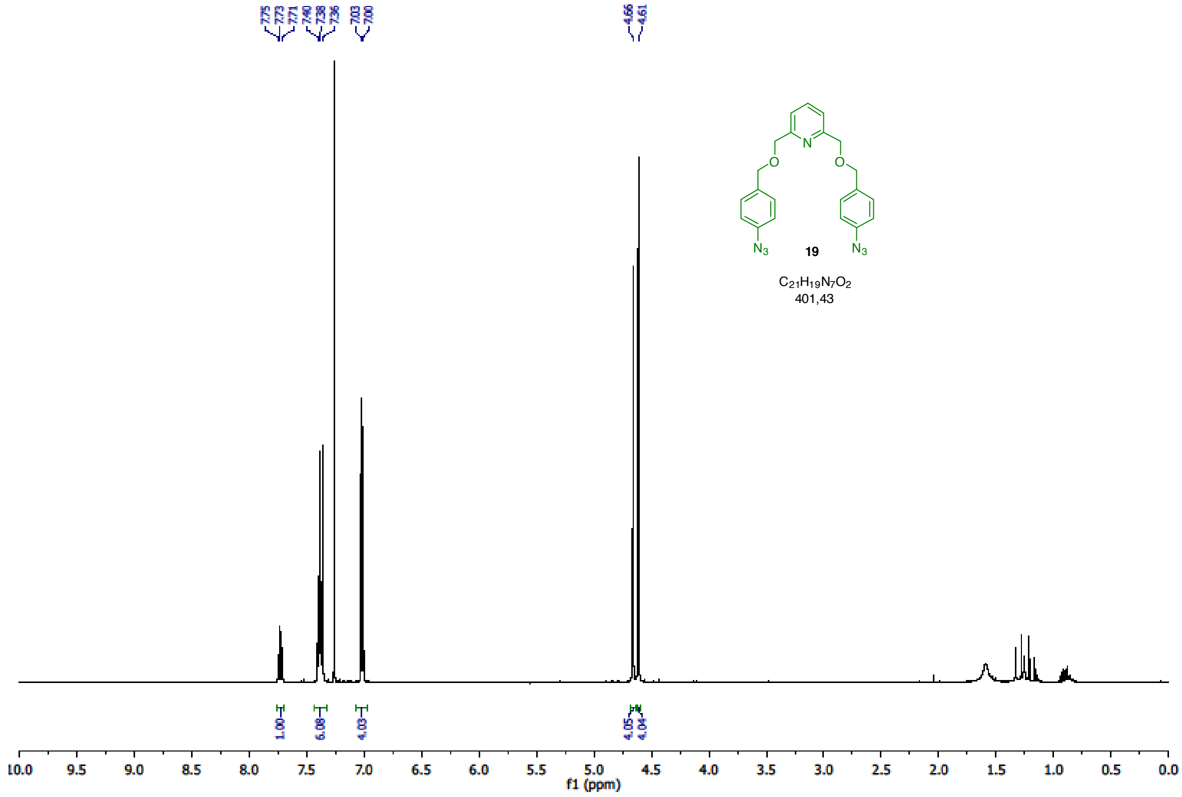


$^{1}$H NMR spectrum (400 MHz, 298 K, $CDCl_3$) of **19**.

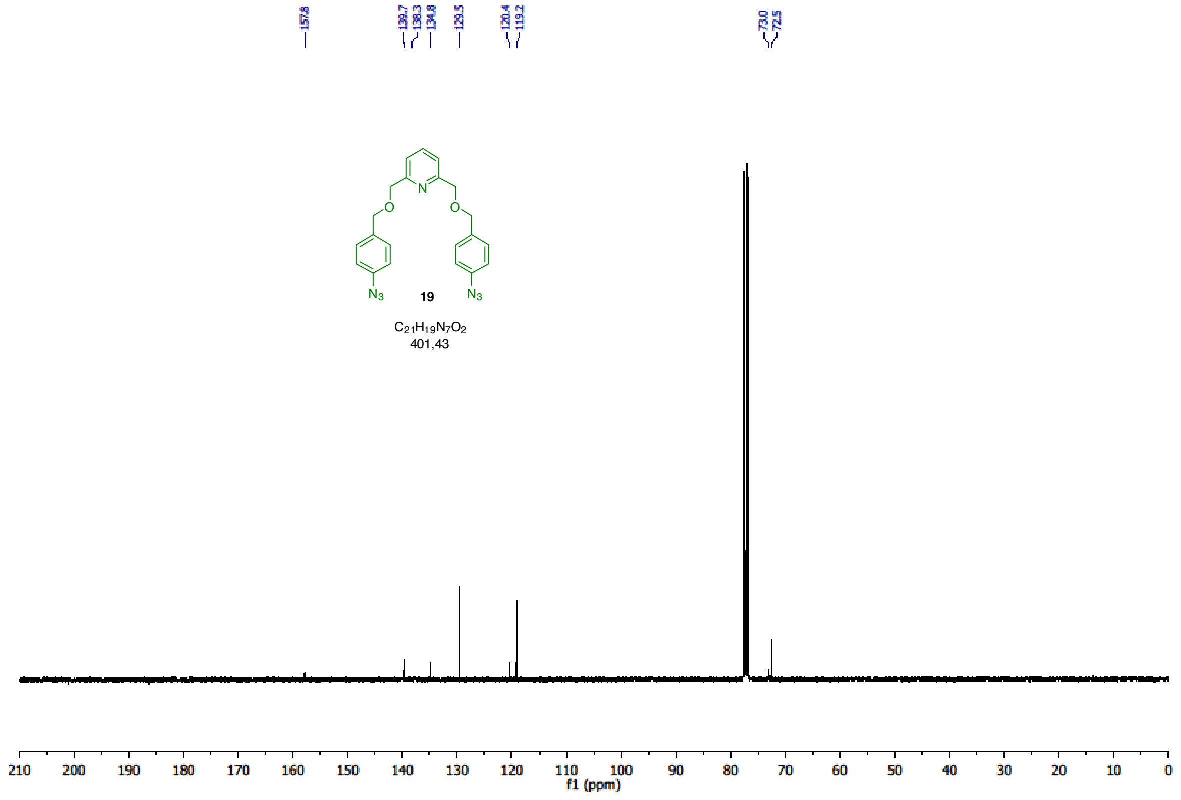


$^{13}$C NMR spectrum (100 MHz, 298 K, $CDCl_3$) of **19**.

***Preparation of compound 7***

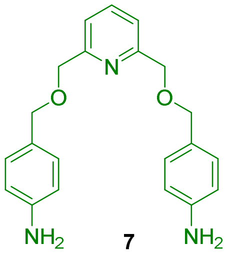

**7**

To a solution of **19** (1g, 2.5 mmol.) in anhydrous THF (74 mL) cooled at 0°C, were added dropwise $Me_2PPh$ (1.06 mL, 7.5 mmol, 3 equiv.) and $NH_4OH$ (33%) (4.4 mL) and stirring was continued for 1 hour at room temperature. The solvent was removed under reduced pressure and the crude material was purified by column chromatography over silica gel (gradient elution 1% to 3% MeOH in $CH_2Cl_2$) to give **7** (0.85 g, 2.43 mmol, 97%) as a shiny yellow solid.

**R*f*:** 0.4 ($CH_2Cl_2$:MeOH 95:5).

**m.p.:** 144°C.

**$^1$H NMR** (400 MHz, DMSO-*d*$_6$): δ 4.37 (s, 4H), 4.48 (s, 4H), 5.07 (bs, 4H), 6.53 (d, *J* = 8.3, 4H), 7.02 (d, *J* = 8.3, 4H), 7.32 (d, *J* = 7.7, 2H), 7.80 (t, *J* = 7.7, 1H).

**$^{13}$C NMR** (100 MHz, DMSO-*d*$_6$): δ 71.8 (x2), 72.3 (x2), 113.5 (x4), 119.7 (x2), 124.8 (x2), 129.4 (x4), 137.3, 148.3 (x2), 157.8 (x2).

**HRESI-MS**: m/z 372.1689 (calcd. for $C_{21}H_{23}N_3O_2Na$ 372.1688 $[M+Na]^+$).

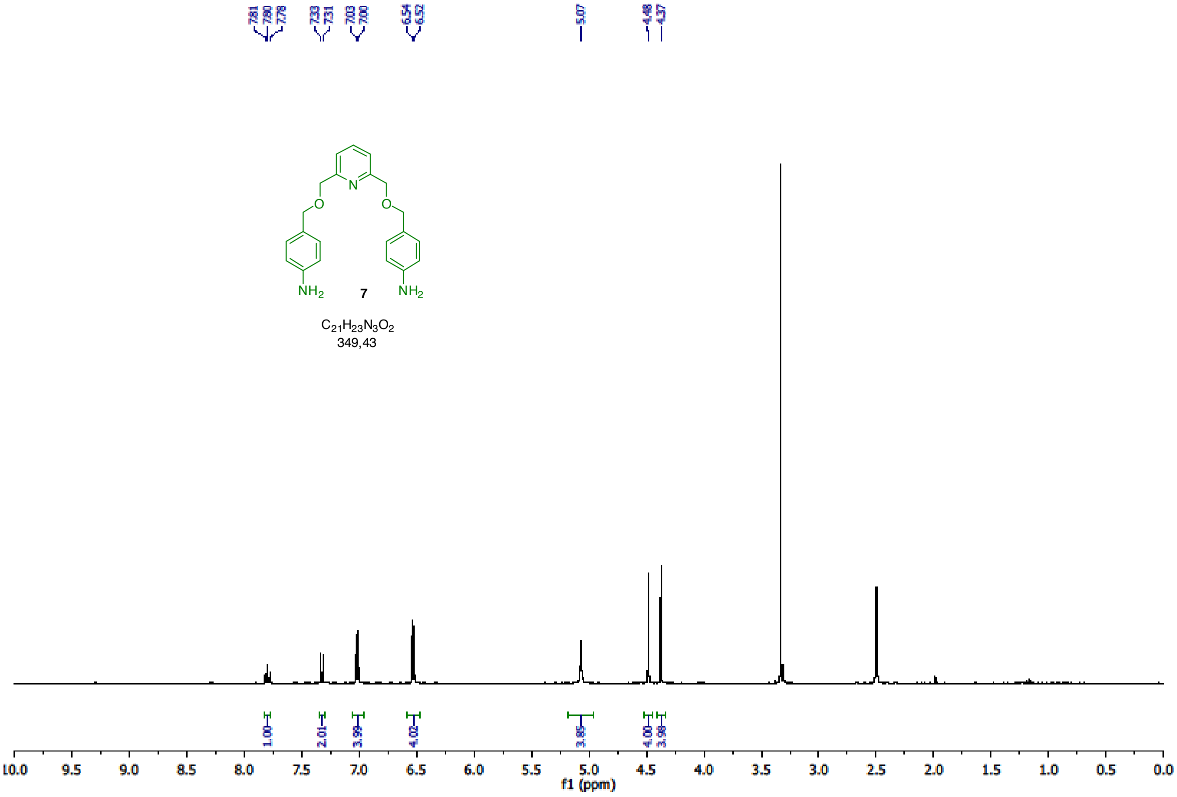


$^{1}$H NMR spectrum (400 MHz, 298 K, DMSO-$d_6$) of **7**.

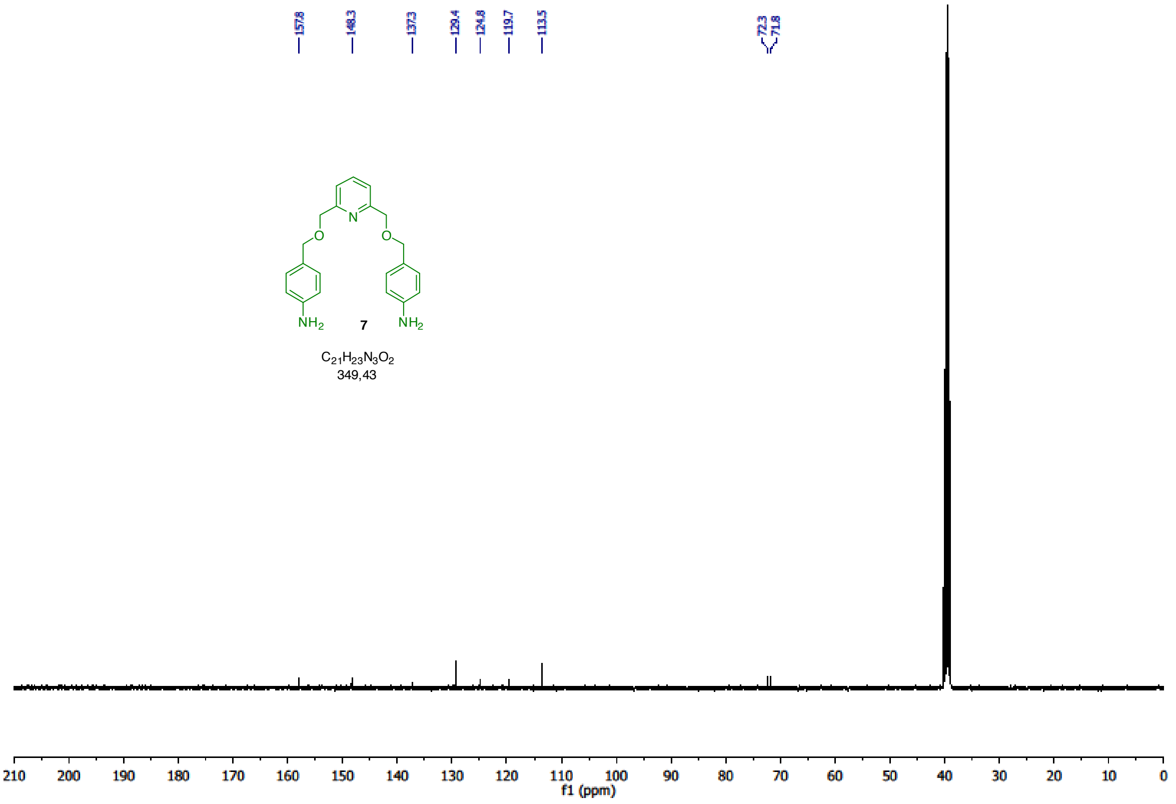


$^{13}$C NMR spectrum (100 MHz, 298 K, DMSO-$d_6$) of **7**.

## *Preparation of compound 8*

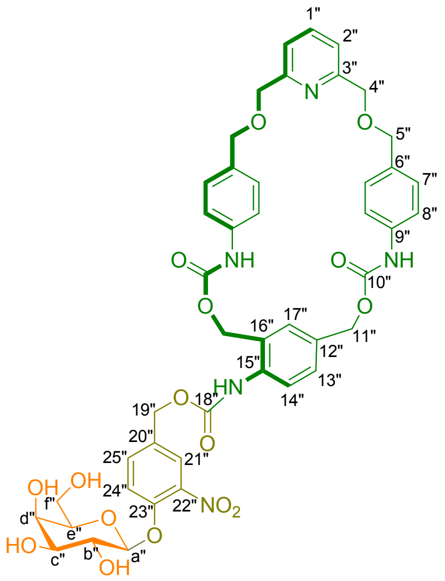


**8**

To a solution of carbonate **6** (400 mg, 0.396 mmol) and aniline **7** (346 mg, 0.991 mmol, 2.25 equiv.) in DMF (4.8 mL), was added HOBt (53 mg, 0.396 mmol) and stirring was continued for 5 hours at room temperature until total disappearance of carbonate **6**. The solution was diluted with DMF (391 mL) and stirring was continued for 96 hours at 30°C. The solvent was removed under reduced pressure and the crude material was purified by column chromatography over silica gel (gradient elution 1.5% to 2% MeOH in $CH_2Cl_2$) to give an unseparable mixture (348 mg) of acetylated macrocycle **20** and aniline **7**. To a solution of this mixture in $CH_2Cl_2$/MeOH 1/2 (10.5 mL) cooled at 0°C, was added MeONa (86 mg, 1.61 mmol) and stirring was continued for 1h15 at 0°C. The solution was neutralized with IRC-50 resin, filtered and concentrated under reduced pressure. The crude material was purified by column chromatography over silica gel (gradient elution 5% to 7% MeOH in $CH_2Cl_2$) to give macrocyle **8** (100 mg, 0.11 mmol, 28%) as a beige solid.

**m.p.:** 145-147°C.

**$^1$H NMR** (500 MHz, $CD_3OD$): δ 3.58 (dd, $J$ = 9.7, 3.3, 1H, $H_{c"}$), 3.71-3.80 (m, 3H, $H_{e"}$, $2H_{f"}$), 3.84 (dd, $J$ = 9.7, 7.7, 1H, $H_{b"}$), 3.90 (d, $J$ = 3.2, 1H, $H_{d"}$), 4.49-4.62 (m, 10H, $4H_{4"}$, $4H_{5"}$, $2H_{19"}$), 5.04 (d, $J$ = 7.7, 1H, $H_{a"}$), 5.16-5.20 (m, 4H, $H_{11"}$), 7.12-7.19 (m, 4H, $H_{7"}$), 7.26-7.33

(m, 5H, $H_{13''}$, $4H_{8''}$), 7.39-7.48 (m, 4H, $H_{2''}$, $H_{14''}$, $H_{24''}$), 7.58 (d, *J* = 1.5, 1H, $H_{17''}$), 7.66 (d, *J* = 8.5, 1H, $H_{25''}$), 7.81 (t, *J* = 7.2, 1H, $H_{1''}$), 7.90 (s, 1H, $H_{21''}$).

**$^{13}$C NMR** (125 MHz, $CD_3OD$): δ 62.3($C_{f''}$), 66.4-66.7 ($2C_{11''}$), 70.1 ($C_{d''}$), 71.9 ($C_{b''}$), 72.4-72.6-73.2 ($2C_{4''}$, $2C_{5''}$, $C_{19''}$), 74.8 ($C_{c''}$), 77.4 ($C_{e''}$), 103.0 ($C_{a''}$), 118.9 ($C_{24''}$), 119.9 ($4C_{8''}$), 121.9 ($C_{14''}$), 123.1 ($2C_{2''}$), 125.7 ($C_{21''}$), 129.5 ($C_{17''}$), 129.9 ($C_{13''}$), 130.1 ($4C_{7''}$), 132.4-133.7 ($2C_{6''}$, $2C_{9''}$, $C_{15''}$, $C_{20''}$), 134.7 ($C_{25''}$), 139.1 ($C_{1''}$), 139.6-141.9-151.2 ($C_{12''}$, $C_{16''}$, $C_{22''}$, $C_{23''}$), 155.6-155.7-156.5-158.8 ($2C_{3''}$, $C_{18''}$, $2C_{10''}$).

**HRESI-MS**: m/z 934.2754 (calcd. for $C_{45}H_{45}N_5O_{16}Na$ 934.2754 $[M+Na]^+$).

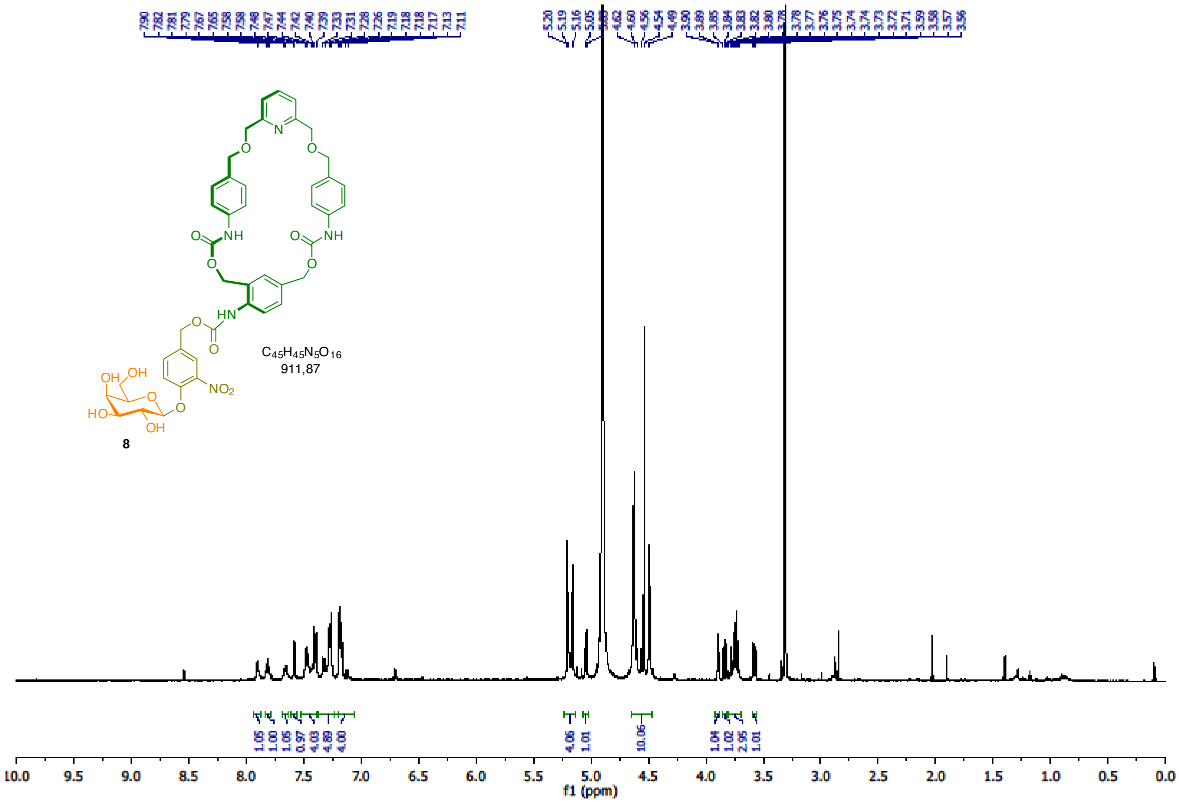


$^{1}$H NMR spectrum (500 MHz, 298 K, $CD_3OD$) of **8**.

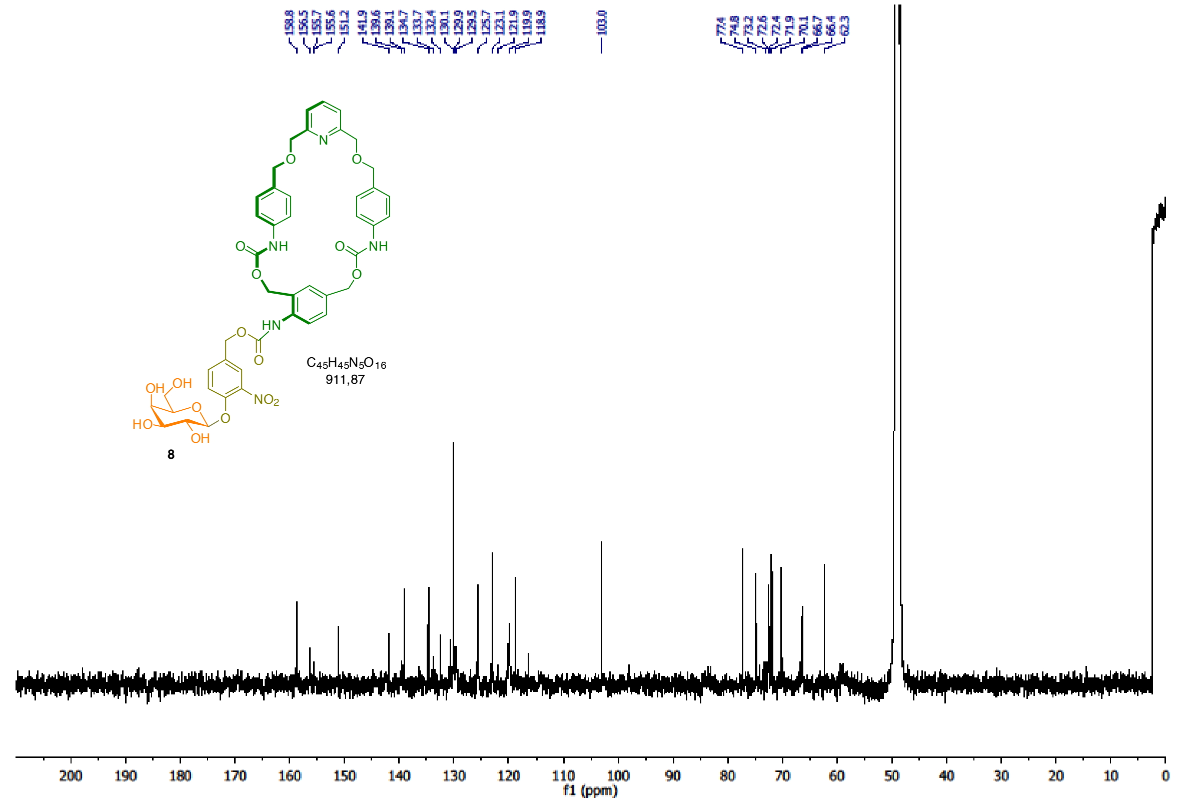


$^{13}$C NMR spectrum (125 MHz, 298 K, $CD_3OD$) of **8**.

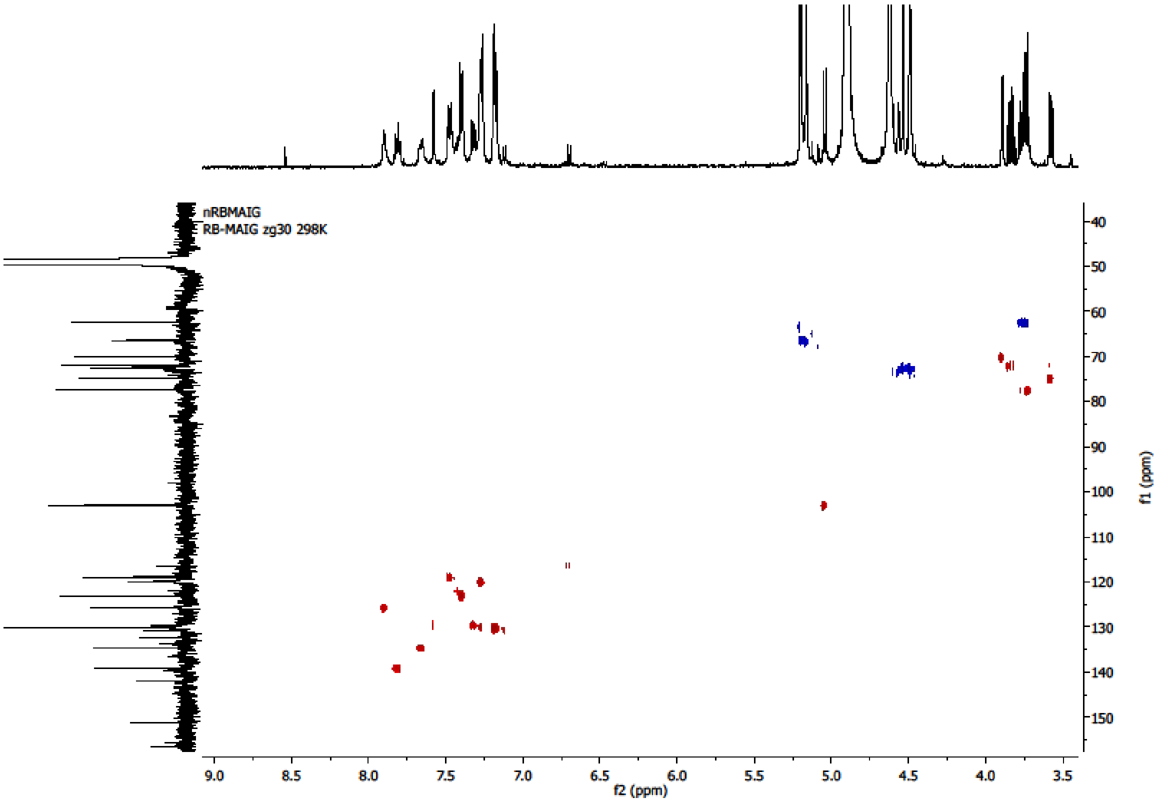


HSQC-edit NMR spectrum (500 MHz, 298 K, $CD_3OD$) of **8**

***Preparation of compound 10***

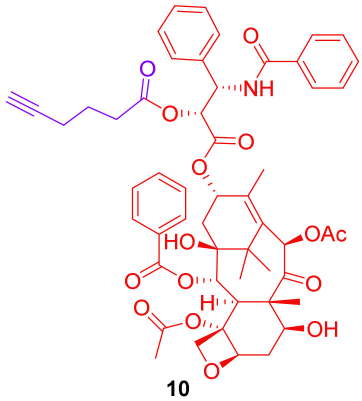


**10**

To a solution of paclitaxel (150 mg, 0.176 mmol) and 5-hexynoic acid (39 $\mu$L, 0.352 mmol, 2 equiv.) in anhydrous THF (10 mL), were added DCC (109 mg, 0.527 mmol, 3 equiv.) and a catalytic amount of DMAP and stirring was continued for 16 hours at room temperature. The solvent was removed under reduced pressure and the crude material was purified on

preparative TLC ($CH_2Cl_2$/MeOH 95/5) to give ester **10** (130 mg, 0.137 mmol, 78%) as a white solid.

**m.p.:** 163°C.

**$^1$H NMR** (400 MHz, $CDCl_3$): δ 1.14-1.37 (m, 8H), 1.58-2.66 (m, 21H), 3.81 (d, *J* = 7.0, 1H), 4.20 (d, *J* = 8.4, 1H), 4.32 (d, *J* = 8.4, 1H), 4.45 (dd, *J* = 10.8, 6.7, 1H), 4.97 (d, *J* = 8.6, 1H), 5.50 (d, *J* = 3.1, 1H), 5.68 (d, *J* = 7.1, 1H), 5.96 (dd, *J* = 9.1, 3.1, 1H), 6.26 (m, 2H), 6.90 (d, *J* = 9.1, 1H), 7.34-7.44 (m, 7H), 7.52 (m, 3H), 7.61 (t, *J* = 7.4, 1H), 7.74 (d, *J* = 7.8, 2H), 8.14 (d, *J* = 7.8, 2H).

**$^{13}$C NMR** (100 MHz, $CDCl_3$): δ 9.7, 14.9, 17.6, 20.9 (x2), 22.3, 22.8 (x2), 25.0, 25.6, 32.4, 35.6 (x2), 43.3, 45.7, 52.8, 58.6, 69.6, 72.1 (x2), 74.1, 75.2, 75.7, 76.6, 77.4, 81.2, 83.1, 84.6, 126.6-127.2-128.6-128.9-129.2-130.3-132.2-133.8 (18C), 168.2-169.9-171.4-172.2 (6C), 204.0.

**HRESI-MS**: m/z 970.3626 (calcd. for $C_{53}H_{57}NO_{15}Na$ 970.3620 $[M+Na]^+$).

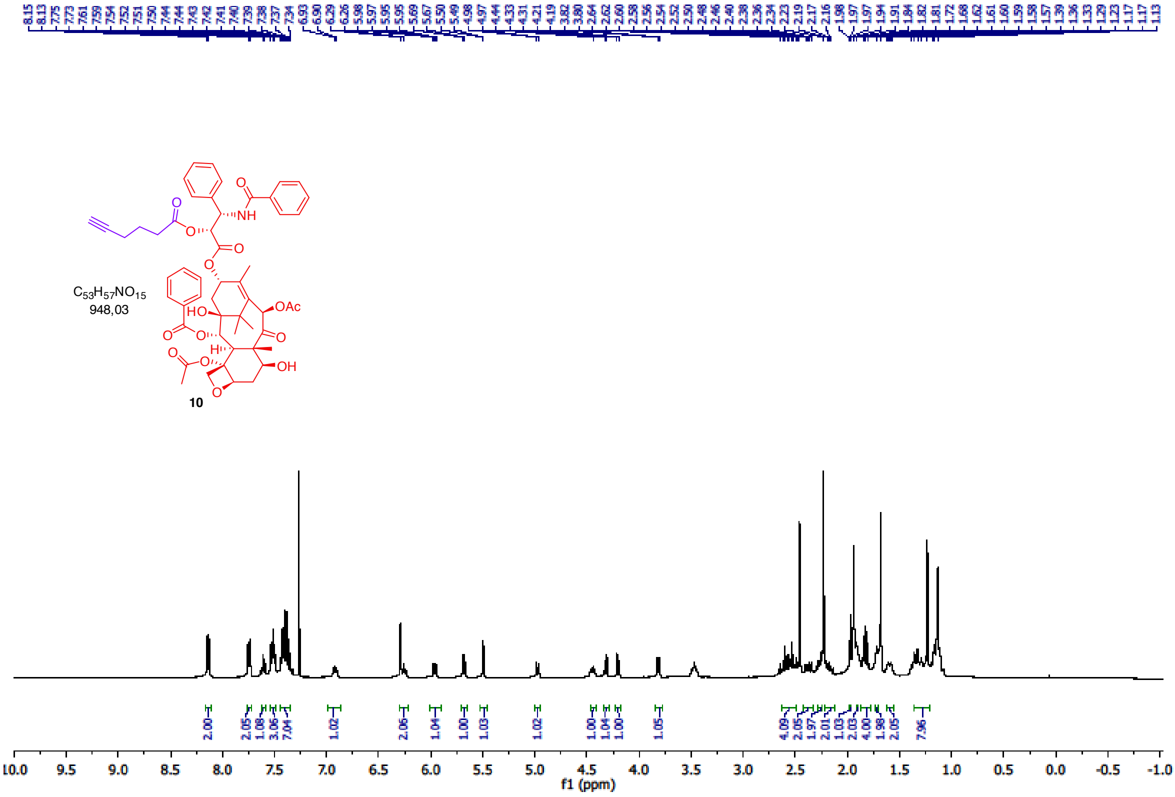


$^{1}$H NMR spectrum (400 MHz, 298 K, $CDCl_3$) of **10**.

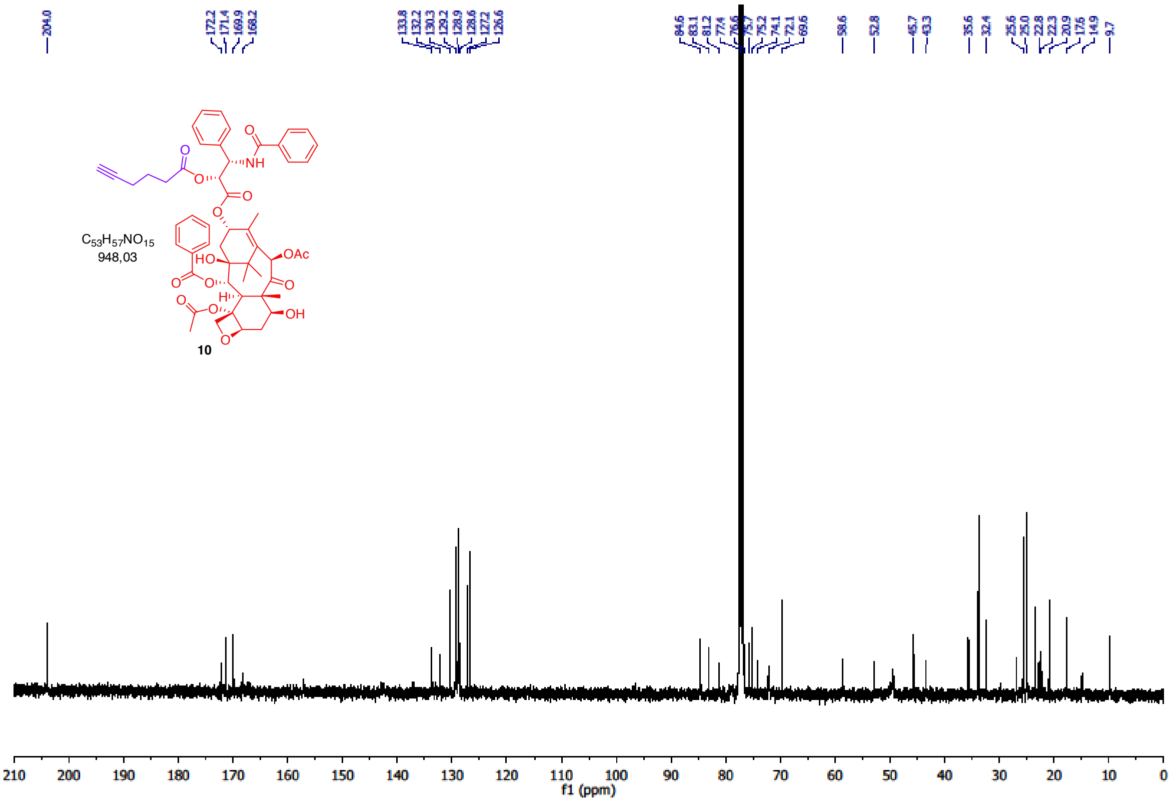


$^{13}$C NMR spectrum (100 MHz, 298 K, $CDCl_3$) of **10**.

***Preparation of compound 22***

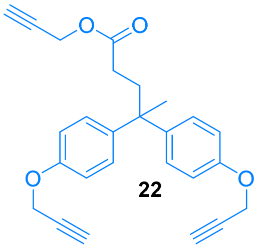

**22**

To a solution of 4,4-bis-(4-hydroxyphenyl) valeric acid **21** (716 mg, 2.5 mmol) in DMF (13 mL), were added $K_2CO_3$ (2.06 g, 15.0 mmol, 6 equiv.) and propargyl bromide (1.7 mL, 15.0 mmol, 6 equiv.). The solution was stirred at 50°C for 22 hours. The reaction was hydrolyzed with water and the solution was extracted with $Et_2O$. The combined organic layers were dried over $MgSO_4$, filtered and concentrated under reduced pressure. The resulting crude material was purified by automatic chromatography (gradient elution 0% to 100% ethyl acetate in petroleum ether) to give **22** (800 mg, 2.0 mmol, 80%) as a colorless wax.

**R*f*:** 0.3 (petroleum ether:ethyl acetate 8:2).

**$^1$H NMR** (400 MHz, $CDCl_3$): δ 1.58 (s, 3H), 2.16 (m, 2H), 2.43 (m, 2H), 2.46 (t, *J* = 2.5, 1H), 2.53 (t, *J* = 2.4, 2H), 4.61 (d, *J* = 2.5, 2H), 4.65 (d, *J* = 2.4, 4H), 6.88 (d, *J* = 8.9, 4H), 7.12 (d, *J* = 8.9, 4H).

**$^{13}$C NMR** (100 MHz, $CDCl_3$): δ 27.8, 30.1, 36.4, 44.7, 52.0, 55.9 (x2), 75.0, 75.6 (x2), 77.8, 78.8 (x2), 114.5 (x4), 128.3 (x4), 141.7 (x2), 155.7 (x2), 173.1.

**HRESI-MS**: m/z 423.1566 (calcd. for $C_{26}H_{24}O_4Na$ 423.1567 $[M+Na]^+$).

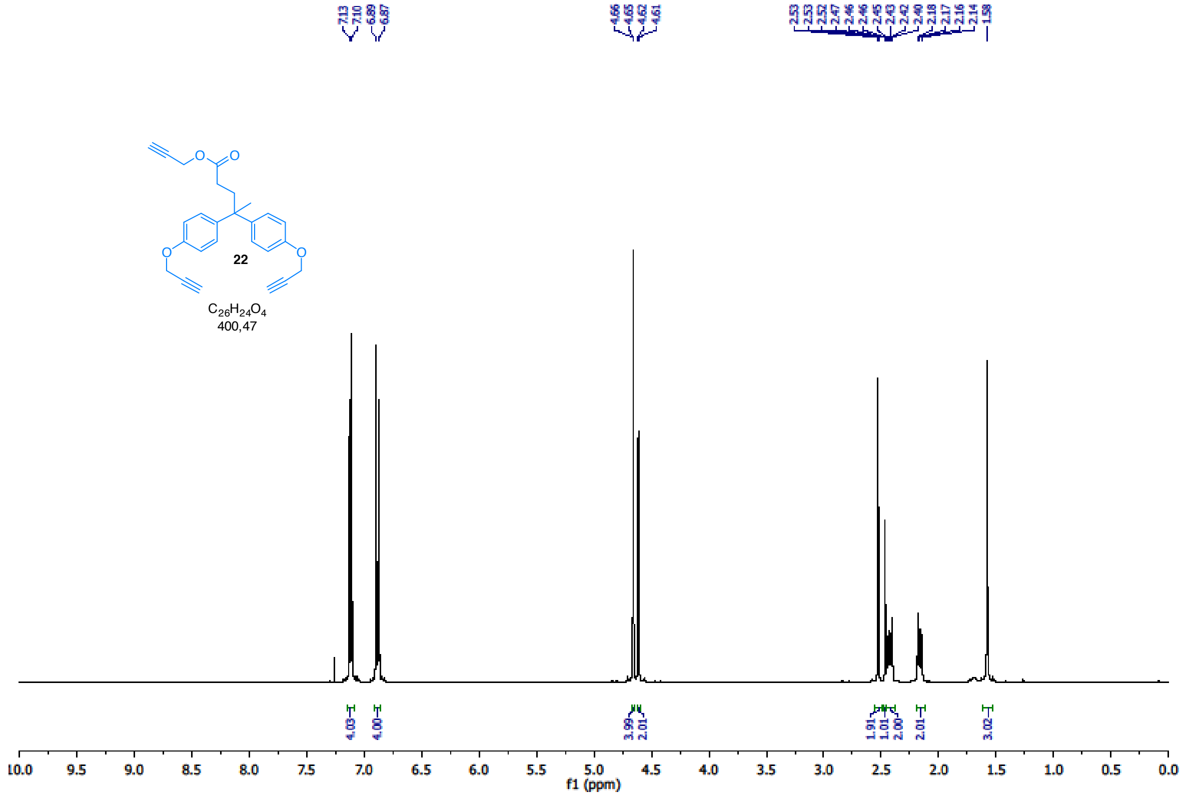


$^{1}$H NMR spectrum (400 MHz, 298 K, $CDCl_3$) of **22**.

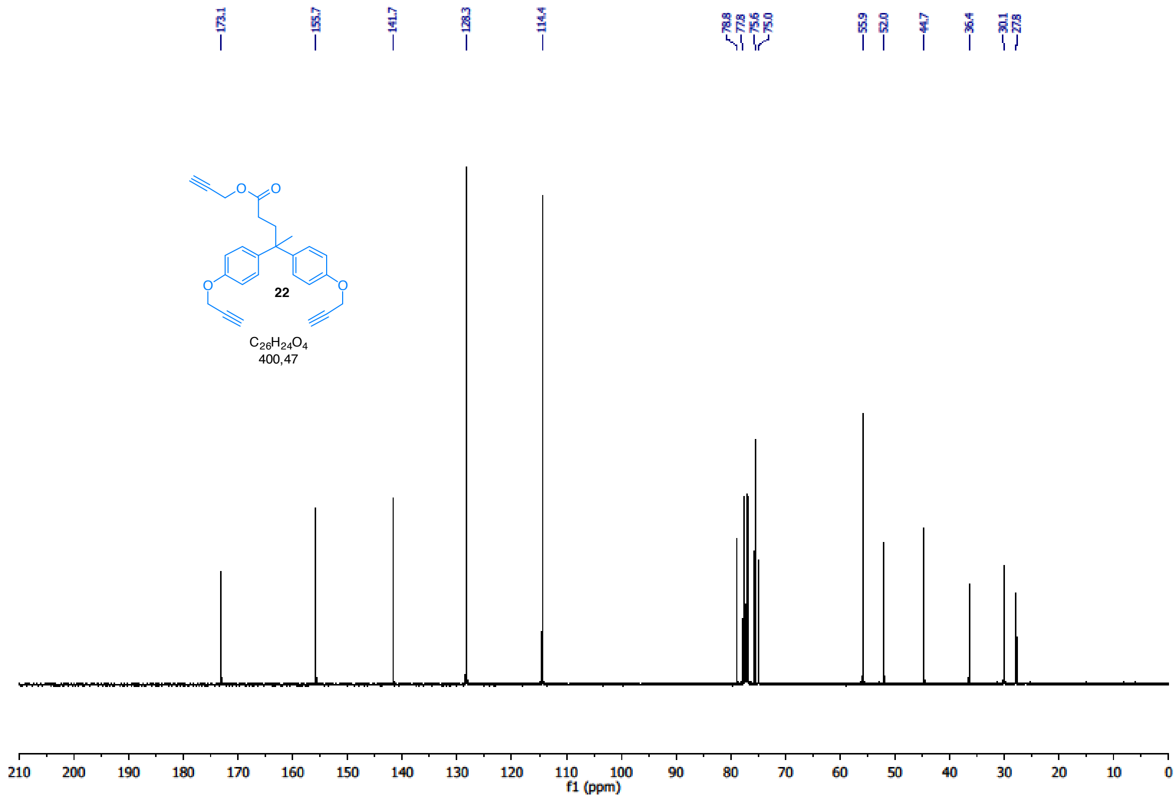


$^{13}$C NMR spectrum (100 MHz, 298 K, $CDCl_3$) of **22**.

### *Preparation of compound 23*

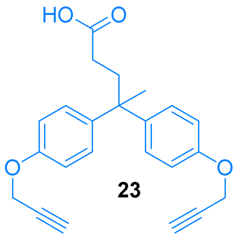

**23**

A solution of ester **22** (800 mg, 2.0 mmol) in 2N NaOH/MeOH 3:2 (10 mL) was stirred at room temperature. After 3 hours, HCl (1N, 12 mL) was added to neutralize the solution. MeOH was removed under reduced pressure and the resulting solution was extracted with ethyl acetate (3x). The combined organic layers were dried over $MgSO_4$, filtered and concentrated under reduced pressure. The resulting crude material was purified by automatic chromatography (gradient elution 0% to 100% ethyl acetate in petroleum ether) to give **23** (684 mg, 1.89 mmol, 94%) as a colorless wax.

**$R_f$:** 0.4 ($CH_2Cl_2$/MeOH 99:1).

**$^1$H NMR** (400 MHz, $CDCl_3$): δ 1.58 (s, 3H), 2.16 (m, 2H), 2.43 (m, 2H), 2.52 (t, *J* = 2.4, 2H), 4.66 (d, *J* = 2.4, 4H), 6.88 (d, *J* = 8.9, 4H), 7.12 (d, *J* = 8.9, 4H).

**$^{13}$C NMR** (100 MHz, $CDCl_3$): δ 27.8, 30.2, 36.4, 44.7, 55.9 (x2), 75.6 (x2), 78.8 (x2), 114.5 (x4), 128.4 (x4), 141.8 (x2), 155.8 (x2), 180.1.

**HRESI-MS**: m/z 385.1413 (calcd. for $C_{23}H_{22}O_4Na$ 385.1410 $[M+Na]^+$).

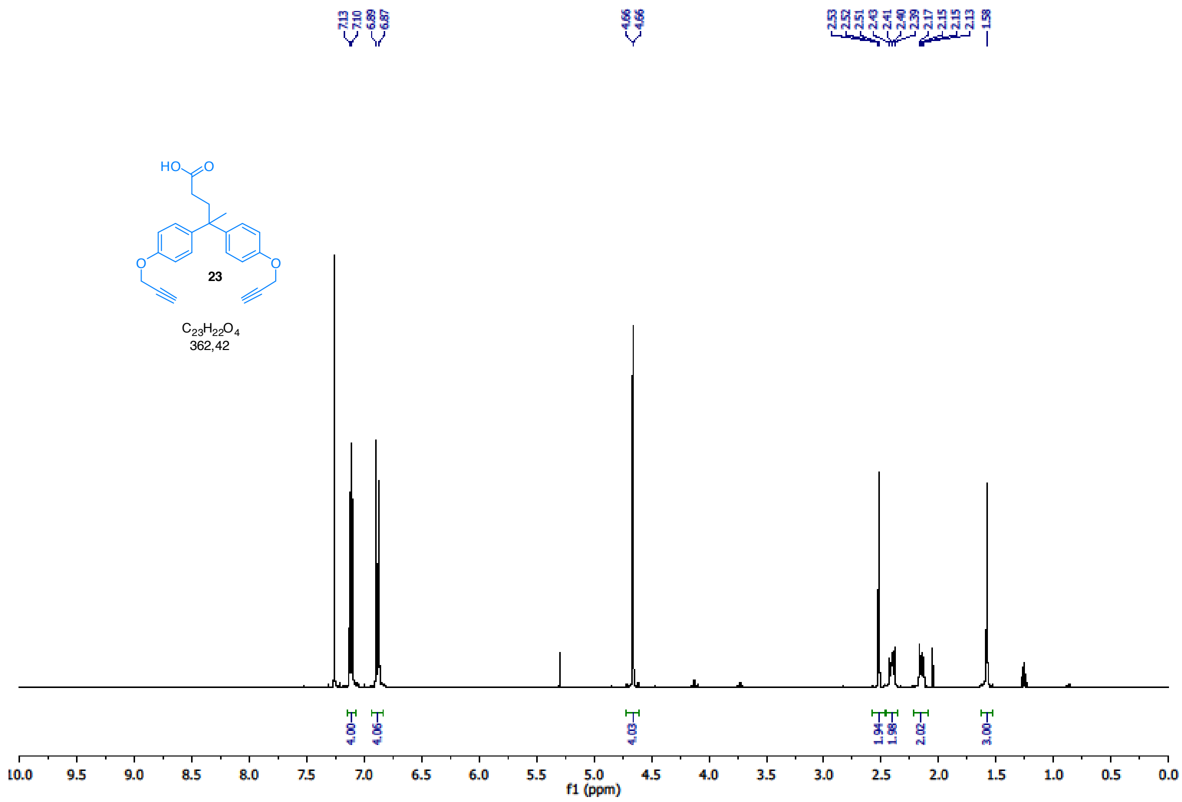


$^1$H NMR spectrum (400 MHz, 298 K, $CDCl_3$) of **23**.

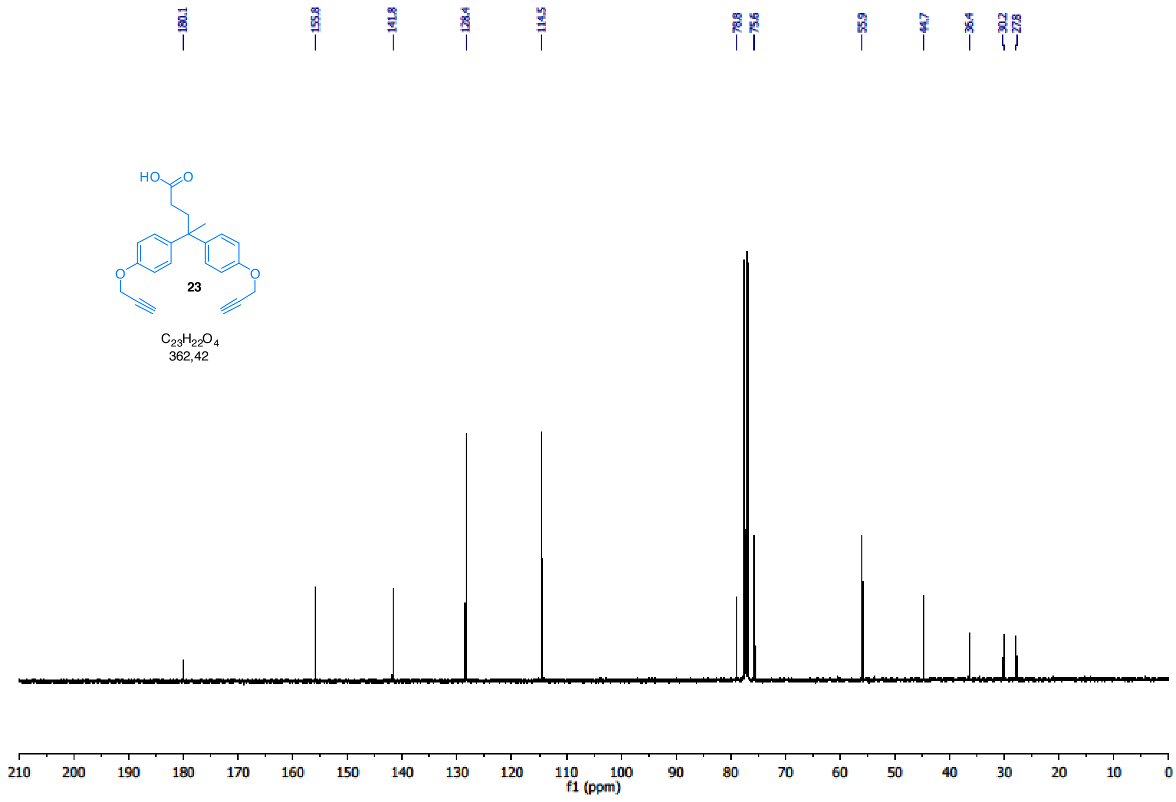


$^{13}$C NMR spectrum (100 MHz, 298 K, $CDCl_3$) of **23**.

### *Preparation of compound 25*

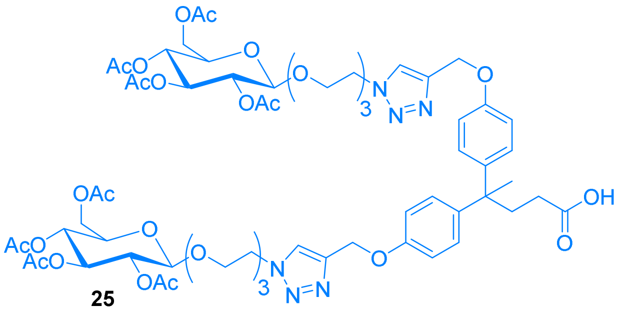

**25**

To a solution of alkyne **23** (114 mg, 0.314 mmol) and 2-[2-(2-azidoethoxy)ethoxy]ethyl-2,3,4,6-tetra-*O*-acetyl-β-D glucoside **24**[4] (317 mg, 0.627 mmol, 2 equiv.) in degassed $CH_2Cl_2$ (3.3 mL), was added $[Cu(CH_3CN)_4](PF_6)$ (59 mg, 0.157 mmol, 0.5 equiv.) and stirring was continued for 48 hours at room temperature. The reaction was quenched with a saturated solution of EDTA acidified with few drops of HCl 1N. The resulting solution was extracted with $CH_2Cl_2$ (3x). The combined organic layers were dried over $MgSO_4$, filtered and concentrated under reduced pressure. The resulting crude material was purified by automatic chromatography (gradient elution 0% to 10% MeOH in $CH_2Cl_2$) to give **25** (336 mg, 0.245 mmol, 78%) as a colorless wax.

**$R_f$:** 0.7 ($CH_2Cl_2$/MeOH 95:5).

**$^1$H NMR** (400 MHz, $CDCl_3$): δ 1.58 (s, 3H), 1.99-2.11 (4s, 24H), 2.14 (t, $J$ = 8.2, 2H), 2.41 (t, $J$ = 8.2, 2H), 3.56-4.0 (m, 26H), 4.12 (d, $J$ = 12.0, 2H), 4.24 (d, $J$ = 12.0, 2H), 4.56 (m, 6H), 4.98 (t, $J$ = 8.6, 2H), 5.07 (t, $J$ = 9.5, 2H), 5.19 (t, $J$ = 9.5, 2H), 6.94 (d, $J$ = 8.8, 4H), 7.12 (d, $J$ = 8.8, 4H), 7.82 (s, 2H).

**$^{13}$C NMR** (100 MHz, $CDCl_3$): δ 20.7 (x4), 20.8 (x2), 20.9 (x2), 27.9, 30.0, 36.6, 44.7, 62.1 (x2), 68.5 (x2), 69.2-70.7 (20C), 71.4 (x2), 71.9 (x2), 72.9 (x2), 77.4 (x2), 101.0 (x2), 114.6 (x2), 128.5 (x2), 141.7 (x2), 156.4 (x2), 169.5 (x2), 169.6 (x2), 170.4 (x2), 170.8 (x2), 177.2.

**HRESI-MS**: m/z 1395.5231 (calcd. for $C_{63}H_{84}N_6O_{28}Na$ 1395.5226 $[M+Na]^+$).

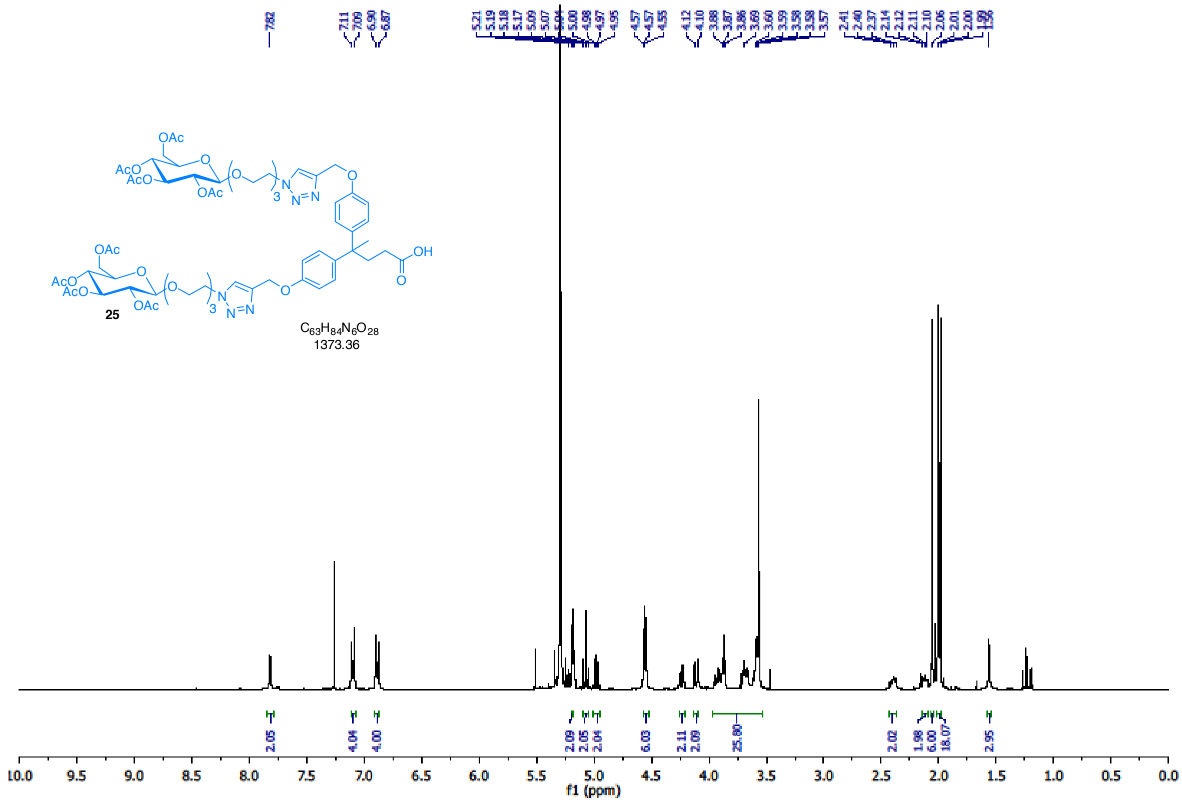


$^1$H NMR spectrum (400 MHz, 298 K, $CDCl_3$) of **25**.

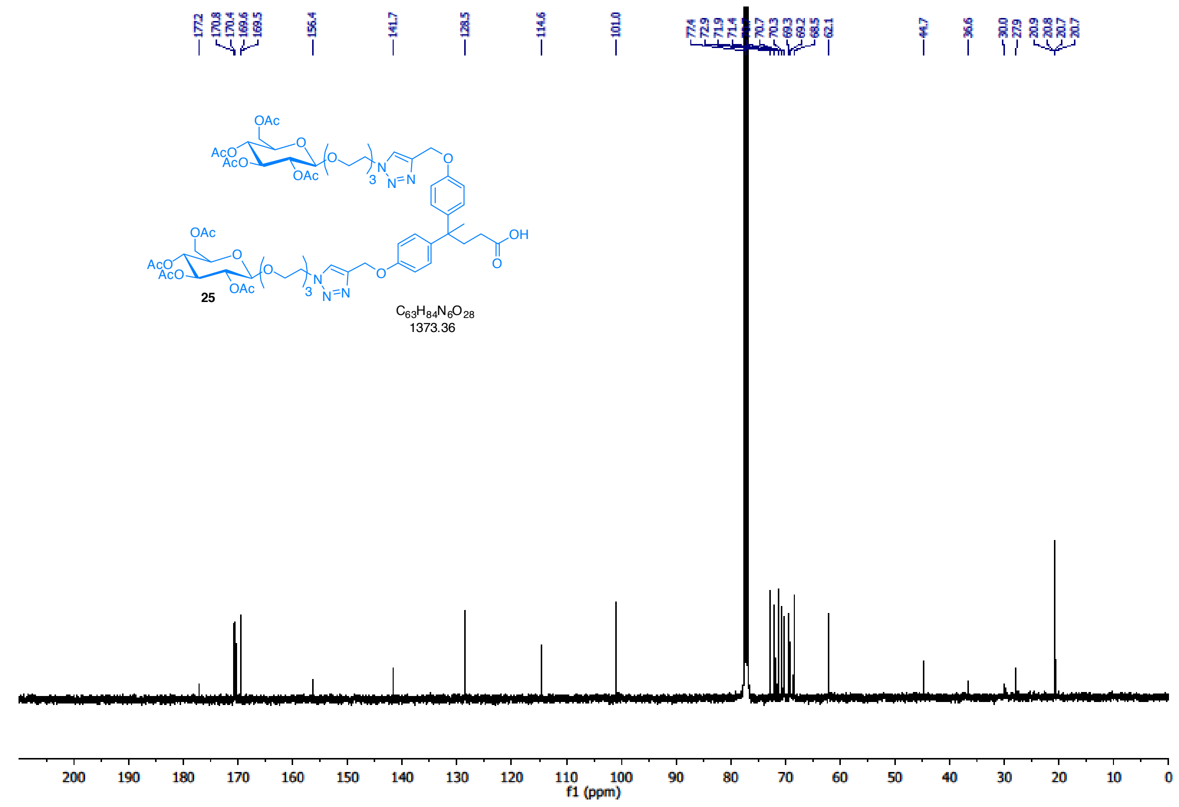


$^{13}$C NMR spectrum (100 MHz, 298 K, $CDCl_3$) of **25**.

***Preparation of compound 27***

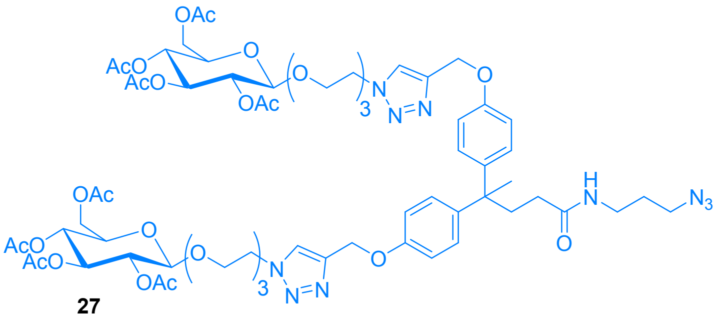


**27**

To a solution of **25** (200 mg, 0.146 mmol) and 3-amino-1-azide-propane **26**[5] (29 mg, 0.29 mmol, 2 equiv.) in dry DMF (10 mL), was added EDC (56 mg, 0.292 mmol, 2 equiv.) and DMAP (4 mg, 0.2 equiv.). Stirring was continued for 24 hours at room temperature and the mixture was hydrolyzed with water and extracted with ethyl acetate. The combined organic layers were dried over $MgSO_4$, filtered and concentrated under reduced pressure. The resulting crude material was purified by automatic chromatography (gradient elution 0% to 10% MeOH in $CH_2Cl_2$) to give **27** (125 mg, 0.086 mmol, 59%) as a colorless wax.

**R*f*:** 0.5 ($CH_2Cl_2$/MeOH 97:3).

**$^1$H NMR** (400 MHz, $CDCl_3$): δ 1.57 (s, 3H) ,1.75 (qi, *J* = 6.6, 2H), 1.92 (m, 2H), 1.99-2.06 (4s, 24H), 2.40 (m, 2H), 3.27 (m, 2H), 3.33 (t, *J* = 6.6, 2H), 3.58-3.73 (m, 16H), 3.88 (t, *J* = 5.1, 4H), 3.91-3.96 (m, 2H), 4.12 (dd, *J* = 12.3, 2.3, 2H), 4.24 (dd, *J* = 12.3, 4.6, 2H), 4.57 (m, 6H), 4.99 (dd, *J* = 9.6, 8.0, 2H), 5.08 (t, *J* =9.6, 2H), 5.18 (s, 4H), 5.20 (t, *J* = 9.6, 2H), 5.62 (t, *J* = 6.6, 1H), 6.90 (d, *J* = 8.8, 4H), 7.11 (d, *J* = 8.8, 4H), 7.82 (s, 2H).

**$^{13}$C NMR** (100 MHz, $CDCl_3$): δ 20.8-20.9 (x8), 28.0, 28.9, 32.8, 37.2 (x2), 44.9, 49.5, 50.6 (x2), 62.1 (x4), 68.5 (x2), 69.3 (x4), 69.6 (x2), 70.4-70.7 (x4), 71.4 (x2), 71.9 (x2), 72.9 (x2), 101.0 (x2), 114.4 (x4), 124.2 (x2), 128.5 (x4), 141.8 (x2), 144.1 (x2), 156.5 (x2), 169.5 (x2), 169.6 (x2), 170.4 (x2), 170.8 (x2).

**HRESI-MS**: m/z 1455.6068 (calcd. for $C_{66}H_{91}N_{10}O_{27}$ 1455.6050 $[M+H]^+$).

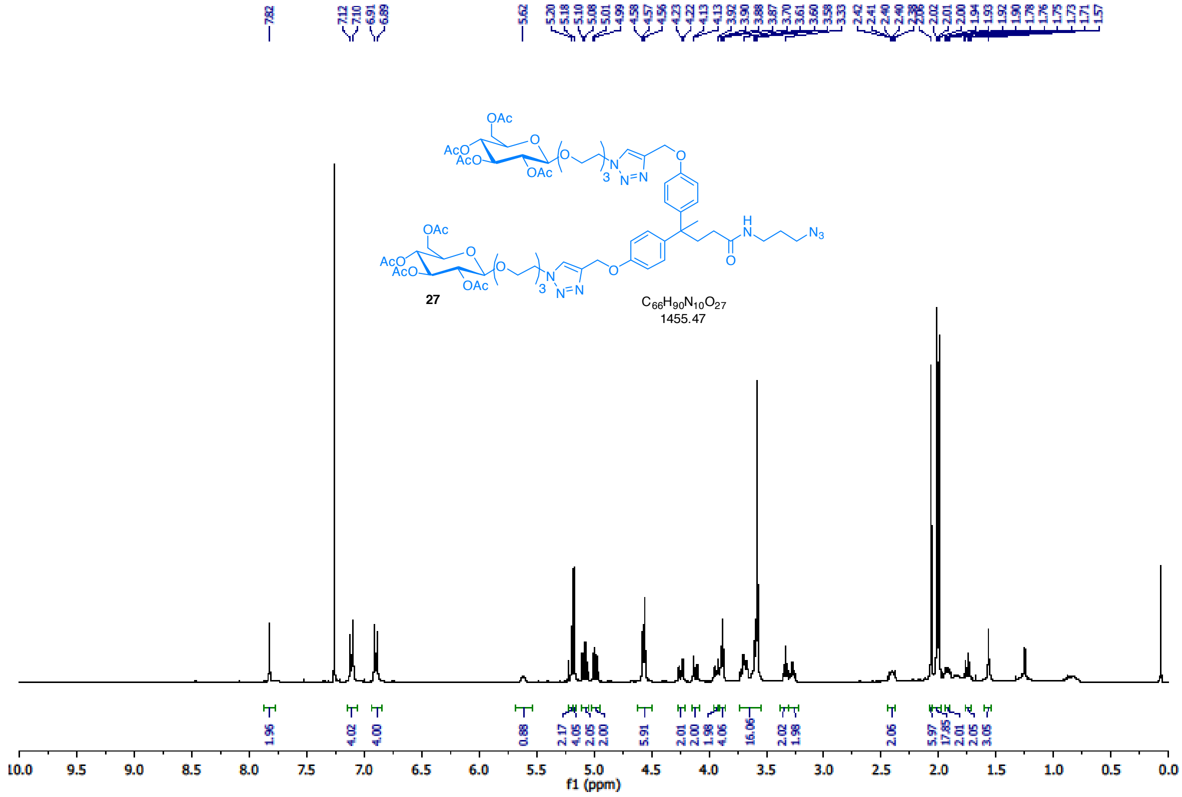

$^1$H NMR spectrum (400 MHz, 298 K, CDCl$_3$) of **27**.

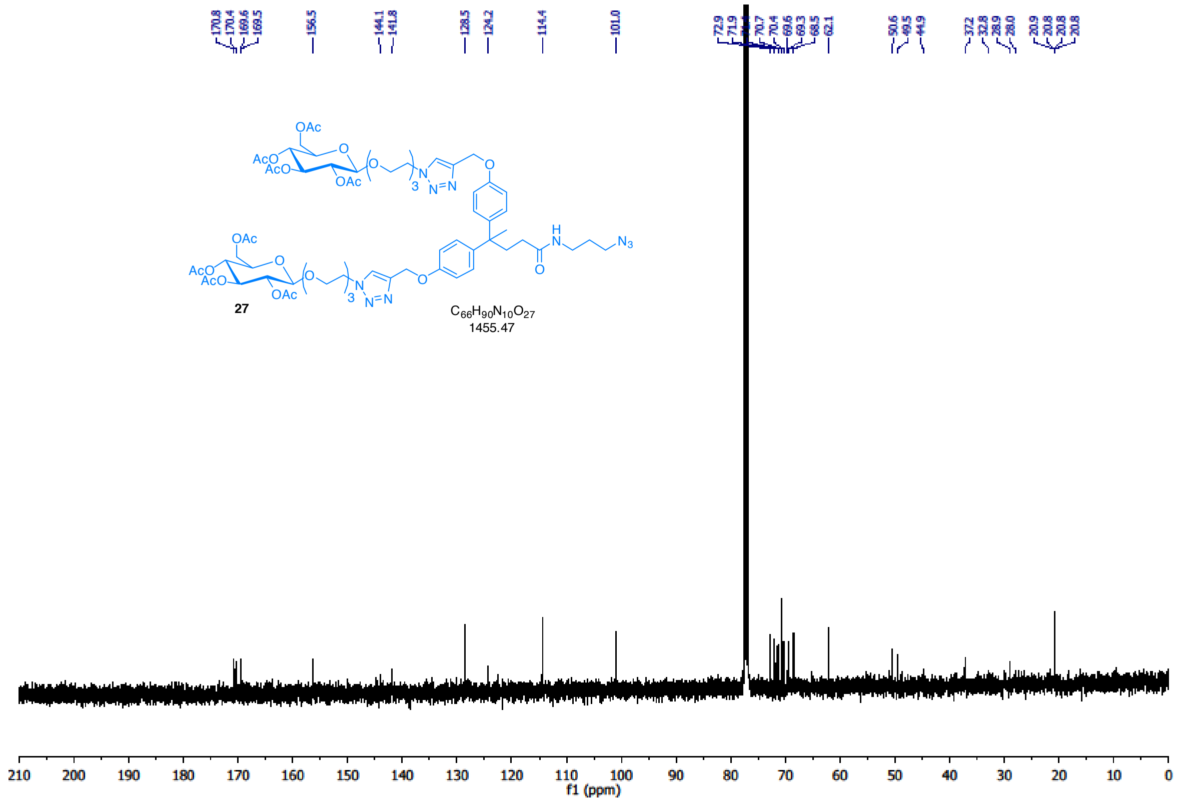

$^{13}$C NMR spectrum (100 MHz, 298 K, CDCl$_3$) of **27**.

***Preparation of compound 9***

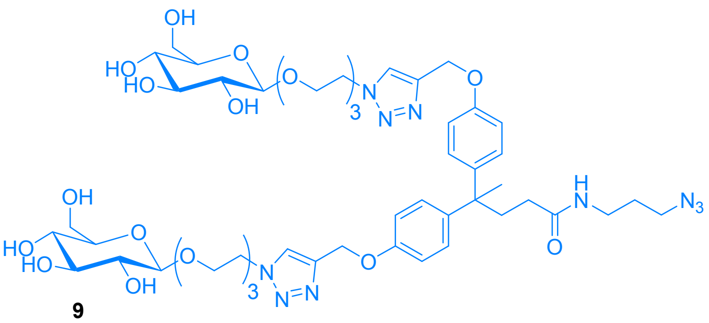

**9**

To a solution of **27** (164 mg, 0.113 mmol) in MeOH (5.7 mL), was added MeONa (37 mg, 0.676 mmol, 6 equiv.). Stirring was continued for 18 hours at room temperature and the solution was neutralized with Amberlite® Weakly acidic Cation Exchanger hydrogen form during 20 minutes. The solution was filtered through a pad of cotton and the solvent was removed under reduced pressure to give **9** without further purification (126 mg, 0.113 mmol, 100%) as a colorless wax.

**R*f*:** 0.3 ($CH_2Cl_2$/MeOH 8:2).

**$^1$H NMR** (400 MHz, $CD_3OD$): δ 1.59 (s, 3H), 1.71 (qi, *J* = 6.8, 2H), 1.99 (m, 2H), 2.38 (m, 2H), 3.17-3.37 (m, 12H), 3.60-3.71 (m, 16H), 3.84 (dd, *J* = 12.0, 2.0, 2H), 3.90 (t, *J* = 5.0, 4H), 3.98 (m, 2H), 4.27 (d, *J* = 7.8, 2H), 4.60 (t, *J* = 5.0, 4H), 5.15 (s, 4H), 6.93 (d, *J* = 8.9, 4H), 7.14 (d, *J* = 8.9, 4H), 8.13 (s, 2H).

**$^{13}$C NMR** (100 MHz, $CD_3OD$): δ 28.3, 29.7, 33.1, 37.8, 38.7, 45.8, 50.1, 51.5 (x2), 62.4 (x2), 62.8 (x2), 69.7 (x2), 70.3 (x2), 71.4 (x6), 71.6-78.0 (8C), 104.5 (x2), 115.5 (x4), 126.3 (x2), 129.5 (x4), 143.2 (x2), 145.0 (x2), 157.8 (x2), 176.4.

**HRESI-MS**: m/z 1141.5033 (calcd. for $C_{50}H_{74}N_{10}O_{19}$ 1141.5024 $[M+Na]^+$).

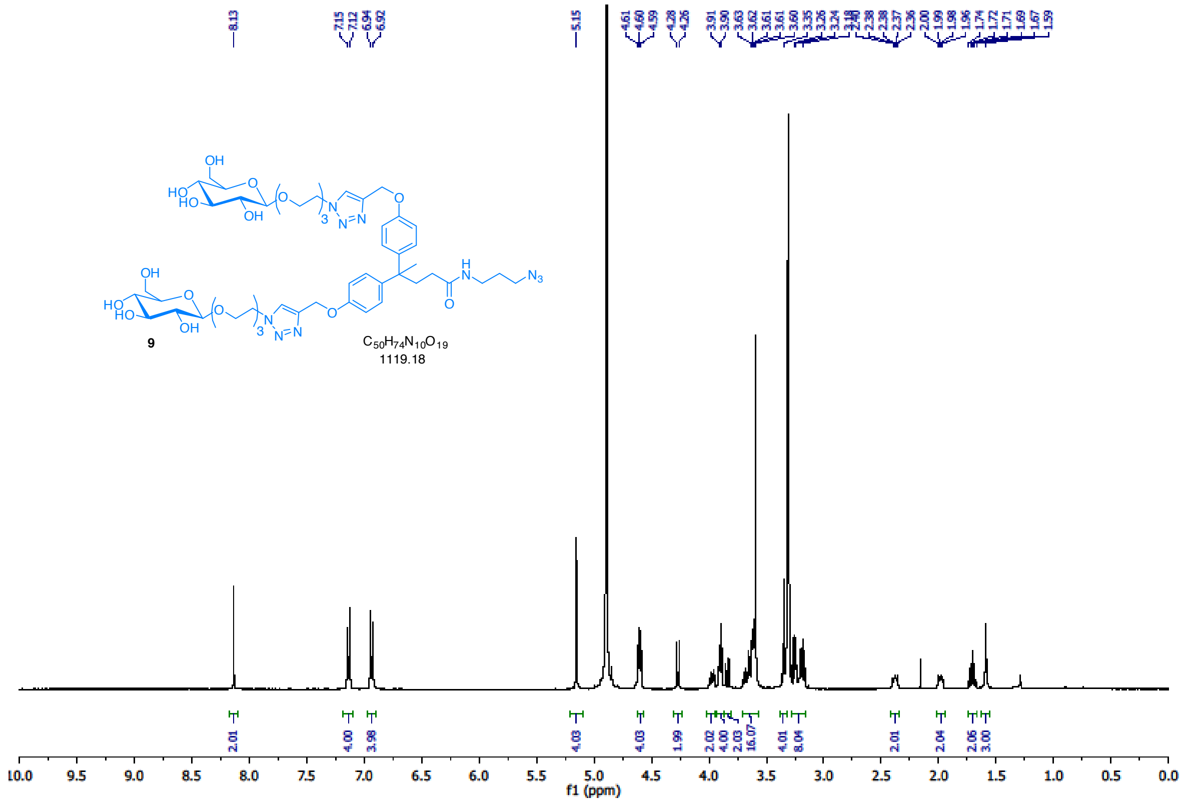

$^{1}$H NMR spectrum (400 MHz, 298 K, $CD_3OD$) of **9**.

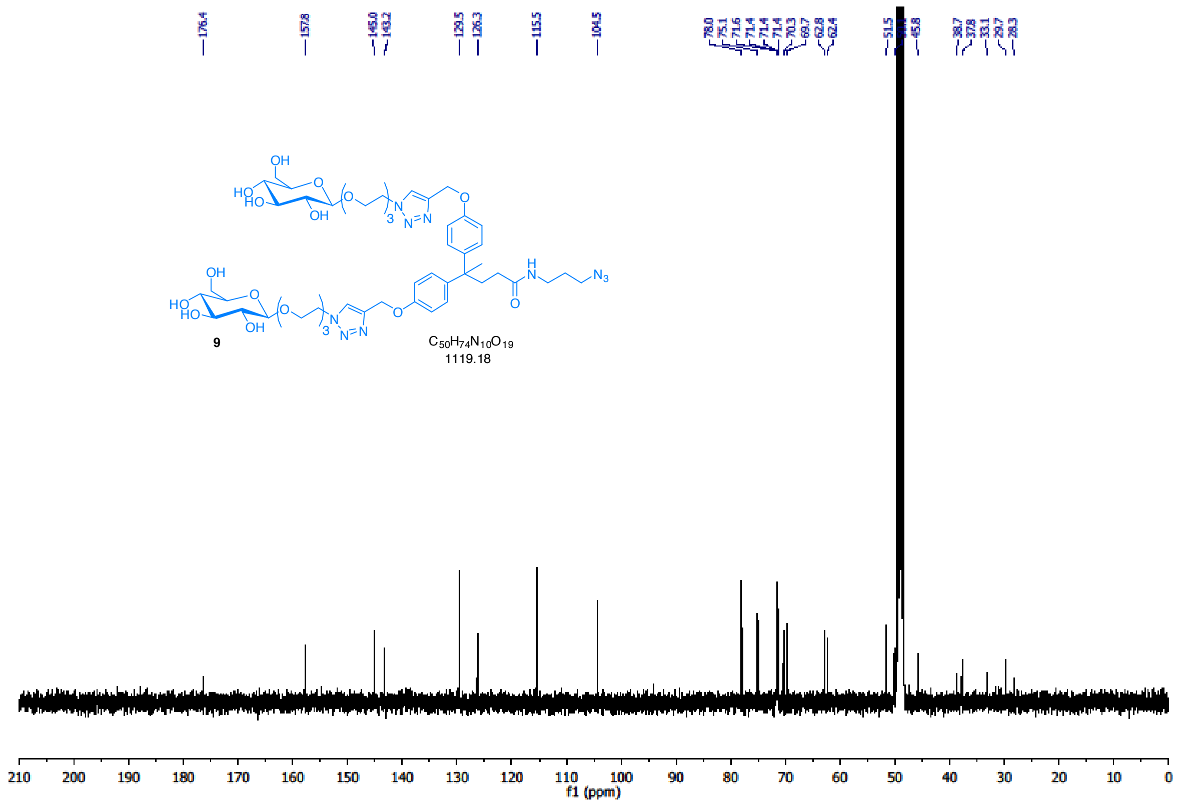

$^{13}$C NMR spectrum (100 MHz, 298 K, $CD_3OD$) of **9**.

## *Preparation of compound 4*

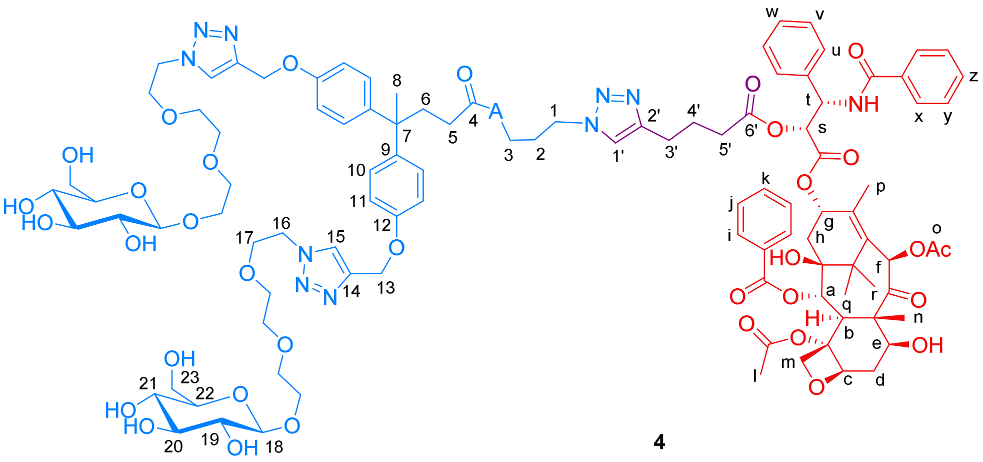

**4**

To a solution of azide **9** (22 mg, 18.7 $\mu$mol) and alkyne **10** (17.7 mg, 18.7 $\mu$mol) in $CH_2Cl_2$/MeOH 83:17 (0.8 mL), was added $[Cu(CH_3CN)_4](PF_6)$ (6.3 mg, 16.8 $\mu$mol, 0.9 equiv.) and stirring was continued for 4 days at room temperature. The mixture was concentrated and diluted with water. The resulting solution was extracted with $CHCl_3$ and the combined organic layers were dried over $MgSO_4$, filtered and concentrated under reduced pressure. The resulting crude material was purified by semi-preparative reverse-phase HPLC. Crude compound (10 mg) was dissolved in a minimum amount of MeOH and filtered through a syringe filter (membrane Nylon, porosity 0.45$\mu$m, diameter 13 mm). Solvent flow 4 mL.min$^{-1}$ was applied to a semi-preparative column ACE® C18-AR (100x10 mm, 5 $\mu$m). Gradient eluent was composed of A ($H_2O$) and B ($CH_3CN$). Method: linear gradient beginning with A/B 80:20 reaching A/B 0:100 within 30 minutes. After lyophilization, compound **4** was obtained as a white powder (2.3 mg, 1.1 $\mu$mol, 6%).

**$^1$H NMR** (500 MHz, $CD_3OD$): δ 1.12-1.13 (2s, 6H. $H_q$, $H_r$), 1.56 (s, 3H, $H_8$), 1.65 (s, H, $3H_n$), 1.80-2.01 (m, 13H, $H_2$, $H_5$, $H_h$, $4H_{CH2}$, $H_o$), 2.15 (s, 3H, $H_p$), 2.32-2.36 (m, 2H, $H_6$), 2.39 (s, 3H, $H_l$), 2.46-2.49 (m, 2H, $H_d$), 2.69-2.73 (m, 2H, $H_{CH2}$), 3.11 (t, $J$ = 6.7, 2H, $H_3$), 3.17-3.37 (m, 8H, $8H_{glucose}$), 3.58-3.69 (m, 16H, $2H_{23}$,. $14H_{CH2\ PEG}$), 3.80-3.85 (m, 3H, $H_b$, $2H_{CH2\ PEG}$), 3.88 (t, $J$ = 5.0, 4H, $H_{16}$), 3.94-3.98 (m, 2H, $2H_{23}$), 4.18 (s, 2H, $H_m$), 4.27 (dd, $J$ = 7.8, 0.6, 2H,

$H_{18}$), 4.32-4.35 (m, 3H, $H_e$, $H_1$), 4.59 (t, *J* = 5.0, 4H, $H_{17}$), 5.00 (m, 1H, $H_c$), 5.13 (s, 4H, $H_{13}$), 5.46 (d, *J* = 6.5, 1H, $H_s$), 5.64 (d, *J* = 7.2, 1H, $H_a$), 5.84 (d, *J* = 6.5, 1H, $H_t$), 6.06 (t, *J* = 9.1, 1H, $H_g$), 6.45 (s, 1H, $H_f$), 6.91 (d, *J* = 8.6, 4H, $H_{10}$), 7.10 (d, *J* = 8.6, 4H, $H_{11}$), 7.25 (t, *J* = 7.4, 1H, $H_z$), 7.40-7.44 (m, 4H, $H_v$, $H_y$), 7.49-7.52 (m, 3H, $H_w$, $H_x$), 7.59 (t, *J* = 7.7, 2H, $H_j$), 7.66-7.69 (m, 1H, $H_k$), 7.71 (s, 1H, $H_{1'}$), 7.78-7.80 (m, 2H, $H_u$), 8.10-8.12 (m, 4H, $H_i$, $H_{15}$).

**$^{13}$C NMR** (125 MHz, $CD_3OD$): δ 10.5 ($C_n$), 15.0 ($C_o$), 20.8 ($C_p$), 22.4 ($C_q$), 23.3 ($C_l$), 25.2 ($C_{CH2}$), 25.6 ($C_{CH2}$), 26.1 ($C_{CH2}$), 26.9 ($C_r$), 28.3 ($C_8$), 31.0 ($C_{CH2}$), 33.1 ($C_{CH2}$), 33.6 ($C_d$), 34.7 ($C_{CH2}$), 36.4 ($C_{quat.\ paclitaxel}$), 37.5 ($C_3$), 38.6 ($C_6$), 44.6 ($C_{quat.\ paclitaxel}$), 45.8 ($C_7$), 47.9 ($C_b$), 48.8 ($C_1$), 51.5 ($2C_{17}$), 55.3 ($C_t$), 59.2 ($C_{quat.\ paclitaxel}$), 62.3 ($2C_{13}$), 62.7 ($C_{CH2\ PEG}$), 69.7 ($2C_{23}$), 70.3 ($2C_{16}$), 71.3-71.4 ($3C_{CH2\ PEG}$), 71.6 ($C_{glucose}$), 72.3 ($C_e$), 72.9 ($C_g$), 75.0 ($C_{glucose}$), 75.9 ($C_s$), 76.2 ($C_a$), 76.8 ($C_f$), 77.4 ($C_m$), 77.9-78.0 ($2C_{glucose}$), 82.2 ($C_{quat.\ paclitaxel}$), 85.9 ($C_c$), 104.4 ($2C_{18}$), 115.4 ($4C_{10}$), 123.8 ($C_{1'}$), 126.3 ($2C_{15}$), 128.6-128.7 ($C_u$, $C_w$), 129.5-129.6-129.7 ($C_v$, $C_y$, $C_z$, $C_{11}$), 130.1 ($C_{quat.\ aro.\ paclitaxel}$), 131.2 ($C_i$), 131.4 ($C_{quat.\ aro.\ paclitaxel}$), 132.9 ($C_x$), 134.7 ($C_k$), 134.9 ($C_j$), 135.5-138.4-142.4-143.1 ($2C_{C=C\ paclitaxel}$, $C_{quat.\ aro.\ paclitaxel}$, $C_9$), 144.9 ($2C_{14}$), 148.0 ($C_{2'}$), 157.7 ($2C_{12}$), 167.6-170.5-170.6-171.3-171.6-174.0 ($C_{6'}$, [$2C_{C=O\ acetate}$, $2C_{C=O\ esters}$, $C_{C=O\ amide}$]$_{paclitaxel}$), 176.4 ($C_4$), 205.2 ($C_{C=O\ paclitaxel}$).

**HRESI-MS**: m/z 2066.8865 (calcd. for $C_{103}H_{132}N_{11}O_{34}$ 2066.8860 $[M+H]^+$).

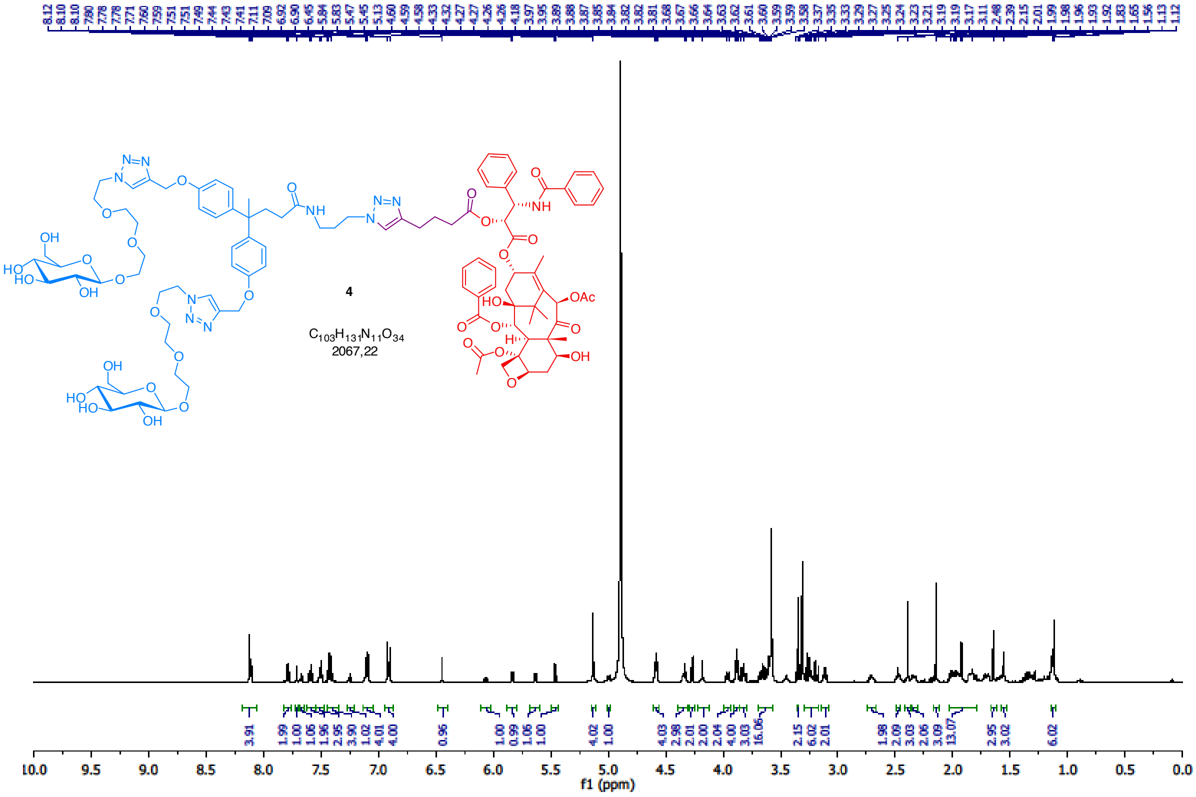

$^{1}$H NMR spectrum (500 MHz, 298 K, $CD_3OD$) of **4**.

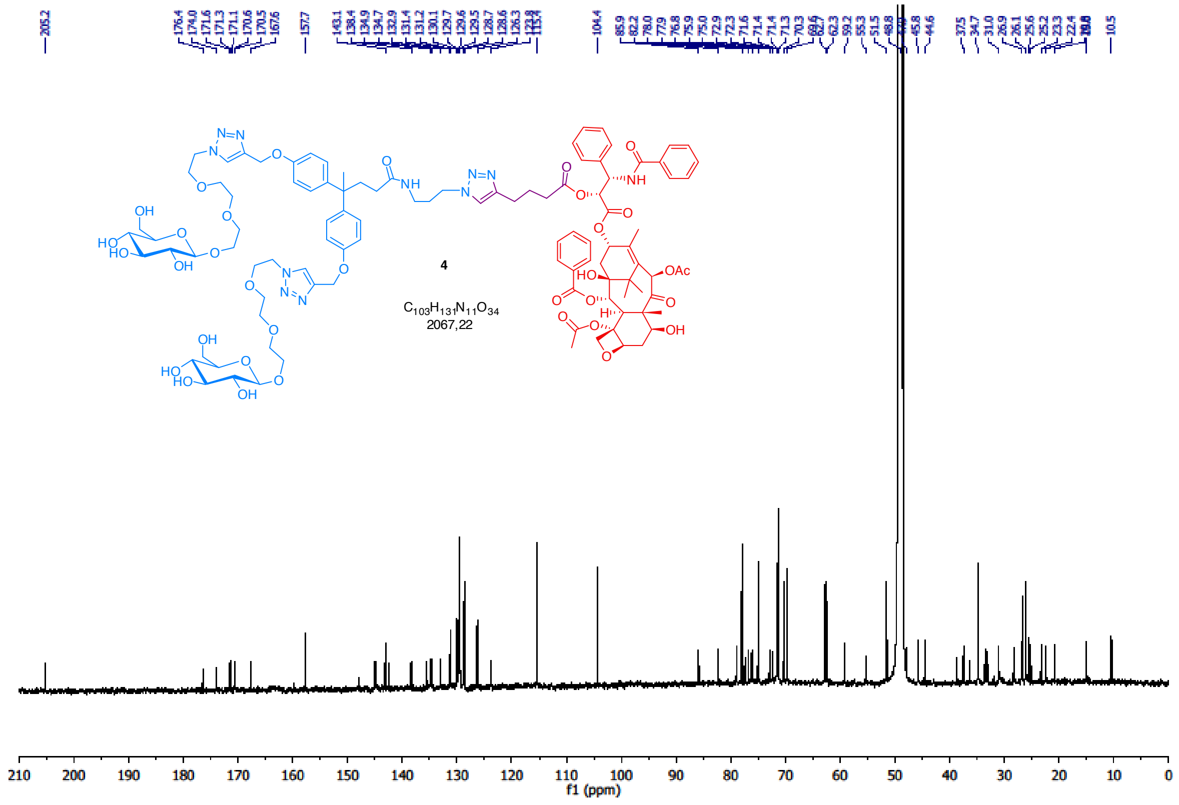

$^{13}$C NMR spectrum (125 MHz, 298 K, $CD_3OD$) of **4**.

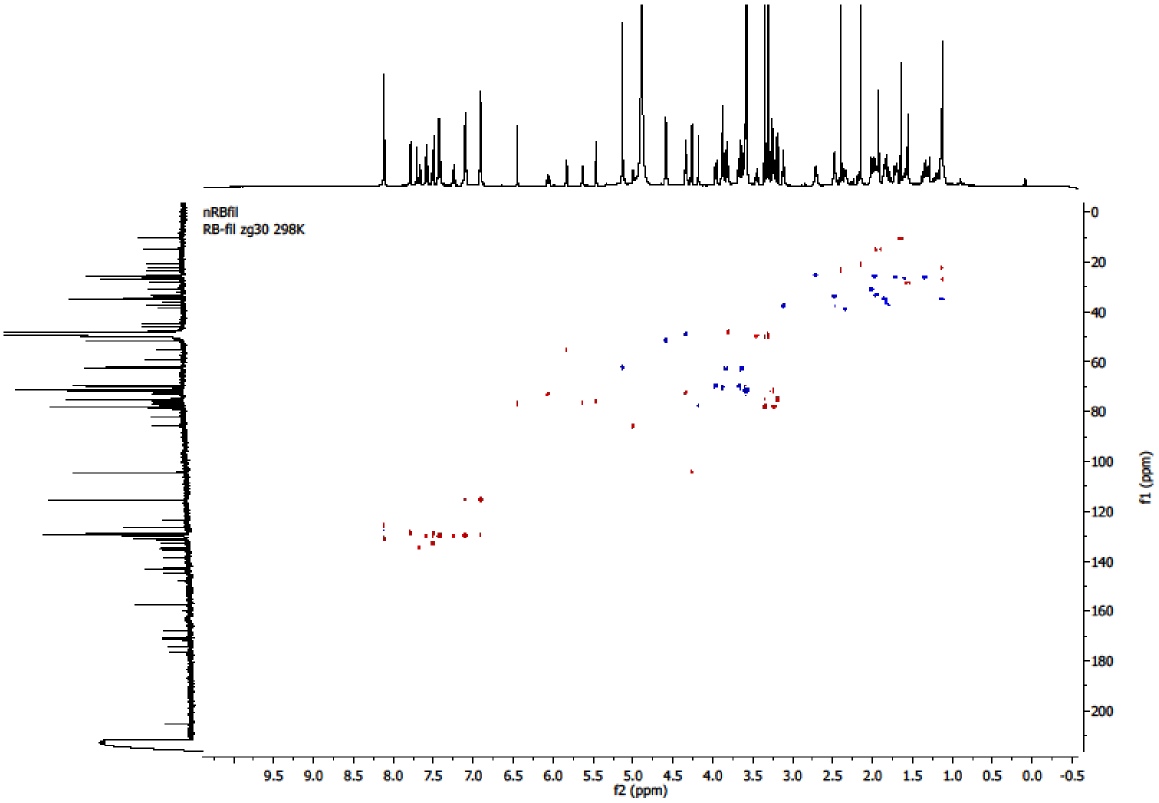


HSQC-edit NMR spectrum (500 MHz, 298 K, $CD_3OD$) of ***4***.

### *Preparation of compound 1 as a mixture of two isomers*

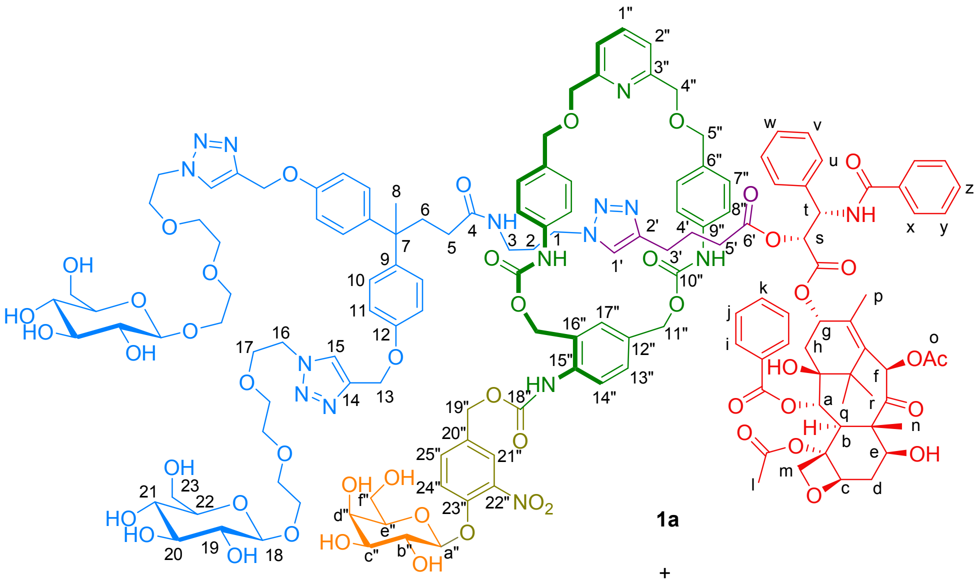

**1a**

+

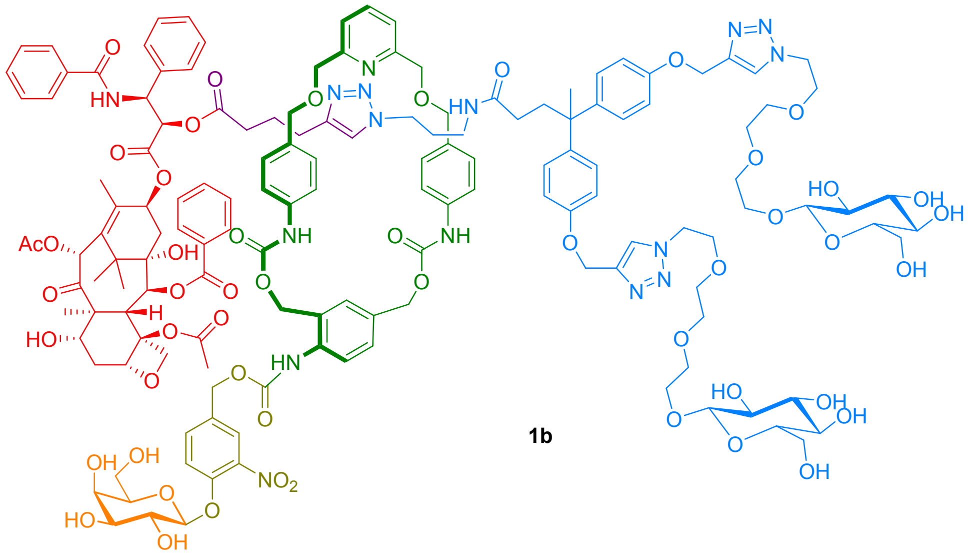

**1b**

The authors draw the reader's attention that rotaxanation step must be performed in a dry schlenk tube, under argon atmosphere with anhydrous solvents freshly degassed.

Macrocycle **8** (10 mg, 11.0 $\mu$mol) and $[Cu(CH_3CN)_4](PF_6)$ (3.7 mg, 9.9 $\mu$mol, 0.9 equiv.) were stirred in a solution of $CH_2Cl_2$/MeOH 83/17 (0.8 mL) at room temperature. A solution of azide **9** (49.2 mg, 44.0 $\mu$mol, 1 equiv.) in $CH_2Cl_2$/MeOH 4/1 (0.25 mL) and alkyne **10** (41.6 mg, 44.0 $\mu$mol, 4 equiv.) were added and stirring was continued for 45 hours at room temperature. EDTA was added and the mixture stirred for 1 hour. The organic layer was separated and the aqueous phase was extracted with $CH_2Cl_2$/MeOH 9:1. The combined

organic layers were dried over $MgSO_4$, filtered and concentrated under reduced pressure. The resulting crude material was purified by semi-preparative reverse-phase HPLC. Crude compound (7x10 mg) was dissolved in a minimum amount of MeOH and filtered through a syringe filter (membrane Nylon, porosity 0.45$\mu$m, diameter 13 mm). Solvent flow 4 mL.min$^{-1}$ was applied to a semi-preparative column ACE® C18-AR (100x10 mm, 5 $\mu$m). Gradient eluent was composed of A ($H_2O$) and B ($CH_3CN$). Method: linear gradient beginning with A/B 80:20 reaching A/B 0:100 within 30 minutes. After lyophilization, compound **1** was obtained as a white powder (9.0 mg after 7 runs, 3.0 $\mu$mol, 27%). In this case 4.5 mg of starting macrocycle **8** was recovered and 9 mg of the thread **4** was isolated.

**$^1$H NMR** of a mixture of two isomers (500 MHz, $CD_3OD$). Protons from the macrocycle are indicated in blue and protons from the thread are indicated in red:

δ 1.09-1.14 (m, 6H, 3$H_q$, 3$H_r$), 1.46-1.56 (m, 3H, 3$H_8$), 1.65 (m, 3H, 3$H_n$), 1.74-2.04 (m, 13H, 2$H_h$, 3$H_o$, 2$H_2$, 2$H_5$, 2$H_{2'}$, 2$H_{5'}$), 2.11 (s, 3H, 3$H_p$), 2.29-2.36 (m, 2H, 2$H_6$), 2.40-2.41 (m, 3H, 3$H_l$), 2.46-2.49 (m, 2H, 2$H_d$), 2.57-2.70 (m, 2H, 2$H_{3'}$), 3.10-3.28 (m, 7H, 5$H_{glucose}$, 2$H_3$), 3.35-3.38 (m, 3H, 3$H_{glucose}$), 3.57-3.60 (m, 14H, 1$H_{c''}$, 1$H_{d''}$, 12$H_{CH2\ PEG}$), 3.63-3.70 (m, 4H, 2$H_{23}$, 2$H_{CH2\ PEG}$), 3.72-3.76 (m, 2H, 2$H_{f''}$), 3.82-3.91 (m, 9H, 1$H_b$, 2$H_{CH2\ PEG}$, 4$H_{16}$, 1$H_{b''}$, 1$H_{e''}$), 3.94-3.98 (m, 2H, 2$H_{23}$), 4.19 (s, 2H, $H_m$), 4.28 (d, $J$ = 7.8, 2H, $H_{18}$), 4.33-4.59 (m, 15H, 2$H_1$, 4$H_{17}$, 1$H_e$, 4$H_{4''}$, 4$H_{5''}$), 4.98-5.03 (m, 2H, $H_{a''}$, $H_c$), 5.11-5.21 (m, 10H, 4$H_{13}$, 4$H_{11''}$, 2$H_{19''}$), 5.46-5.49 (m, 1H, $H_s$), 5.62-5.64 (m, 1H, $H_a$), 5.84-5.86 (m, 1H, $H_t$), 6.03-6.07 (m, 1H, $H_g$), 6.41-6.44 (m, 1H, $H_f$), 6.87-7.09 (m, 15H, 4$H_{10}$, 4$H_{7''}$, 4$H_{11}$, 1$H_{17''}$, 2$H_v$), 7.25-7.27 (m, 2H, 1$H_{13''}$, 1$H_z$), 7.41-7.61 (m, 16H, 1$H_w$, 1$H_{24''}$, 2$H_j$, 1$H_{25''}$, 2$H_{2''}$, 1$H_{14''}$, 4$H_{8''}$, 2$H_x$, 2$H_y$), 7.66-7.67 (m, 1H, $H_{1'}$), 7.68-7.69 (m, 1H, $H_K$), 7.78-7.85 (m, 3H, 2$H_u$, 1$H_{21''}$), 8.09-8.12 (m, 5H, 2$H_{15}$, 2$H_i$, 1$H_{1''}$).

**$^{13}$C NMR** of a mixture of two isomers (125 MHz, $CD_3OD$). Carbons from the macrocycle are indicated in blue and carbons from the thread are indicated in red:

δ 10.5 ($C_n$), 15.0-15.3 ($C_o$), 20.8-20.9 ($C_p$), 22.4 ($C_q$), 23.3 ($C_l$), 25.1-25.2 ($C_{3'}$), 25.6 ($C_{CH2}$), 27.0 ($C_r$), 28.3 ($C_8$), 30.7 ($C_{CH2}$), 33.0 ($C_{CH2}$), 33.6-33.7 ($C_d$, $C_6$), 36.5 ($C_{quat.\ paclitaxel}$, $C_{CH2}$), 37.5 ($C_3$, $C_h$), 38.6 ($C_6$), 44.6 ($C_{quat.\ paclitaxel}$), 45.8 ($C_7$), 47.9 ($C_b$), 49.0 ($C_1$), 51.5 ($C_{17}$), 55.3-55.4 ($C_t$), 59.2-59.3 ($C_{quat.\ paclitaxel}$), 62.4 ($C_{f''}$, $C_{13}$), 62.7 ($C_{CH2\ PEG}$), 64.9-66.5 ($2C_{11''}$), 69.7 ($C_{23}$), 70.1 ($C_{d''}$), 70.3 ($C_{16}$), 71.3 ($3C_{CH2\ PEG}$), 71.6 ($C_{glucose}$), 72.0 ($C_{b''}$), 72.4 ($C_e$), 73.0 ($C_g$), 73.1-73.9-74.0 ($C_{4''}$, $C_{5''}$, $C_{19''}$), 74.8 ($C_{c''}$), 75.0 ($C_{glucose}$), 75.9 ($C_s$), 76.3 ($C_a$), 76.8 ($C_f$), 77.3-77.5 ($C_{e''}$, $C_m$), 78.0-79.0 ($2C_{glucose}$), 82.3 ($C_{quat.\ paclitaxel}$), 85.9 ($C_c$), 103.1 ($C_{a''}$), 104.4 ($C_{18}$), 115.4 ($C_{10}$), 119.0 ($C_{24''}$), 120.0 ($C_{7''}$), 123.6-123.7 ($C_{14''}$, $C_{2''}$, $C_{1'}$), 125.9 ($C_{21''}$), 126.2 ($C_{15}$), 128.6 ($C_u$, $C_w$), 129.5-129.6-129.7 ($C_{13''}$, $C_{17''}$, $C_v$, $C_y$, $C_z$, $C_{11}$), 130.1-130.2 ($C_{8''}$, $C_{quat.\ aro.\ paclitaxel}$), 131.2 ($C_i$), 131.4 ($C_{quat.\ aro.\ paclitaxel}$), 132.3-132.8-132.9 ($C_{6''}$, $C_{9''}$, $C_{15''}$, $C_{20''}$), 133.0 ($C_x$), 134.7 ($C_{25''}$, $C_k$), 134.9 ($C_j$), 135.5-138.4-139.1-139.7-141.8-142.4-148.0-151.2 ($C_{12''}$, $C_{16''}$, $C_{22''}$, $C_{23''}$, $C_{2'}$, $C_9$, $2C_{C=C\ paclitaxel}$, $C_{quat.\ aro.\ paclitaxel}$), 143.1 ($C_{1''}$), 145.0 ($C_{14}$), 155.5-155.6-156.8-159.4 ($C_{3''}$, $3C_{C=O\ carbamate}$), 157.7 ($C_{12}$), 176.4 ($C_4$), 167.7-170.5-170.9-171.4-171.7-174.1 ($C_{6'}$, [$2C_{C=O\ acetate}$, $2C_{C=O\ esters}$, $C_{C=O\ amide}$]$_{paclitaxel}$), 205.2 ($C_{C=O\ paclitaxel}$).

**HRESI-MS**: m/z 2978.1807 (calcd. for $C_{148}H_{177}N_{16}O_{50}$ 2978.1721 $[M+H]^+$).

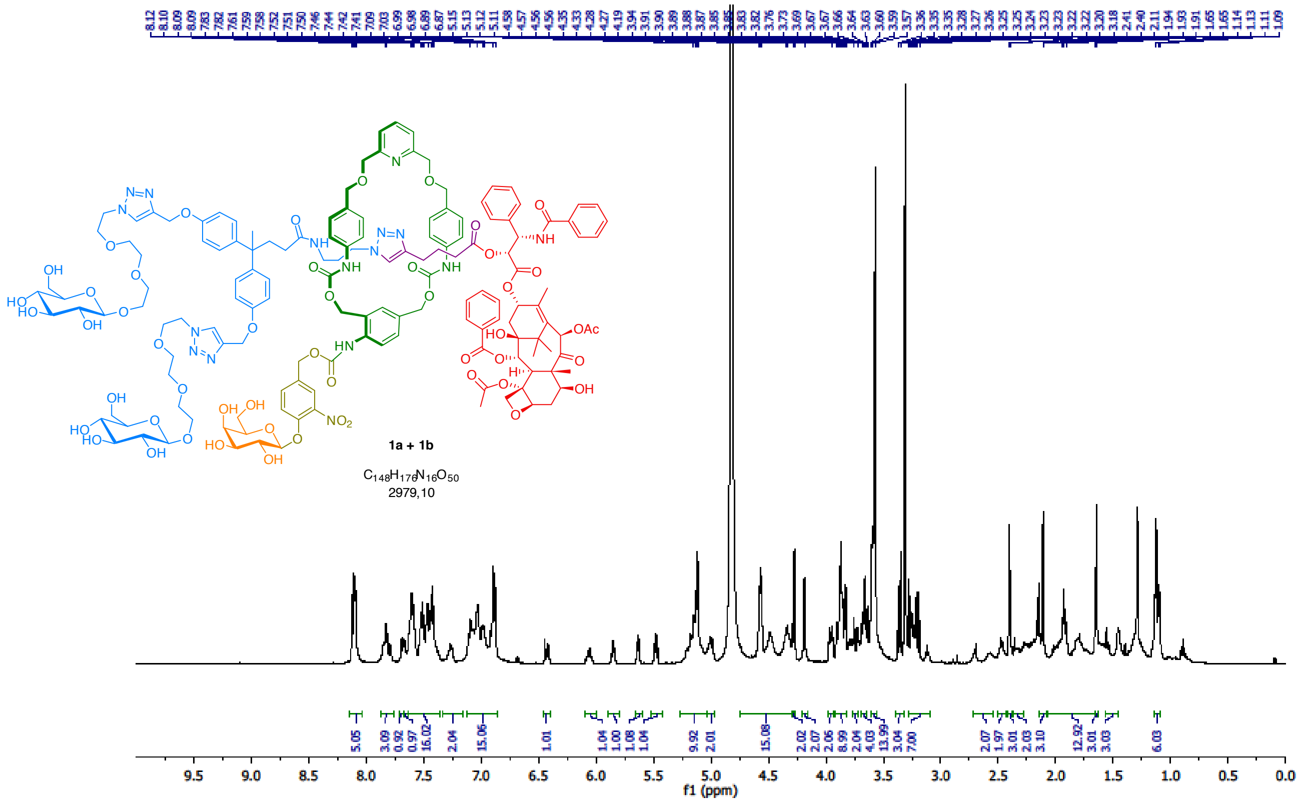

$^{1}$H NMR spectrum (500 MHz, 298 K, $CD_3OD$) of **1**.

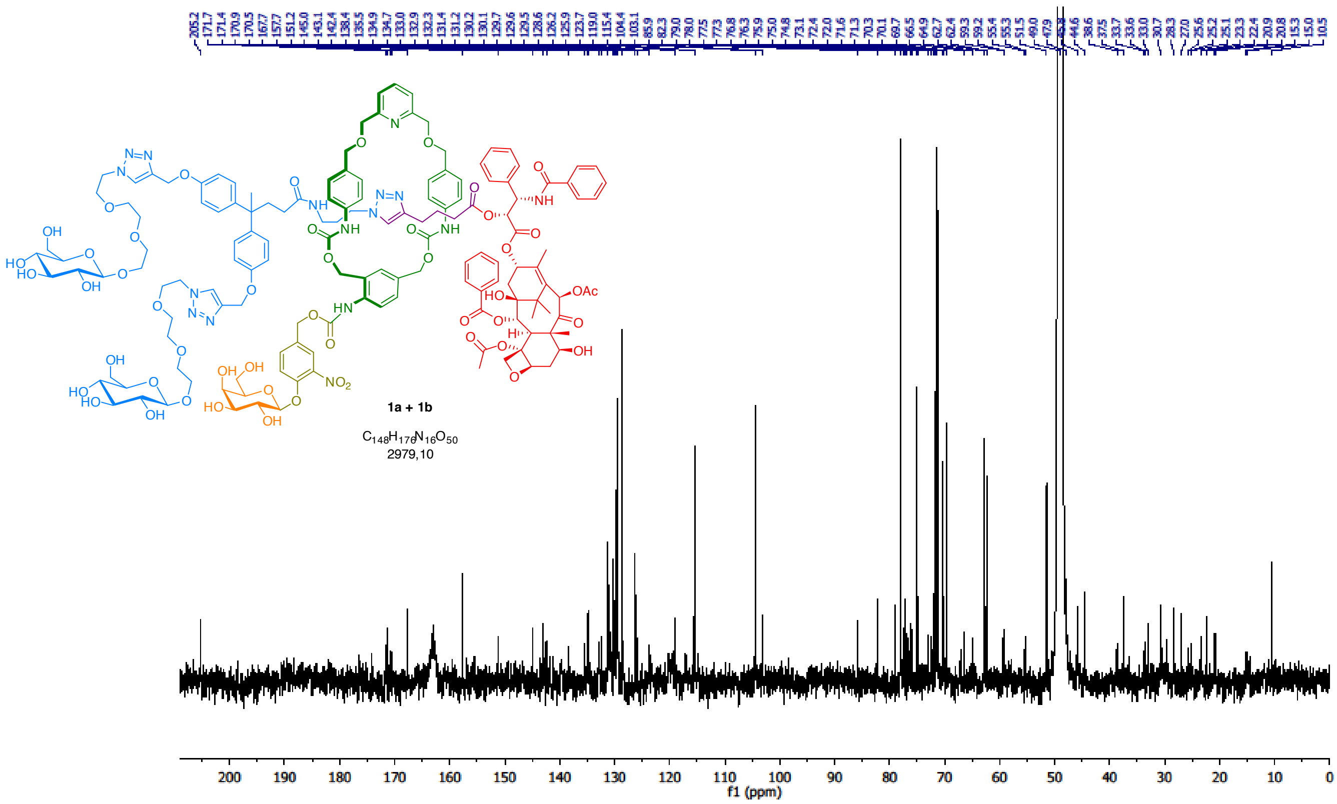

$^{13}$C NMR spectrum (125 MHz, 298 K, $CD_3OD$) of **1**.

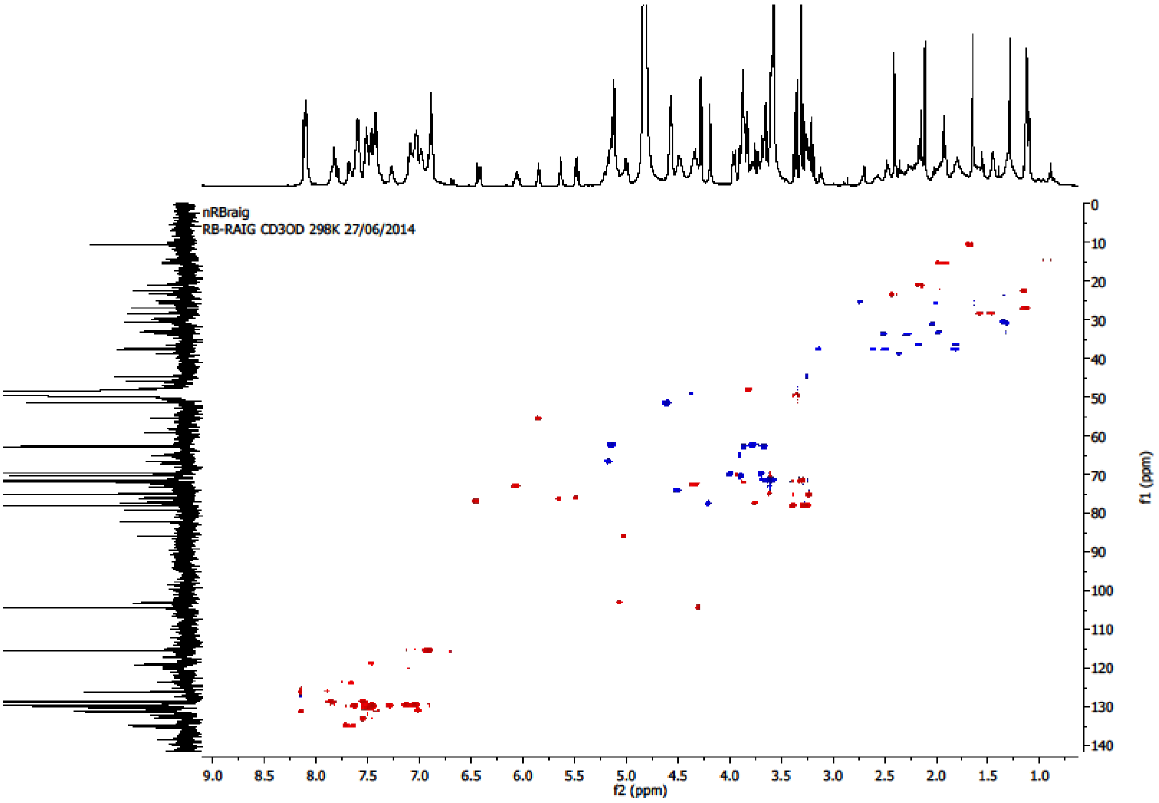


HSQC-edit NMR spectrum (500 MHz, 298 K, $CD_3OD$) of **1**.

| 329 ROTAXANE AI GAL | | | |
|---|---|---|---|
| *Sample Name:* | RB2p176 8-28 | *Injection Volume:* | 20,0 |
| *Vial Number:* | 386 | *Channel:* | UV_VIS_1 |
| *Sample Type:* | unknown | *Wavelength:* | 254 |
| *Control Program:* | rotaxanation | *Bandwidth:* | n.a. |
| *Quantif. Method:* | default | *Dilution Factor:* | 1,0000 |
| *Recording Time:* | 22/01/2014 17:47 | *Sample Weight:* | 1,0000 |
| *Run Time (min):* | 35,00 | *Sample Amount:* | 1,0000 |

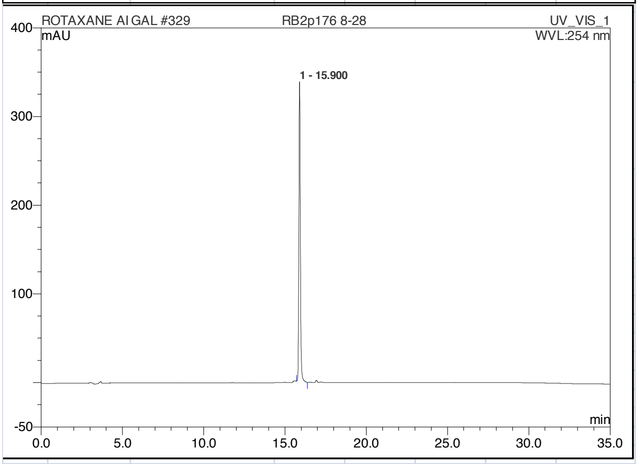


| No. | Ret.Time min | Peak Name | Height mAU | Area mAU*min | Rel.Area % | Amount | Type |
|---|---|---|---|---|---|---|---|
| 1 | 15,90 | n.a. | 338,038 | 36,299 | 100,00 | n.a. | BMB |
| Total: | | | 338,038 | 36,299 | 100,00 | 0,000 | |

HPLC chromatogram of **1** (as a mixture of txo isomers **1a** and **1b**) after semi-preparative reverse phase HPLC purification

## I.4. β-galactosidase cleavage

Enzymatic hydrolysis was carried out with commercial β-galactosidase from *Escherichia coli* E.C. 3.2.1.23 (768 units/mg protein (biuret), suspension in 50% glycerol, 10 mM Tris buffer salts and 10 mM magnesium chloride, pH 7.3). The rotaxane **1** (0.24 mg, 0.08 $\mu$mol) was incubated at 37°C with the enzyme (750 U.$\mu$mol$^{-1}$) in a solution of 20 mM phosphate buffer at pH 7.0 (0.5 mL) containing 10% of DMSO. Hydrolysis was monitored by analytical HPLC using a linear gradient composed of A (0.2% TFA in water) and B ($CH_3CN$) beginning with A/B 80:20 and reaching A/B 0:100 within 30 minutes. (Figure S6). Intermediates **1** to **4** were identified by UHPLC-HRMS (Q-Exactive) using water as mobile phase A and acetonitrile as mobile phase B, both containing 0.1% formic acid with the same elution gradient (Figure S7).

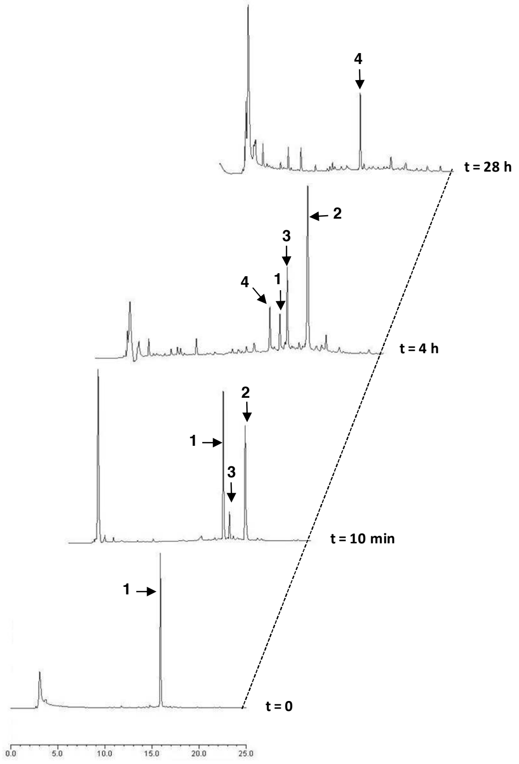


**Fig. S6.** HPLC chromatograms: enzymatic hydrolysis of **1** with *E. coli* β-galactosidase in phosphate buffer (20 mM, pH 7.0) at 37°C using 750 U.$\mu$mol$^{-1}$ of substrate. Retention time: **1**: 15.87 min., **2**: 18.22 min., **3**: 16.59 min., **4**: 14.98 min.

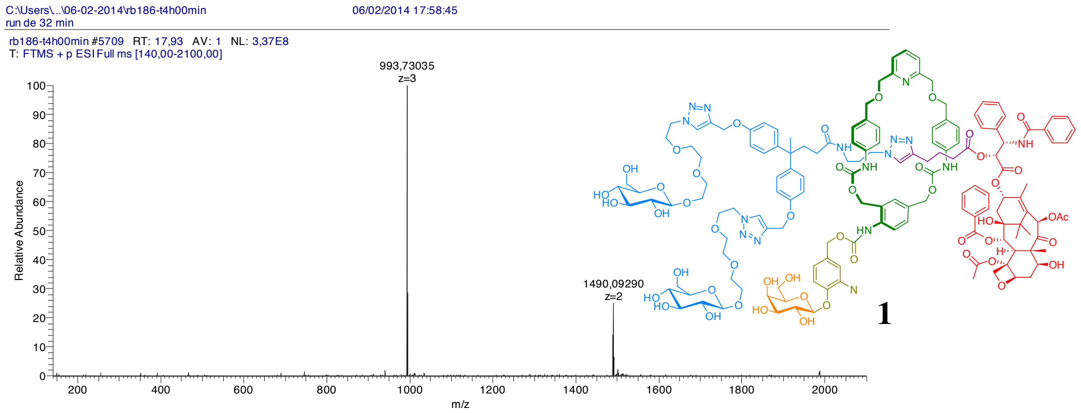


**1**

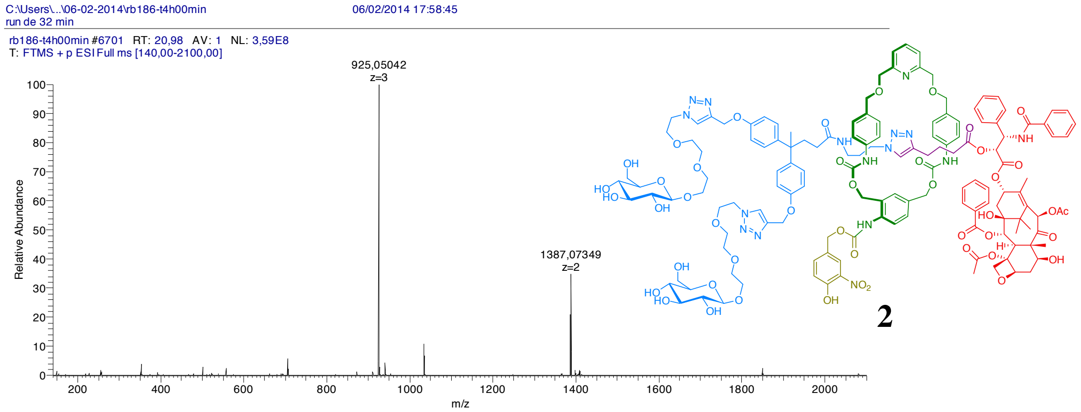


**2**

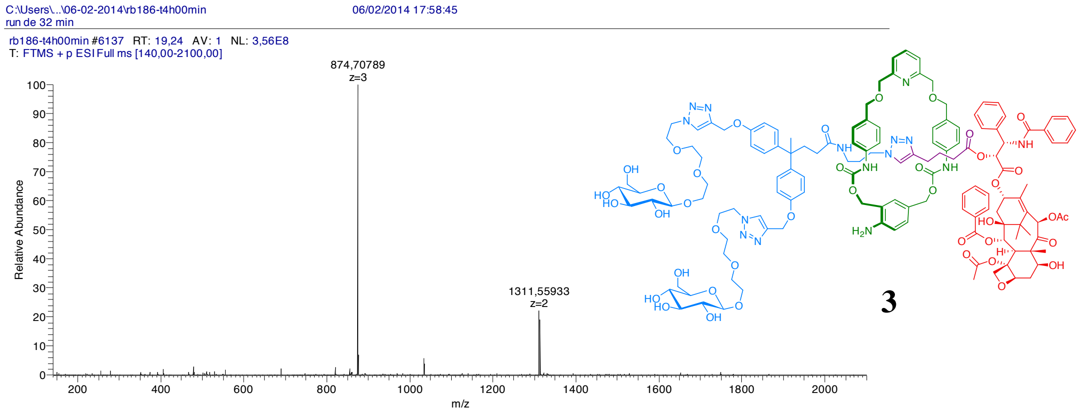


**3**

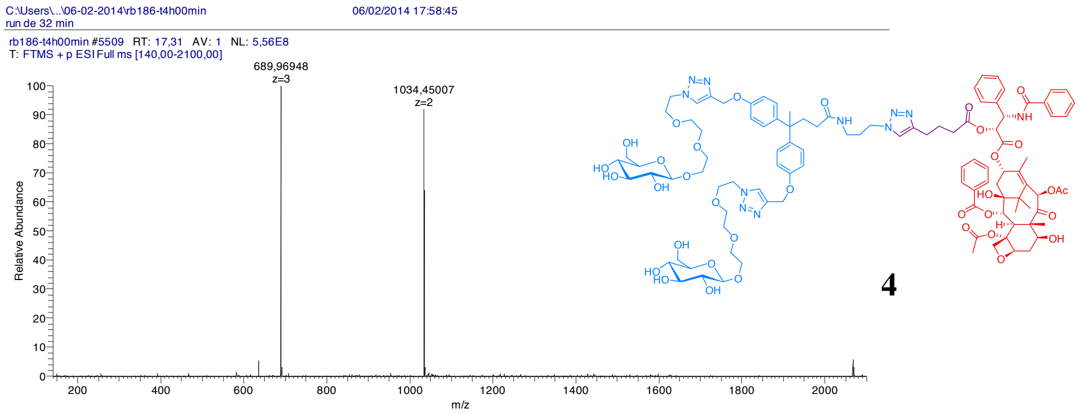


**4**

**Fig. S7.** Mass spectra of compounds **1**, **2**, **3**, **4**.

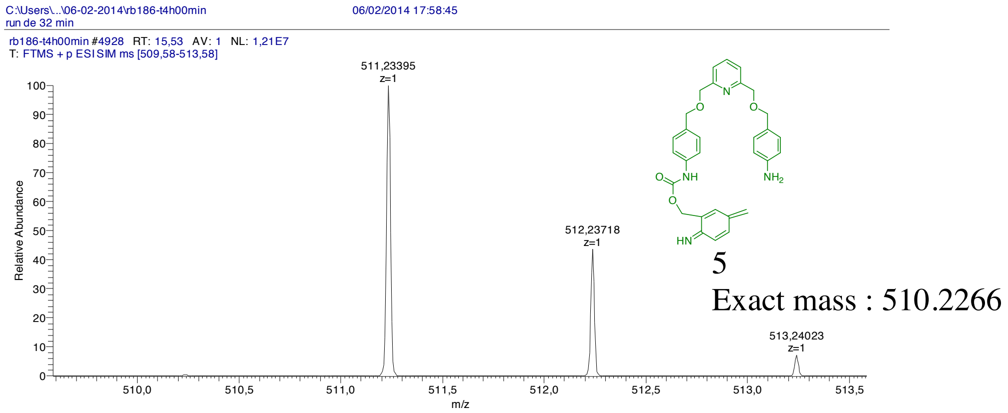


**Fig. S8.** Selected ion monitoring of intermediate **5**.

## I.5. Stability toward rat plasma esterase

Rotaxane **1** (0.1 mg, 0.033 $\mu$mol) and thread **4** (0.13 mg, 0.062 $\mu$mol) were separately incubated at 37°C in identical freshly collected rat plasma (1.0 mL). Aliquots (50 $\mu$L) were periodically withdrawn from the medium, poured into cold MeOH (100 $\mu$L) to precipitate the proteins and cooled on ice. After 30 minutes, the sample was centrifuged (9000 rpm, 5 minutes) and the supernatant analyzed by analytical HPLC using a linear gradient composed of A (0.2% TFA in water) and B ($CH_3CN$) beginning with A/B 80:20 and reaching A/B 0:100 within 30 minutes. HPLC analysis showed no detectable degradation of rotaxane **1** during 48 hours (Figure S9a) and degradation of thread **4** with a release of paclitaxel (Figure S9b) under these conditions.

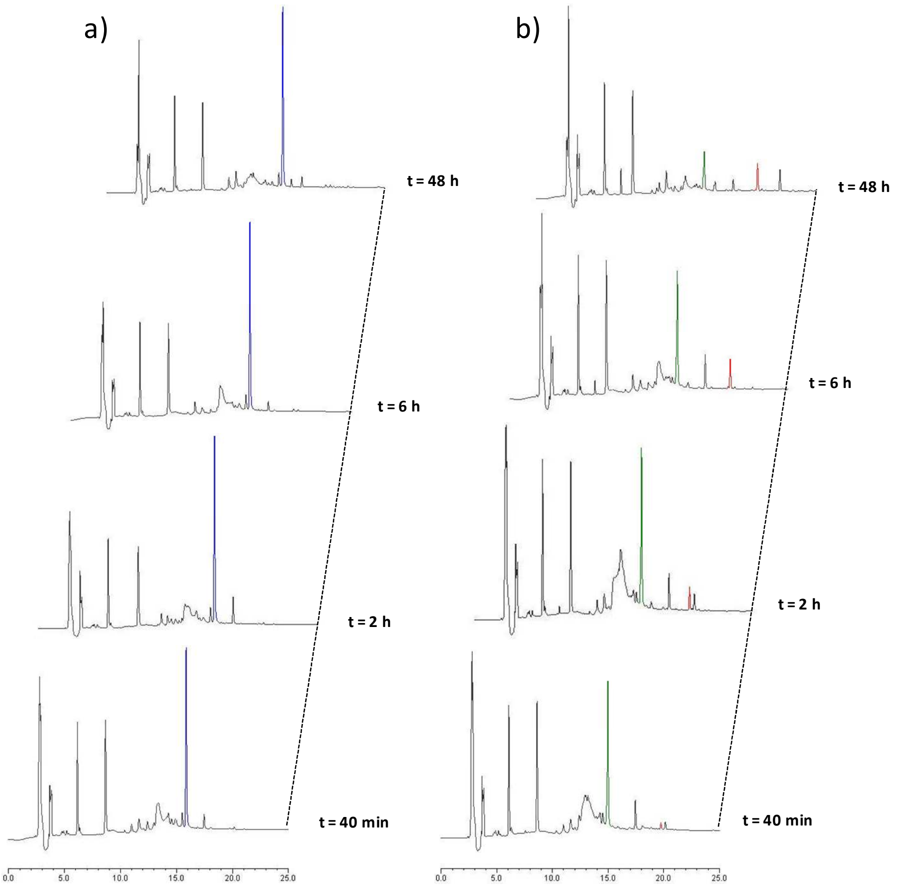


**Fig. S9.** HPLC chromatograms: (a) stability of rotaxane **1** (blue peak) in rat plasma (black peaks); (b) stability of thread **4** (green peak) in rat plasma (black peaks) with release of paclitaxel ( red peak).

# II. Biological Section

## II.1. Cell culture

KB (human oral carcinoma), MDA-MB-231 (human breast cancer) and H661 (human lung carcinoma) cells were maintained in RPMI 1640-GlutaMAX (Gibco) supplemented by 10 % fetal bovine serum and 1 % Penicillin/Streptomycin (Lonza) in a humidified incubator at 37 °C and 5 % $CO_2$. Human umbilical vein endothelial cells (HUVECs, Lonza) were cultured in EGM-2 (endothelial growth medium-2) composed by endothelial cell basal medium-2 (EBM-2) and EGM-2 SingleQuot Kit Suppl. & Growth Factors (FBS, hydrocortisone, hFGF, VEGF, R3-IGF-1, ascorbic acid, hEGF, GA-1000 and heparin). HUVECs were used at early passages (1 to 8). Cells were kept at 37 °C in a 5% $CO_2$ humidified incubator.

## II.2. Cell viability

The Cell Proliferation Kit II XTT (Roche) was used to measure cell viability. For this assay, cells were seeded in 96-well plate. After 24 h, cells were exposed to Paclitaxel or **1** at the indicated concentration. After 2 days of treatment, 25 $\mu$l of the XTT labeling mixture were added per well. After additional 4 h of incubation, absorbance was determined at 490 nm. Experiments were performed 2 times in triplicate. Data were analyzed with GraphPad software. For each compound, inhibitory concentration values ($IC_{50}$) were determined by the software.

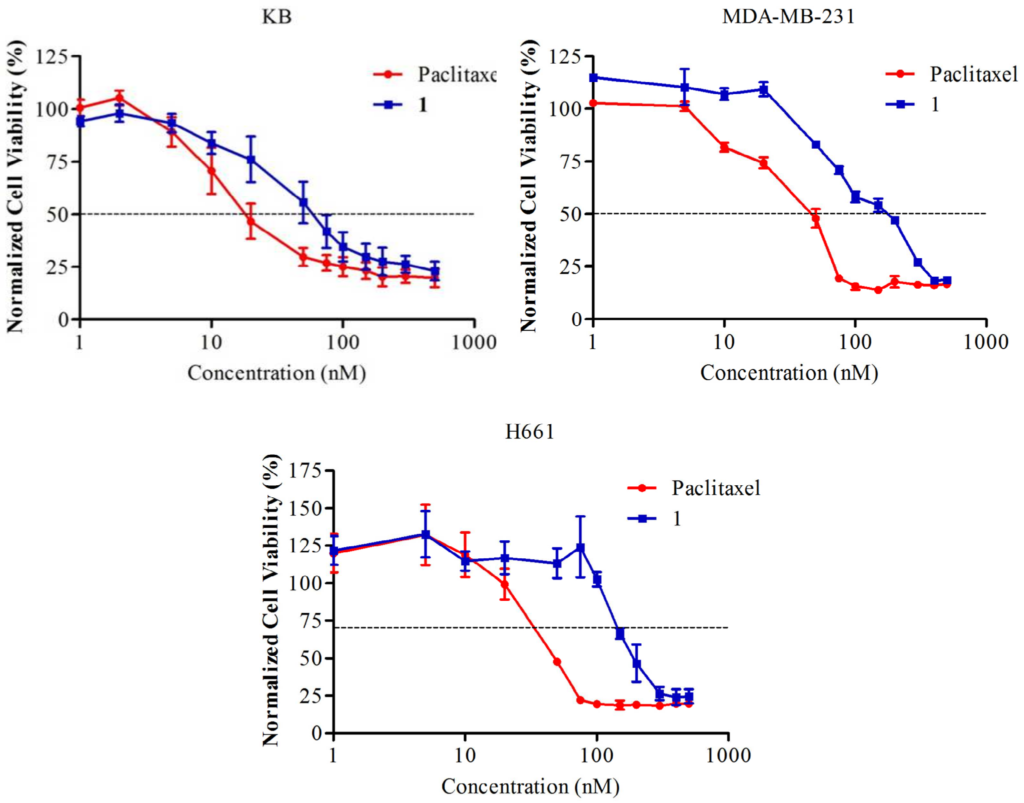


**Fig. S10.** Cell viability of KB, MDA-MB-231 and H661 cells after 2 days of treatment with different concentrations of Paclitaxel or **1**.

| *IC50 values* | Paclitaxel | **1** |
|---|---|---|
| ***KB*** | 19.4±3.1nM | 88.7±10.3nM |
| ***MDA-MB-231*** | 41.2±5.0nM | 192.3±19.1nM |
| ***H661*** | 37.3±8.0nM | 144±3.0nM |

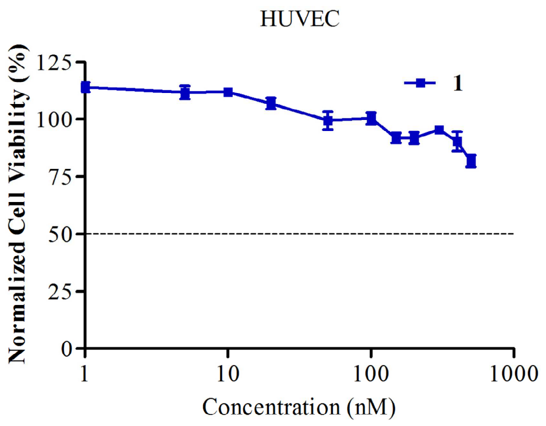


**Fig. S11.** Cell viability of HUVEC cells after 2 days of treatment with different concentrations of **1**.

## II.3. Immunofluorescence

Immunodetection of α-tubulin was performed on KB cells that were fixed in cold methanol (-20°C) for 10 min. Cells were incubated with a monoclonal anti-α-tubulin antibody (1:500) at room temperature (RT) for 30 min. Alexa Fluor® 488 goat anti-mouse (1:50, Invitrogen, excitation/emission wavelengths of 488 and 590 nm) antibody was then applied at RT for 30 min. Cover slips were mounted with Mowiol (Calbiochem) prior to observation with a confocal microscope (FV 1000, Olympus IX-81). Scale bar: 25µm.

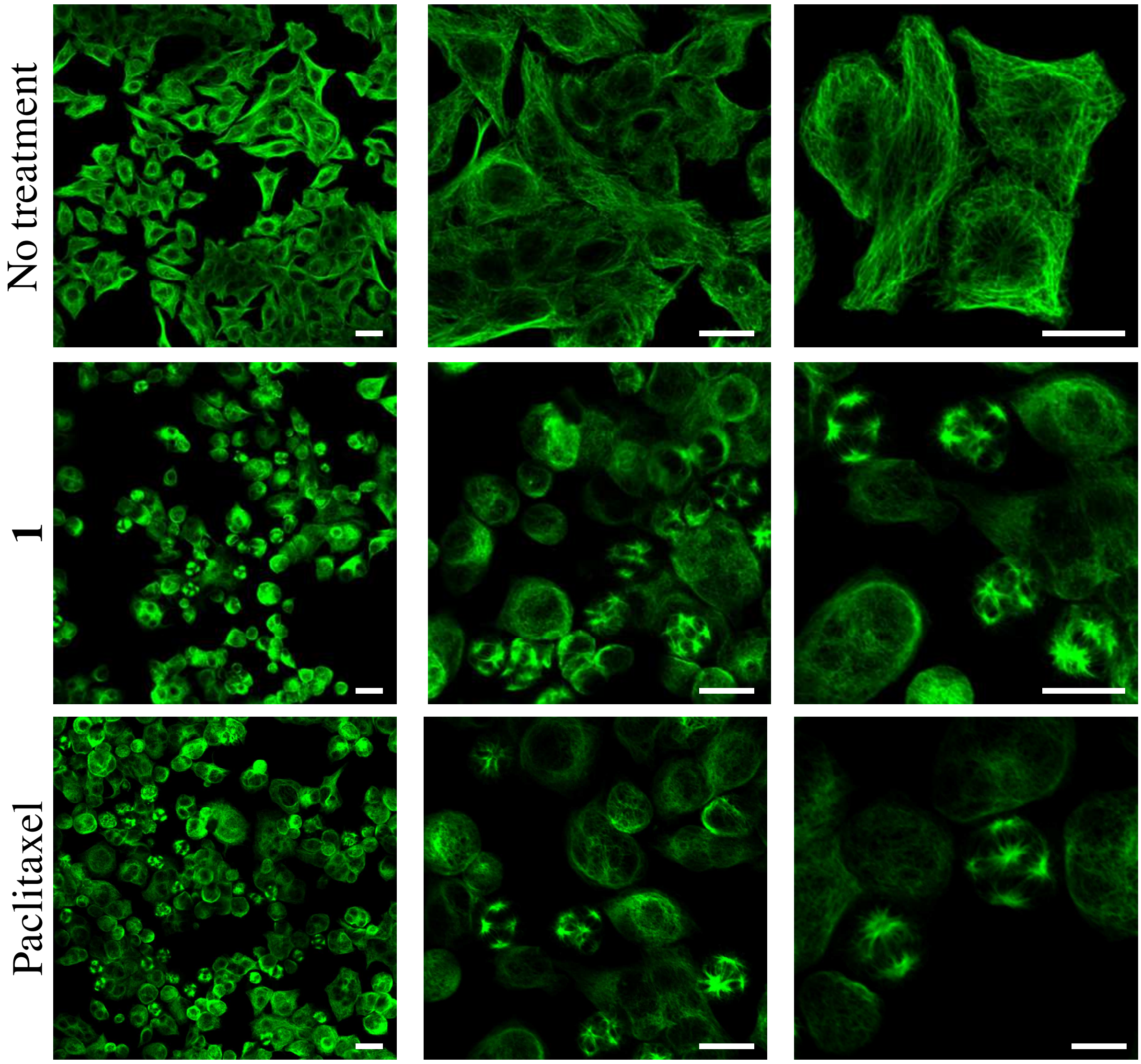


**Fig. S12.** Immunodetection of α-tubulin in KB cells after 24h of treatment with 100 nM of **1** or 25 nM of Paclitaxel.

## II.4. Small Interfering RNA Transfection

### *β-Galactosidase expression inhibition*

To inhibit β-Galactosidase expression, RNA interference silencing was performed using siPORT NeoFX transfection agent (Ambion) according to the manufacturer's instructions. Pre-designed β-Galactosidase siRNAs, called siGLB1, were purchased from Ambion (sense: 5'-GCUACUUUGCCUGUGAUUUtt-3'; antisense: 5'-AAAUCACAGGCAAAGUAGCtg-3'). Silencing control was performed by transfection of a scramble siRNA (Ambion) with no significant homology to any known gene sequence from human, rat and mouse. Briefly, 2 x$10^5$ KB cells were transfected with 30 nM of siRNA in a 6-well plate. Cells were incubated at 37 °C for 2 days before RNA extraction using RNA Total Isolation Kit (Promega) as previously described.[6] Reverse transcription was done with 1 to 1.5 $\mu$g of RNA using qScript cDNA Synthesis Kit (Quanta Biosciences), according to the manufacturer's instructions. β-Galactosidase gene expression was assessed relative to GAPDH by quantitative real-time PCR with the GeneAmp 7500 Sequence Detection System and SYBR Green Chemistry (Applied Biosystems) as previously reported.[6] Specific primers were used for GAPDH (forward: 5'-TGCACCACCAACTGCTTAGC-3' and reverse: 5'-GGCATGGACTGTGGTCATGAG-3') and β-Galactosidase (forward: 5'-CACTCCACAATCAAGACCGAAGC-3' and reverse: 5': CTGTGCTGCATAGGGTGAGTTG-3') quantification. Transfections were performed 2 or 3 times and analyzed in duplicate by RT-qPCR. Two-way ANOVA and Bonferroni post-tests were performed for statistical analysis.

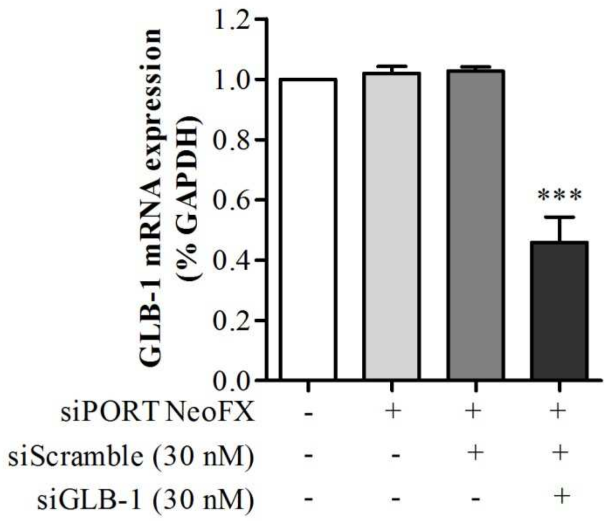


**Fig. S13.** Inhibition of β-Galactosidase (GLB1) expression in siRNA-transfected KB cells.

***Effect of siGLB1 on compound 1- induced toxicity***

RNA interference silencing was performed as described above. Twenty-four hours after transfection, cells were treated with indicated compound (20 nM of Paclitaxel, 100 nM of **1** or **4** and/or 40 units of β-Galactosidase) for 48 additional hours. Cell viability was then assessed using the Cell Proliferation Kit II (XTT; Roche) as described above. Experiments were performed 2 or 3 times. T-tests were performed for statistical analysis.

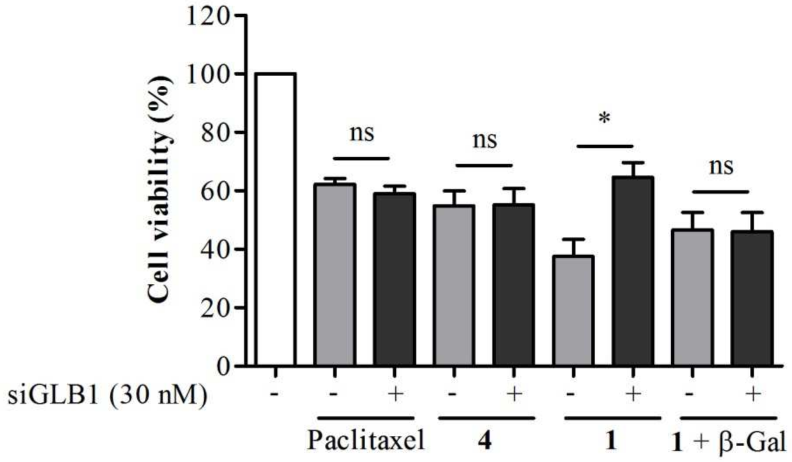


**Fig. S14.** Cell viability of KB cells with β-Galactosidase expression inhibition (siGLB1) after 2 days of treatment with indicated compounds.

# III. References

[1] Erez, R., Shabat, D. The azaquinone-methide elimination: comparison study of 1,6- and 1,4-eliminations under physiological conditions. *Org. Biomol. Chem.* **6**, 2669-72 (2008).

[2] Ozer, H., Kocakaya, S. A. O, Akgun, A., Hosgören, H., Togrul, Ma. The enantiomeric recognition of chiral organic ammonium salts by chiral pyridino-macrocycles bearing aminoalcohol subunits. *Tetrahedron Asym.* **20**, 1541-46 (2009).

[3] Andersen, J., Madsen, U., Björkling, F., Liang, X. Rapid Synthesis of Aryl Azides from Aryl Halides under Mild Conditions. *Synlett.* **14**, 2209-13 (2005)

[4] Sanki, A. K., Mahal, L. K. A One-Step Synthesis of Azide-Tagged Carbohydrates: Versatile Intermediates for Glycotechnology. *Synlett.* **3**, 455-59 (2006).

[5] Hatzakis, N. S., Engelkamp, H., Velonia, K., Hofkens, J., Christianen, P. C. M., Svendsen, A., Patkar, S. A., Vind, J., Maan, J. C., Rowan, A. E., Nolte,R. J. M. Synthesis and Single Enzyme Activity of a Clicked Lipase-BSA Hetero-dimer C*hem. Commun.* **19**, 2012-14 (2006).

[6] Clarhaut, J., Fraineau, S., Guilhot, J., Peraudeau, E., Tranoy-Opalinski, I., Thomas, M., Renoux, B., Randriamalala, E., Bois, P., Chatelier, A., Monvoisin, A., Cronier, L., Papot, S., Guilhot, F. A galactosidase-responsive doxorubicin-folate conjugate for selective targeting of acute myelogenous leukemia blasts. *Leuk. Res.* **37**, 948-55 (2013).
